# DNA base lesion-containing G-quadruplex mediates transcriptome reprogramming in EGFR-TKI resistance of non-small cell lung cancer

**DOI:** 10.1186/s13046-026-03702-w

**Published:** 2026-04-14

**Authors:** Xinming Jing, Zheng Liu, Lujie Yang, Yanli Xiong, Shixun Li, Ruyi Hang, Xiao Yang, Xiaoyan Dai, Jiangyang Li, Jing Yang, Yi Cheng, Ziqi Jiang, Huifen Cao, Philipp Kapranov, Qian Chen, Dong Wang, Wei Guo, Zhou Huang, Mengxia Li

**Affiliations:** 1 Cancer Center, Daping Hospital & Army Medical Center of PLA, Third Military Medical University, Chongqing, China; 2 Thoracic Surgery Department, Daping Hospital & Army Medical Center of PLA, Third Military Medical University, Chongqing, China; 3 State Key Laboratory of Cellular Stress Biology, School of Life Sciences, Xiamen University, Xiamen, China; 4 Institute of Genomics, School of Medicine, Huaqiao University, Xiamen, China; 5 Department of Oncology, General Hospital of Western Theatre Command, Chengdu, China

**Keywords:** Base excision repair, G-quadruplex, EGFR-TKI resistance, Plasticity

## Abstract

**Background:**

Cancer cells, such as non-small cell lung cancer (NSCLC) cells, exhibit remarkable phenotypic plasticity and undergo epigenetic reprogramming, characteristics that enable them to evade targeted therapies. However, how NSCLC cells use epigenetic regulatory mechanisms (including endogenous DNA base damage) to drive transcriptome reprogramming and develop resistance to EGFR-tyrosine kinase inhibitor (EGFR-TKI) therapy remains unclear.

**Methods:**

We employed an integrated multi-omics approach in erlotinib-sensitive and resistant NSCLC cells, including genome-wide mapping of abasic sites (AP sites) via Single-Strand Break Mapping at Nucleotide Genome Level-AP (SSiNGLe-AP), characterization of binding landscapes of 8-Oxoguanine DNA Glycosylase 1 (OGG1), Apurinic/Apyrimidinic Endonuclease 1 (APE1), and genome-wide profiling of G-quadruplex (G4) structures by Cleavage Under Targets and Tagmentation sequencing (CUT&Tag-seq). Combined with RNA-seq and ATAC-seq analysis, we performed functional validation using overexpression, RNAi, qPCR, western blot, immunofluorescence, sphere formation, and flow cytometry. High-resolution microscopy revealed that oxidized base lesions and repair complexes orchestrated the spatiotemporal dynamics of G4 structures. Co-localization of G4 structures with CpG islands (CGIs) was assessed by Whole-Genome Bisulfite Sequencing (WGBS). Finally, the therapeutic relevance of targeting this pathway was evaluated using a mouse xenograft model treated with APE1 inhibitors.

**Results:**

We identified the DNA base lesion repair proteins OGG1 and APE1 as key mediators of transcriptional reprogramming that promote cancer cell plasticity and resistance to EGFR-TKIs. Mechanistically, genome-wide profiling revealed their dynamic redistribution to specific genomic loci in resistant cells. Beyond canonical repair, APE1 stabilizes G4 structures at the promoters of epithelial-mesenchymal transition (EMT) and stemness-associated genes, facilitating their transcriptional activation. This APE1-driven, G4-dependent transcriptional program occurs preferentially at hypomethylated CGIs, revealing a mechanism by which DNA secondary structures shape the epigenetic landscape to promote cellular plasticity.

**Conclusion:**

Our study elucidates a novel pathway in which OGG1/APE1-mediated processing of oxidative damage orchestrates a G4-dependent transcriptional program to drive EMT and stemness in EGFR-TKI-resistant NSCLC. The therapeutic potential of targeting this axis is demonstrated by the efficacy of BER inhibitors in suppressing tumor growth in vivo, establishing APE1-G4 targeting as a promising anti-resistance strategy.

**Supplementary Information:**

The online version contains supplementary material available at 10.1186/s13046-026-03702-w.

## Background

In patients with non-small cell lung cancer (NSCLC) harboring driver mutations, targeted treatment strategies improve the objective response rate (ORR) and greatly prolong progression-free survival (PFS). Epidermal growth factor receptor tyrosine kinase inhibitors (EGFR-TKIs) are employed as the first-line treatment for NSCLC patients with EGFR mutations. EGFR-TKIs, such as gefitinib, erlotinib and osimertinib, are the small-molecule inhibitors that selectively target the mutant EGFR ATP-binding pocket, thereby blocking the constitutive, ligand-independent kinase activity driven by these activating mutations. The most common EGFR mutations in NSCLC include exon 19 deletion and L858R substitution, with TKIs selectively suppressing the proliferation of target cancer cells [1]. However, acquired resistance to EGFR-TKIs develops almost universally and limits the long-term benefits of this treatment modality [2], although the mechanisms underlying resistance are not fully understood.

A key emerging mechanism of therapy resistance is cancer cell lineage plasticity [3], which enables cells to evade lineage-specific targeted therapies by adopting alternative identities. In particular, the epithelial-mesenchymal plasticity of cancer cells plays a pivotal role in tumor invasion, acquisition of stemness, and metastasis [4]. However, epithelial-to-mesenchymal transition (EMT) is not only a change in cell morphology but also an ‘upside-down’ change in gene expression [5, 6]. Although numerous transcription factors, including Snail, ZEB1, Twist, and FoxC2, have been implicated in EMT [7–9], the mechanisms driving the dramatic changes in gene expression during and after EMT remain incompletely understood. Critically, no therapies are currently available to specifically counter resistance driven by lineage plasticity.

At the molecular level, G-quadruplexes (G4s), i.e., non-B-form DNA secondary structures within specific guanine (G)-rich sequences, mainly found in nucleosome-depleted chromatin regions such as gene promoters, are increasingly recognized as important regulatory elements in the genome [10, 11]. Potential G4‑forming sequences (PQS) are G-rich DNA motifs that can fold into G4 structures to regulate genomic stability and gene expression. The guanine bases within PQS are susceptible to oxidative damage, resulting in the formation of 8-oxo-7,8-dihydroguanine (8-oxo-G), a common oxidative base lesion in the genome [12]. This damage is mainly repaired via the base excision repair (BER) pathway, initiated by 8-Oxoguanine DNA Glycosylase 1 (OGG1) and processed by (Apurinic/Apyrimidinic Endonuclease 1) APE1 [13]. Notably, oxidative damage within G4s is not merely a form of genomic insult; evidence suggests that 8-oxo-G and the ensuing BER intermediates can function as epigenetic-like marks, actively promoting the transcription of nearby genes [14]. Despite its potential significance, the biological and clinical relevance of this oxidative damage-mediated regulation in cancer plasticity and therapy resistance is largely unexplored.

To examine potential relationships between DNA damage, G4 structures prone to oxidation, and core BER enzymes—the key mediators of the base excision repair pathway, which resolves oxidative DNA damage—we performed genome-wide unbiased mapping of G4 structures and the binding sites of APE1 and OGG1, as well as cross-referencing and detection of DNA base lesions in tumor plasticity-related signatures. In this study, we focus specifically on two key effectors of the BER pathway: OGG1 and APE1.We hypothesized that endogenous oxidative damage at G4 sites, processed by OGG1 and APE1, serves as a regulatory mechanism that orchestrates transcriptome reprogramming to drive cellular plasticity and resistance. This study provides comprehensive evidence that APE1/OGG1-mediated processing of oxidative damage at G4 structures controls the transcriptional programs underlying EMT and stemness- associated phenotypes, thereby establishing a novel epigenetic-like mechanism in NSCLC acquired resistance.

## Materials and methods

All materials and methods are included in Extended Data Materials and Methods.

## Results

### Oxidative DNA damage accumulation and BER activation in EGFR-TKI resistant NSCLC cells

**Fig. 1:**
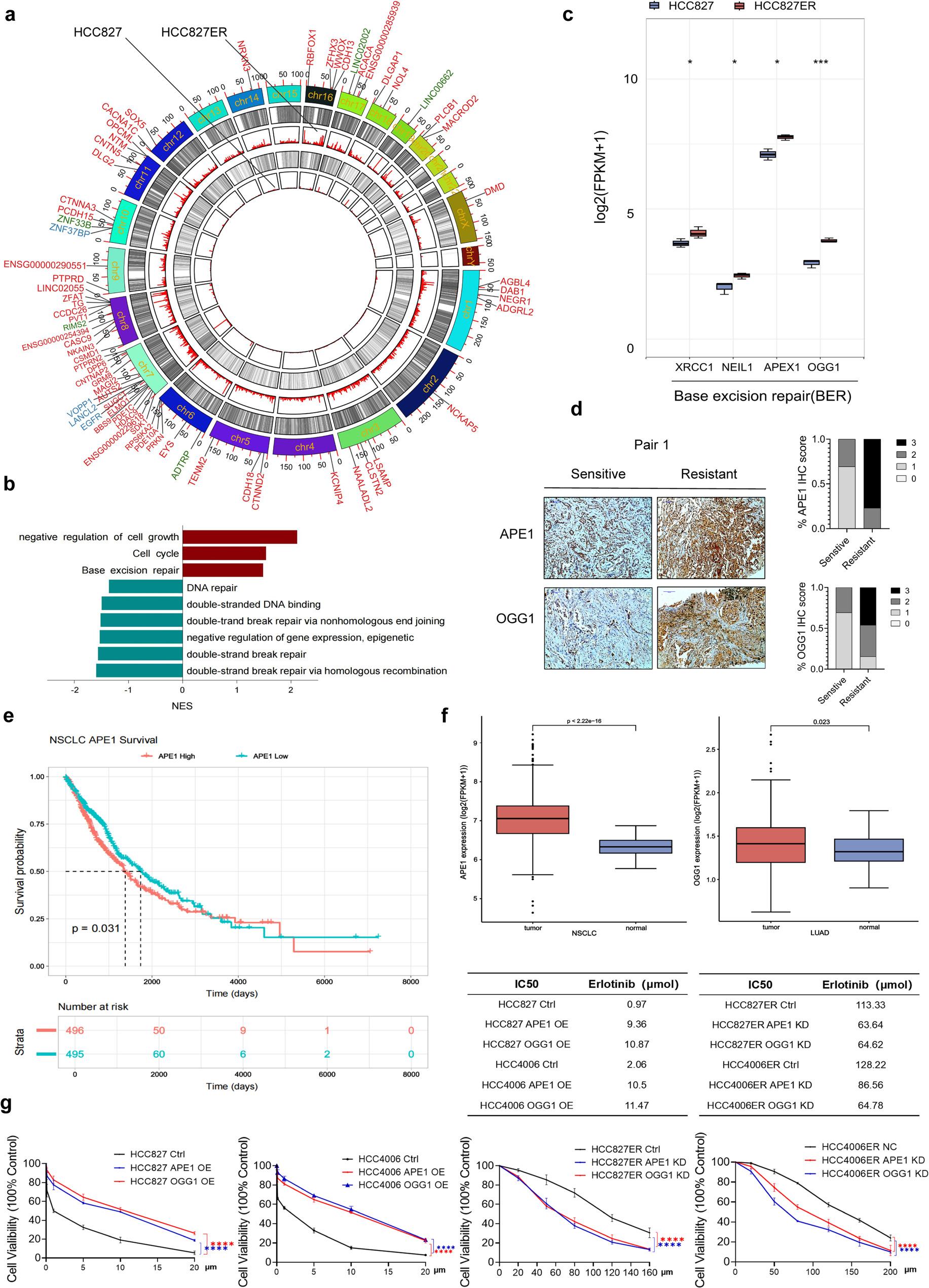
Oxidative DNA damage accumulation and BER activation in EGFR-TKI resistant NSCLC cells. **a**, Genomic distribution of AP sites in HCC827 and HCC827ER cells. The concentric rings (outer to inner) represent: chromosomes, HCC827-specific AP sites, gene-mapped AP site counts for HCC827, HCC827ER-specific AP sites, and gene-mapped AP site counts for HCC827ER. The outermost ring labels genes with ≥30 AP sites (color-coded: red, HCC827ER; blue, HCC827; green, shared). **b**, GSEA pathway analysis highlighting cancer-related signaling pathways associated with altered DNA repair in HCC827ER cells compared with parental cells. **c**, Differential expression analysis of BER pathway genes in HCC827ER vs. parental cells. **d**, Representative images of IHC analysis of the expression of OGG1 and APE1 in 13 pairs of human NSCLC tissue samples before and after resistance to first- and second-generation EGFR-TKI treatment (Scale bar, 100 µm). **e**, Elevated APE1 expression significantly correlates with reduced overall survival in the TCGA-LUAD cohort (log-rank p=0.031). **f**, APE1 and OGG1 were significantly upregulated in NSCLC tissues compared to normal controls by analyzing TCGA-derived clinical samples. **g**, Erlotinib dose-response curves for control and APE1 or OGG1 OE in parental cell lines and control and APE1 or OGG1 KD in ER counterparts. IC₅₀ values of sensitive and resistant cell lines. n=3 experimental replicates, mean ± SEM. Significance was calculated using a paired t test. *p < 0.05

Two NSCLC cell lines with EGFR exon 19 deletion (HCC827 and HCC4006) were selected to establish in vitro acquired drug resistance models via long-term exposure to incrementally increasing concentrations of erlotinib, a first-generation EGFR tyrosine kinase inhibitor (EGFR-TKI). The parental cell lines showed erlotinib IC₅₀ values of 2 µM or lower, while the resistant variants exhibited more than 50-fold increases in IC₅₀ (Extended Data Fig. 1a, b). Isolated erlotinib-resistant (ER) cells (HCC827ER and HCC4006ER) lost their initial epithelial morphology and displayed a more ‘spindle-like’ morphological organization, analogous to that of mesenchymal cells (Extended Data Fig. 1a). Next, to characterize the genomic profiles of oxidative DNA damage in parental and resistant cells, we employed a high-resolution Single-Strand Break Mapping at Nucleotide Genome Level-AP (SSiNGLe-AP) technology as previously described [15]. The results revealed distinct genome-wide regional specificity in AP site distribution. We identified 18,686 gene-mapped AP sites in HCC827 cells, whereas genome-wide AP sites significantly increased to 70,708 in HCC827ER cells (Fig. 1a), indicating oxidative genomic damage accumulation in resistance. To examine the potential relationships between DNA damage and resistance, we conducted Gene Set Enrichment Analysis (GSEA) on the corresponding RNA-seq data and observed a marked upregulation of the base excision repair (BER) signaling pathway, alongside downregulation of double-strand break (DSB) repair, in resistant cells (Fig. 1b). OGG1 and APE1 play critical roles in BER, particularly in the repair of 8-oxoG- and AP-site damage, respectively. Given the aforementioned emerging role of oxidative damage in gene regulation, we examined OGG1 and APEX1 gene expression changes in EGFR-TKI sensitive and resistant cells and found that both were highly expressed in ER cells (Fig. 1c). Immunohistochemical (IHC) experiments were then performed to determine the levels of OGG1 and APE1 proteins in paired tissue samples from 13 patients with NSCLC before and after the development of ER. The IHC scores, based on the percentage of positively stained cells and staining intensity, were significantly higher for OGG1 and APE1 in post-resistant tumor tissues than in pre-resistant tissues (Fig. 1d). We further investigated the clinical relevance of OGG1/APEX1 expression in NSCLC by analyzing clinical samples from The Cancer Genome Atlas (TCGA) database. Our analysis revealed that both APEX1 and OGG1 were significantly upregulated in NSCLC tissues compared to normal controls (Fig. 1f). Notably, elevated APEX1 expression demonstrated a statistically significant association with poorer overall survival in NSCLC patients (*p* < 0.05) (Fig. 1e). Moreover, artificial overexpression (OE) of OGG1 and APE1 decreased the sensitivity of HCC827 and HCC4006 cells to erlotinib, whereas RNAi knockdown (KD) of OGG1 or APE1 decreased erlotinib resistance in HCC827ER and HCC4006ER cells (Fig. 1g). Thus, the collective data indicate a potential biological connection between oxidative base damage and BER pathway in the regulation of EGFR-TKI resistance.

### OGG1/APE1 driven transcriptional reprogramming established EMT- and stem-like plasticity in EGFR-TKI resistant NSCLC cells

Given that EGFR T790M mutations account for ~ 50% of first-generation EGFR-TKI resistance [16], we performed Sanger Sequencing (Sanger-seq) and Whole-Genome Sequencing (WGS-seq) and found no detectable T790M mutation of EGFR gene in the HCC827ER and HCC4006ER cell lines, indicating that acquired ER was driven by a non-T790M-based mechanism (Extended Data Fig. 1c). Therefore, we supposed resistance arises through alternative transcriptional programs that bypass EGFR dependency, driving TKI resistance.

**Fig. 2:**
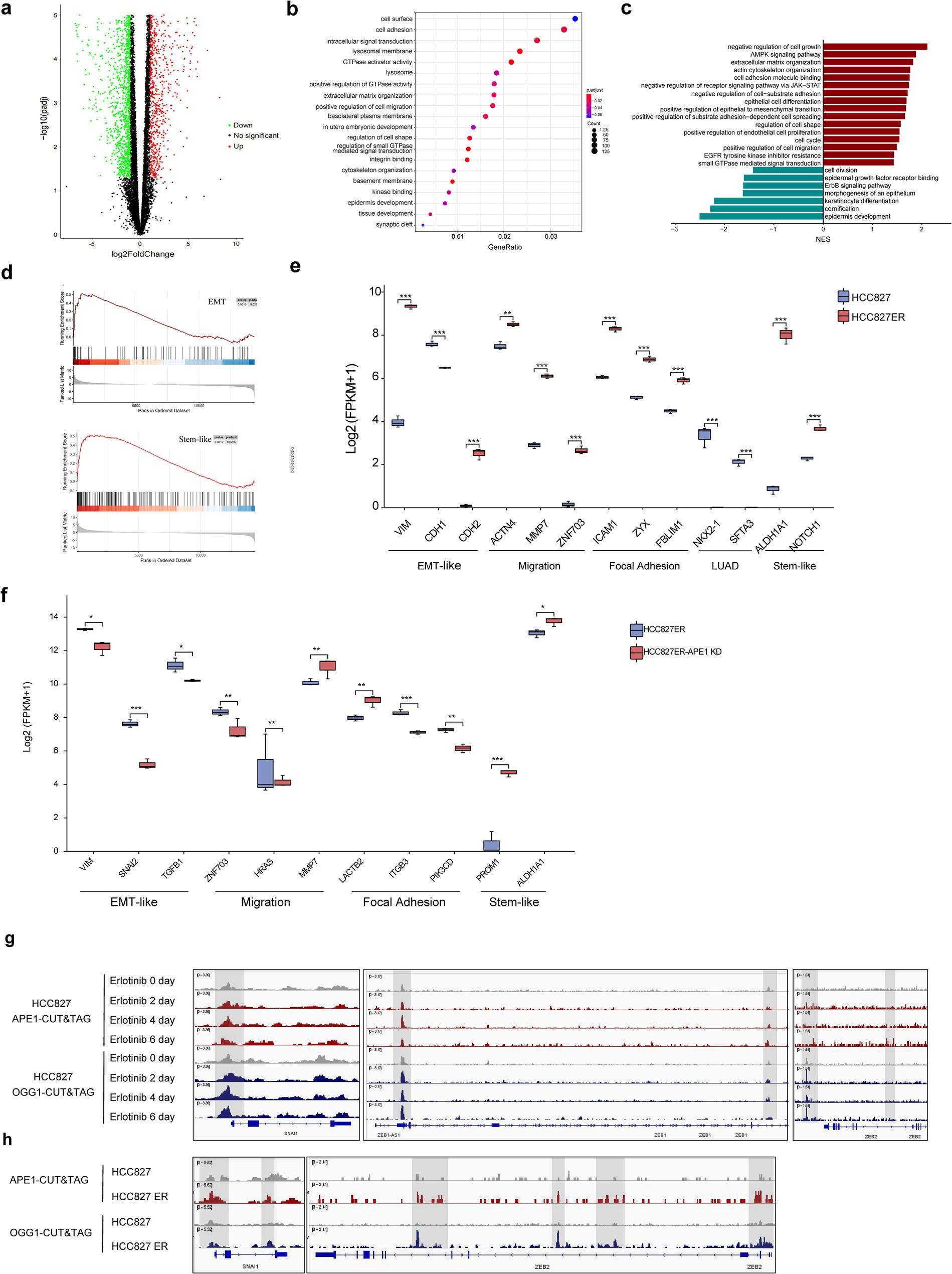
OGG1/APE1 driven transcriptional reprogramming established EMT- and stem-like plasticity in EGFR-TKI resistant NSCLC cells. **a**, Volcano plots representing differentially expressed genes (DEGs) in HCC827 and HCC827ER cells. Upregulated genes (red), downregulated genes (green). **b**, Gene Ontology (GO) Enrichment analysis reflected up and down regulated pathway terms associated with DEGs in HCC827 and HCC827ER cell lines. The dot color represented Normalized Enrichment Score (NES), and the dot size indicated the number of overlapping genes per pathway. **c**, GSEA pathway analysis highlighted cancer-related signaling pathways corresponding to lineage-specific signatures altered in HCC827ER cells compared to parental cells. NES：Normalized Enrichment Score. **d**, GSEA enrichment analysis revealed a strong correlation between DEGs and EMT- and stem-like signatures. **e**, Differential expression analysis of EMT, migration, focal adhesion, and cell-stem response genes in HCC827ER cells compared to parental cells. **f**, Differential expression analysis of EMT, migration, focal adhesion and cell stem response genes in HCC827ER APE1 KD cells compared to HCC827ER cells. **g**, Differential OGG1 and APE1 binding sites in SNAI1/ZEB1/ZEB2 region in HCC827 cells treated with 0.5 μm erlotinib for 0, 2, 4 and 6 days. **h**, Differential OGG1 and APE1 binding sites promoter regions in HCC827 versus HCC827ER cells

RNA sequencing (RNA-seq) analysis of EGFR-TKI-resistant HCC827ER cells unveiled transcriptomic reprogramming that underpins resistance to EGFR-TKIs (Fig. 2a). Differentially expressed genes (DEGs) were significantly enriched in adhesion/extracellular matrix (ECM) pathways, as indicated by gene ontology (GO) analysis (Fig. 2b). Furthermore, Kyoto Encyclopedia of Genes and Genomes (KEGG) pathway analysis demonstrated upregulation of ECM-receptor interaction, focal adhesion, and actin regulation pathways, concomitantly with downregulation of the EGFR/MAPK signaling cascade (Extended Data Fig. 1d). A well-characterized mechanism underlying non-genetically mediated adaptive resistance to EGFR-TKIs involves the rebound of phosphorylated ERK (p-ERK) signaling. Western blotting results were consistent with the sequencing data, demonstrating elevated p-ERK signaling in resistant cells. GSEA validated the suppression of the EGFR pathway and loss of Lung Adenocarcinoma (LUAD) markers, alongside upregulated multilineage plasticity (EMT-like, stem-like, JAK-STAT) that correlates with ER status (Fig. 2c, d). Marker validation confirmed plasticity signature elevation (Fig. 2e), collectively demonstrating resistance-associated transcriptional remodeling. Integrated RNA-seq/ATAC-seq analyses revealed that differentially accessible regions (DARs) showed > 50% concordance with transcriptional changes, leading to upregulation of membrane assembly, suppression of epithelial differentiation, and disruption of cell adhesion (Extended Data Fig. 1e-g).

Functional dissection via APE1/OGG1 knockdown in resistant cells (Extended Data Fig. 2a, c) reversed EMT/stem-like transcriptional networks—such as PI3K-Akt/MAPK signaling and cell adhesion—and restored differentiation programs (e.g., Notch signaling) (Fig. 2f and Extended Data Fig. 2b). OGG1 knockdown (OGG1-KD) further altered GPI-anchored protein regulation and CNS developmental pathways (Extended Data Fig. 2d, e). Notably, BHLH factors (E12, E47, TWIST1, TWIST2) and ZEB factors (ZEB1, ZEB2) have been identified as key regulators of EMT-like plasticity [17]. We found that time-dependent enrichment of APE1/OGG1 binding at genomic loci of EMT master regulators (SNAIL and ZEB factors) during erlotinib treatment (Fig. 2g), a pattern recapitulated in resistant versus parental cells (Fig. 2h). These chromatin occupancy dynamics establish direct BER-mediated transcriptional control over EMT plasticity through engagement with core transcriptional machinery, mechanistically linking oxidative damage repair to TKI resistance evolution.

### OGG1/APE1 mediate EMT- and stem-like plasticity to drive EGFR-TKI Resistance in NSCLC

**Fig. 3:**
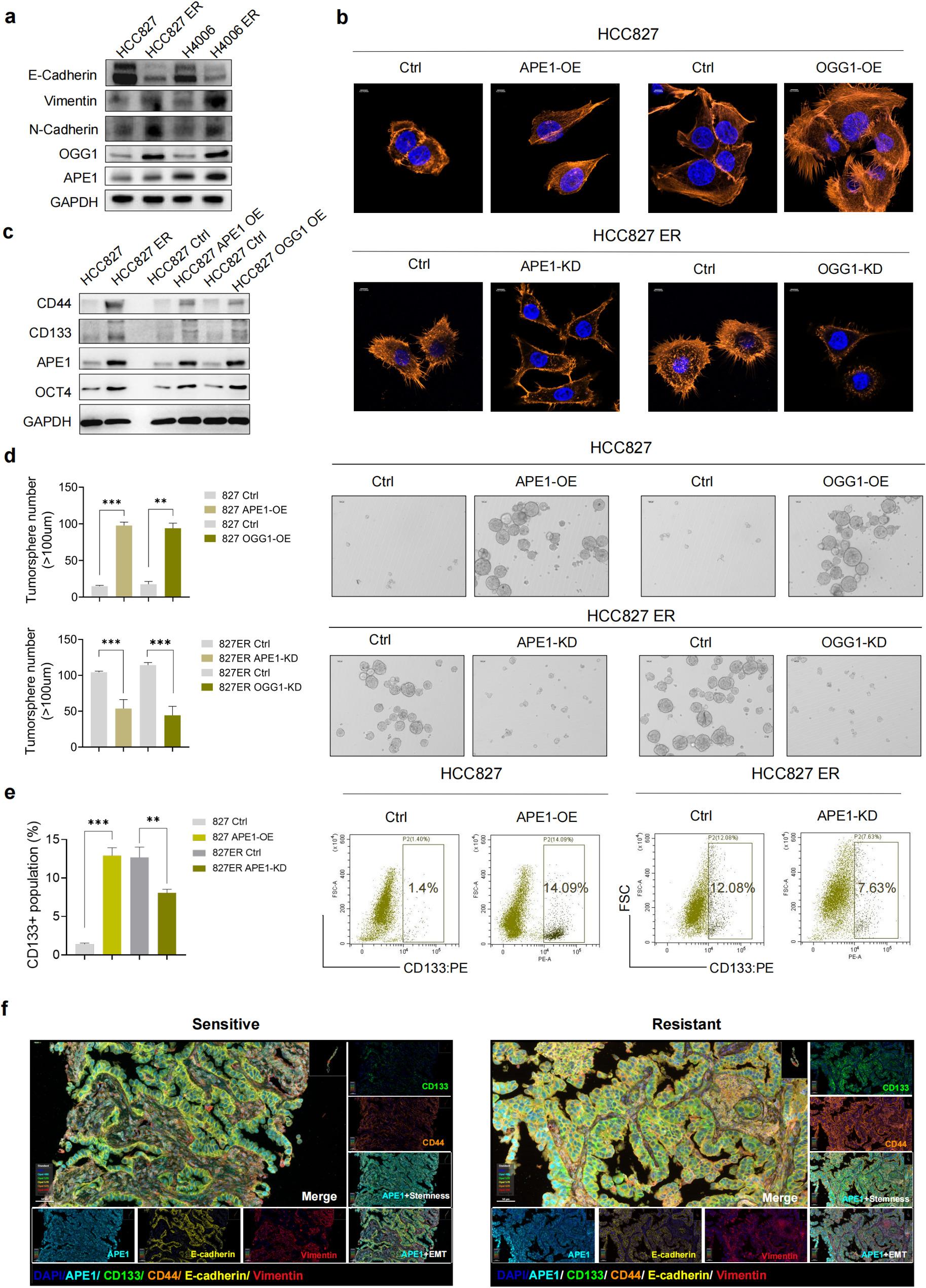
OGG1/APE1 mediate EMT- and stem-like plasticity to drive EGFR-TKI Resistance in NSCLC. **a**, Western blot analysis of EMT markers and APE1/OGG1 in two panels of sensitive and resistant cells. **b**, Phalloidin immunofluorescence showing enhanced actin stress fiber formation in HCC827 cells with OGG1 or APE1 overexpression (OE), and reduced stress fiber levels in HCC827ER cells with OGG1 or APE1 knockdown (KD). Control (Ctrl); Overexpressed (OE); Knockdown (KD). **c**, Western blot analysis of EMT and stemness markers in a panel of sensitive and resistant cells. **d**, Representative phase‑contrast images (right) and quantitative analysis (left) of tumorsphere formation in HCC827 (APE1/OGG1 OE) and HCC827ER (APE1/OGG1 KD) cells. Results are presented as mean ± SEM; n = 3; *P < 0.05, **P < 0.01, ***P < 0.001, independent t test. **e**, Surface expression of CD133 (cancer stem cell marker) in HCC827 (APE1/OGG1 OE) and HCC827ER (APE1/OGG1 KD) cells. Results are presented as mean ± SEM; n = 3; *P < 0.05, **P < 0.01, ***P < 0.001, independent t test. **f**, Representative illustration of human NSCLC tissue stained by multiplex IHC before and after resistance to first- and second-generation EGFR-TKI treatments. Scale bar, 50 µm. DAPI (blue), APE1 (cyan), E-cadherin (yellow), vimentin (red), CD44 (green), and CD133 (orange) staining

To functionally validate OGG1/APE1 in driving EMT- and stem-like phenotypes, we first performed invasion assays in parental/resistant cells with or without OGG1/APE1 modulation to assess EMT-associated migratory capacity. Remarkably, OGG1 or APE1 overexpression (OE) induced a more invasive phenotype in parental cells, whereas OGG1 or APE1 KD decreased invasive capacity in ER cells (Extended Data Fig. 3a). Western blot (WB) analysis of classic EMT markers, including E-Cadherin, N-Cadherin, and Vimentin, consistently supported the induction of EMT-like phenotypes in our model (Fig. 3a and Extended Data Fig. 4a). We further performed TRITC-phalloidin immunofluorescence staining to assess EMT-like phenotypic changes, and found that OE of OGG1 or APE1 in parental cells increased the abundance of actin stress fibers. Strikingly, silencing OGG1 or APE1 in HCC827ER or HCC4006ER cells markedly reduced actin stress fiber levels (Fig. 3b and Extended Data Fig. 4b). EMT and stem-like plasticity are mechanistically linked, sharing core genetic regulators [18, 19]. EMT enables tumor cells to adopt diverse intermediate states along the epithelial‑mesenchymal phenotypic axis, serving as a critical bridge between cancer stem cells (CSCs) and circulating tumor cells (CTCs) [20, 21]. Growing evidence establishes CSCs as pivotal drivers of tumor initiation, invasion, metastasis, recurrence, and therapeutic resistance. Induction of the stem-like phenotype was verified in acquired ER cells by WB. Remarkably, the expression of stemness‑associated marker genes [22] (CD44, CD133, and OCT4) as well as APE1 was markedly elevated in resistant cells compared with parental cells (Fig. 3c and Extended Data Fig. 4c). Additionally, OGG1 or APE1 OE in both HCC827 and HCC4006 cells robustly induced tumorsphere formation, whereas KD of OGG1 or APE1 in resistant cells abrogated this effect (Fig. 3d and Extended Data Fig. 4d). To further define the roles of OGG1 and APE1 in the induction of the stem-like phenotype, we assessed the expression of the classical tumor stemness marker CD133 on the cell surface by flow cytometry and observed that OGG1 and APE1 KD diminished the expression of CD133 in ER cells (Fig. 3e and Extended Data Fig. 4e). Multiplex immunohistochemistry (mIHC) on paired pre- and post-resistance tumor tissues (Fig. 3f) revealed APE1 enrichment in ER tumor regions adjacent to malignant cells, coinciding with elevated EMT (E-cadherin^−^/vimentin^+^) and stem-like (CD44^+^/CD133^+^) markers (Extended Data Fig. 3b). Collectively, these data establish OGG1 and APE1 as critical drivers of EMT and stem-like plasticity underlying EGFR-TKI resistance.

### Redistribution of genome-wide OGG1/APE1 binding sites and G4 structures in EMT- and stem-like related genes

**Fig. 4:**
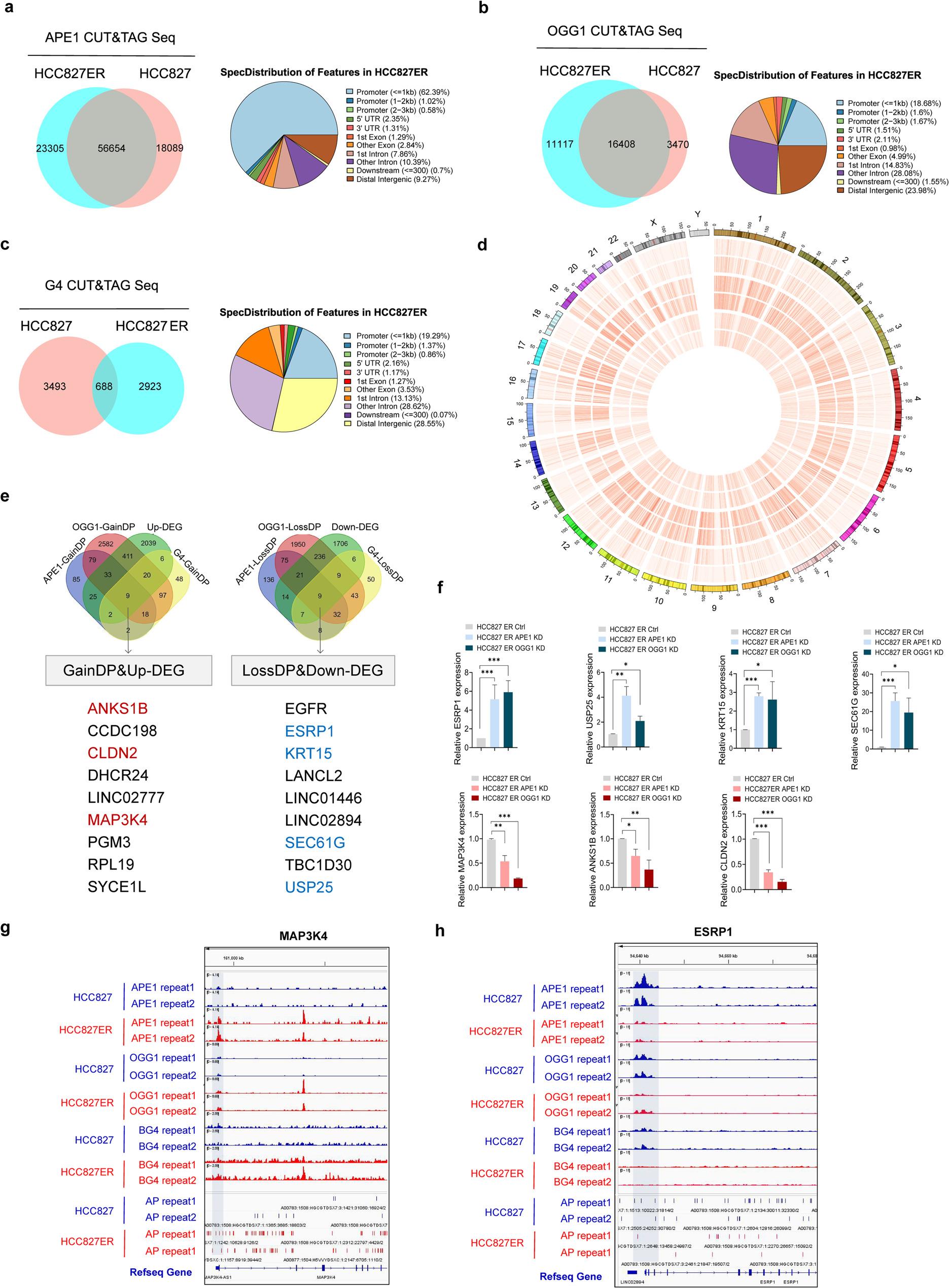
Redistribution of genome-wide OGG1/APE1 binding sites and G4 structures in EMT- and stem-like related genes. **a**, Venn diagram showing overlapping APE1 binding sites (APE1 peaks) between HCC827 and HCC827ER cells, with differential distribution across promoters, gene bodies, and intergenic regions. **b**, Venn diagram of overlapping OGG1 binding sites (OGG1 peaks) between HCC827 and HCC827ER cells, including genomic distribution analysis. **c**, Venn diagram of overlapping G-quadruplex (G4) structures (G4 peaks) between HCC827 and HCC827ER cells, with genomic distribution across promoter, gene body, and intergenic regions. **d**, Whole-genome circular plot displaying co-distribution of APE1, OGG1 binding sites, and G4 structures in HCC827 and HCC827ER cells. Genomic windows (100-kb) were used to calculate peak density. Tracks (from outer to inner): chromosomes; APE1 binding (HCC827); APE1 binding (HCC827ER); OGG1 binding (HCC827); OGG1 binding (HCC827ER); G4 structures (HCC827); G4 structures (HCC827ER). Color gradient (light red → dark red) indicates peak abundance (darker = higher density). **e**, Genomic distribution of 8-oxo-G/AP sites and G4 structures in ER resistant cells and their correlation with downstream gene expression. **f**, qRT-PCR analysis of EMT/stemness gene expression following OGG1/APE1 knockdown in resistant cells. Results are presented as mean ± SEM; n = 3; *P < 0.05, **P < 0.01, ***P < 0.001, independent t test. **g**, The shaded region highlights the overlap of OGG1, APE1, and BG4 binding in the MAP3K4 promoter region (HCC827 vs. HCC827ER), showing elevated AP site density in HCC827ER cells. **h**, The shaded region highlights the overlap of OGG1, APE1, and BG4 binding in the ESRP1 promoter region (HCC827 vs. HCC827ER), showing reduced AP site density in HCC827ER cells

Although OGG1/APE1 upregulation drives EGFR-TKI resistance by modulating EMT and stem-like plasticity, their genome-wide transcriptional mechanisms remain unknown. Therefore, to define how BER coordinates DNA damage response with transcriptional reprogramming for EMT/stemness, we performed Cleavage Under Targets and Tagmentation sequencing (CUT&Tag sequencing). Profiling of the chromatin binding patterns revealed differential APE1/OGG1 occupancy in HCC827 versus ER cells (Extended Data Fig. 5a). Specifically, HCC827ER cells exhibited a greater number of binding peaks for both OGG1 and APE1 compared to their HCC827 counterparts. Then, we mapped the OGG1 and APE1 binding peaks to annotated genomic features and found their differential distribution primarily in gene bodies (exons and introns) and promoter regions (defined as TSS ± 2 kb). Notably, binding sites within 1 kb of the transcription start site (TSS) were markedly enriched in HCC827ER cells (Fig. 4a, b).

We also determined the genome-wide distribution of G4 structures by using BG4-based Cleavage Under Targets and Tagmentation sequencing (BG4-CUT&Tag-seq). Genome-wide karyogram mapping revealed that G4 structure formation predominantly occurs in promoters, 5′ untranslated regions (5′ UTR), and gene bodies (Fig. 4c). Compared to parental cells, HCC827ER cells exhibited a higher signal enrichment intensity for OGG1/APE1 along the vertical axis at the TSS position. The distribution pattern of G4 within ± 2 kb of the TSS resembled the enrichment patterns of APE1 and OGG1 in HCC827ER cells (Extended Data Fig. 5b). By generating integrated distributions of APE1, OGG1, and G4 across the genomes of HCC827 and HCC827ER cells, we uncovered ‘hotspots’ for enrichment in resistant cells. The results indicated that the binding of major DNA repair proteins and the positions of G4 structures are non-random and occur in genome-specific regions (Fig. 4d).

To identify the downstream genes affected by the above features, we integrated the G4, OGG1, and APE1 mapping data—including OGG1-CUT&Tag, APE1-CUT&Tag, and BG4-CUT&Tag in parental and resistant HCC827 cells—with differential gene expression profiles from RNA-seq. Our analysis yielded candidate gene clusters implicated in oxidative DNA damage-related epigenetic mechanisms. Specifically, Group 1 included genes that were upregulated with increased OGG1, APE1, and G4 binding peaks (log2 fold change ≥ 2, *p < 0.05): ANKS1B, CCDC198, CLDN2, DHCR24, LINC02777, MAP3K4, PGM3, RPL19, and SYCE1L. Group 2 included downregulated genes with reduced OGG1, APE1, and G4 binding (log2 fold change ≥ 2, *p < 0.05): EGFR, ESRP1, KRT15, LANCL2, LINC01446, LINC02894, SEC61G, TBC1D30, and USP25 (Fig. 4e). Metascape analysis showed that this selected gene cluster was significantly enriched in the regulation of cellular stress response (Extended Data Fig. 5c). Interestingly, 25% of Group 1 (upregulated) and 30% of Group 2 (downregulated) genes have been reported to be involved in EMT- and stem-like plasticity acquisition, including MAP3K4 [22], ANKS1B [23], CLDN2 [24] (upregulated), ESRP1 [25], USP25 [26], SEC61G [27] and KRT15 [28, 29] (downregulated) (Fig. 4e). Functional validation showed that OGG1/APE1 KD in ER cells downregulated Group 1 genes (MAP3K4, ANKS1B, CLDN2) and upregulated Group 2 genes (ESRP1, USP25, SEC61G, KRT15) (Fig. 4f and Extended Data Fig. 5d). Subsequently, IHC analysis of paired samples from 13 NSCLC patients showed significantly higher MAP3K4 and reduced ESRP1 expression in post-ER tumors compared to pre-ER tissues (Extended Data Fig. 5e). In ER cells with upregulated MAP3K4, G4 structure formation and OGG1/APE1 binding were observed in the ER-shaded region, where more AP sites were detected (Fig. 4g). While in ER cells with downregulated ESRP1, the opposite phenomenon was observed (Fig. 4h). Thus, the collective data indicate a possible biological connection between oxidative base damage, the binding of OGG1 and APE1, and the presence of G4 structures in the regulation of EGFR-TKI resistance. 

### APE1 exhibits stronger affinity towards promoter G4 regions than non-G4 regions in EMT- and stem-like related genes

**Fig. 5:**
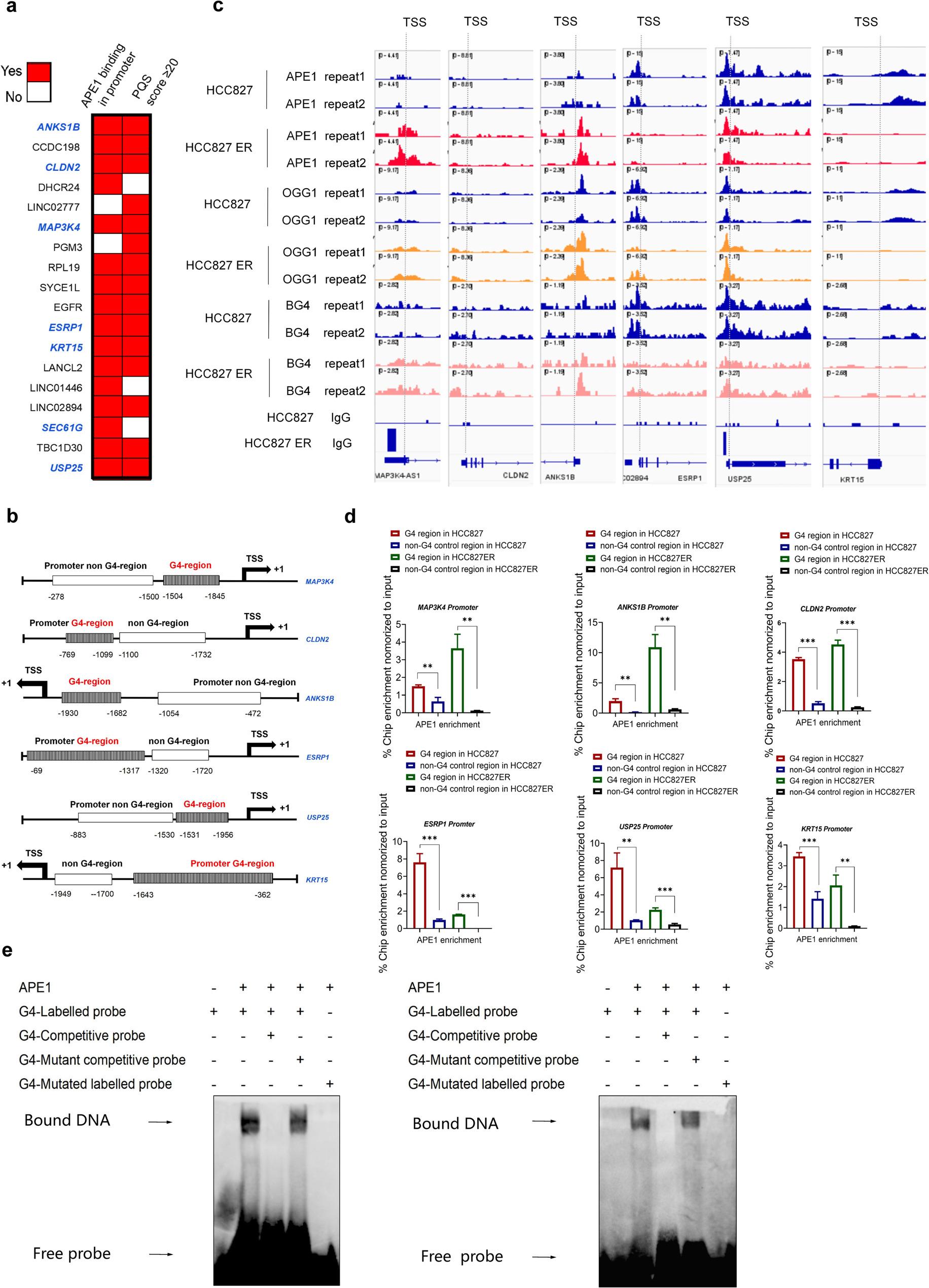
APE1 exhibits stronger affinity towards promoter G4 regions than non-G4 regions in EMT- and stem-like related genes. **a**, Occurrence of APE1 binding sites in G4-containing promoters of DEGs (PQS score ≥20 considered significant). **b**, Schematic highlighting G4-forming regions (shaded) and non-G4 control regions (white box) in promoters of EMT/stemness genes (MAP3K4, CLDN2, ANKS1B, ESRP1, USP25, KRT15). G4 regions were identified via QGRS Mapper (PQS score ≥20). **c**, Representative genome browser views of EMT and stemness-related gene promoter regions (MAP3K4, CLDN2, ANKS1B, ESRP1, USP25, and KRT15) showing OGG1/APE1 binding and G4 profiles in HCC827 and HCC827ER cells. **d**, ChIP-qPCR validation of APE1 enrichment at G4 vs. non-G4 regions in EMT and stemness-related genes (MAP3K4, CLDN2, ANKS1B, ESRP1, USP25, and KRT15) in HCC827 and HCC827ER cells. Primers were utilized to amplify the G4 region and a non-G4 control region. The P-values were determined by an unpaired Student’s t-test (****P < 0.0001, ***P < 0.001, **P < 0.01, *P < 0.05). Error bars denote ± SEM. Three independent experiments were performed in triplicates. **e** and **f**, EMSA confirming APE1 binding to G4-forming oligonucleotides (derived from ANKS1B and ESRP1 promoters) and non-G4 controls. Biotinylated G4 oligonucleotides and mutant control sequences were constructed

To identify potential functional links between G4 structures, BER protein binding and activity and transcriptional robustness, Integrative Genomics Viewer (IGV) was used to determine the occupancy of APE1 in the gene cluster promoter regions, particularly in EMT and stemness-related genes. QGRS Mapper (Quadruplex-forming G-rich Sequence Mapper) analysis revealed PQS scores ≥ 20 (significant threshold) in promoter regions of these genes, except for SEC61G (Fig. 5a). Promoter regions upstream of the TSS were classified as G4-forming or non-G4-forming (Fig. 5b). IGV analysis revealed greater co-occupancy of APE1, OGG1, and G4 in EMT- and stem-like plasticity-related genes (MAP3K4, CLDN2, ANKS1B) versus reduced co-occupancy in ESRP1, USP25, KRT15 in ER cells. Critically, APE1 binding was stronger in G4-forming promoter regions than in non-G4-forming regions in both parental and ER cells (Fig. 5c).

Using Chromatin Immunoprecipitation Quantitative Polymerase Chain Reaction (ChIP-qPCR) in parental and resistant cells, our analysis revealed significant enrichment of APE1 in the G4 regions of the EMT- and stem-like related DEGs promoters, but no enrichment in control non-G4 regions (Fig. 5d). To directly test the binding of APE1 with G4 structures, we next employed electrophoretic mobility shift assay (EMSA) with purified APE1 and biotin-labeled G4-forming oligonucleotide substrates, designed to mimic potential G4 forming sequences in the promoters of ANKSIB and ESRP1, or non-G4-forming controls (mutant controls). We found that APE1 showed stronger binding affinity for G4 substrates than non-G4 controls (Fig. 5e-f).

### APE1 modulates transcription of EMT- and stem-like related genes by stabilizing G4 structures in cells

**Fig. 6:**
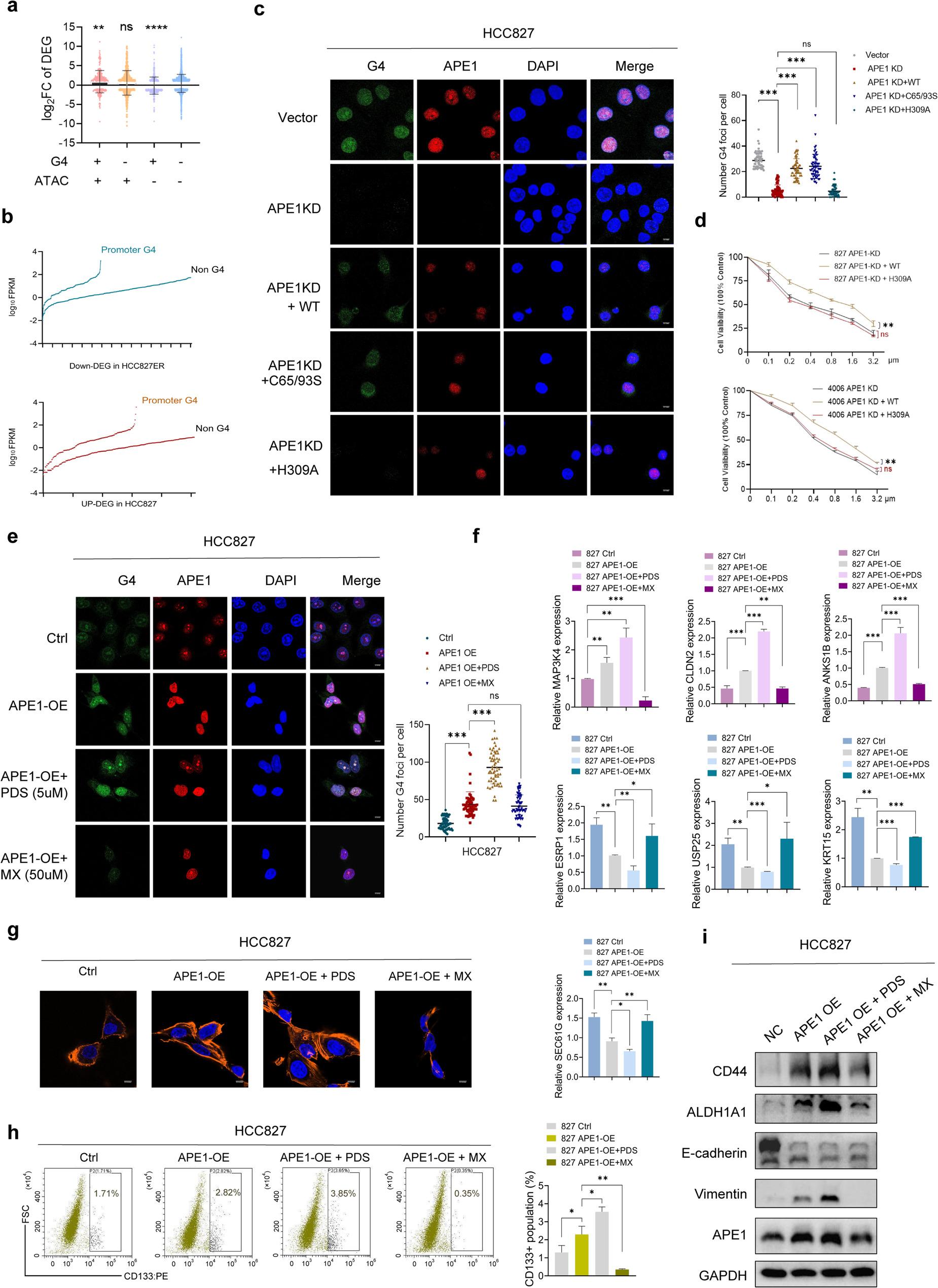
APE1 modulates transcription of EMT- and stem-like related genes by stablizing G4 structures in cells. **a**, Differential gene expression in HCC827 vs. HCC827ER cells categorized by G4 and ATAC signal presence, analyzed relative to ATAC-/G4- baseline. The presence (+) or absence (−) of G4s or ATAC signals are reported. Gene expression distribution of the differentially expressed genes in HCC827 vs. HCC827ER cells was evaluated by two-sided t test in comparison to the G4:ATAC -/- condition (CI 95%, ***p value < 0.001). **b**, Expression distribution of DEGs grouped in HCC827 vs. HCC827ER cells according to the presence of G4 signals (BG4-CUT&TAG Seq) in their gene region. Gene expression is reported as log10FPKM. **c**, APE1 KD HCC827 cells were transfected with APE1 WT plasmid, redox-defective C65/93S, or repair-defective H309A plasmid for 48 hours. Cells were then immunostained with α-1H6 and APE1, counterstained with DAPI, and visualized by confocal microscopy (Magnification: 63×; Scale bars: 0.3 inches). **d**, Erlotinib dose-response curves for APE1 KD HCC827 cells transfected with APE1 WT plasmid, redox-defective C65/93S, or repair-defective H309A plasmid. n = 3 experimental replicates, mean ± SEM *P < 0.05, **P < 0.01, ***P < 0.001. **e**, Representative fields of view showing G4 foci formation detected by confocal microscopy in control, APE1 OE, APE1 OE + PDS (5 µM, 2 hours), and APE1 OE + MX (50 µM, 2 hours) treated HCC827 cells (Magnification: 63×; Scale bars: 0.3 inches). **f**, qRT-PCR assays were performed in control, APE1 OE, APE1 OE + PDS (5 µM, 2 hours), and APE1 OE + MX (50 µM, 2 hours) treated HCC827 cells, and relative gene expression (normalized to GAPDH) of EMT and stemness-related genes (MAP3K4, CLDN2, ANKS1B, ESRP1, USP25, and KRT15) was calculated. The P-values were determined by an unpaired Student’s t test (****P < 0.0001, ***P < 0.001, **P < 0.01, *P < 0.05). Error bars denote ± SEM. Three independent experiments were performed in triplicates. **g**, Actin stress fiber quantification (phalloidin staining, 63×; scale bar = 0.3 mm) in control, APE1 OE, APE1 OE + PDS (5 µM, 2 h), and APE1 OE + MX (50 µM, 2 h) cells. **h**, The expression of the classical tumor stemness marker CD133 on the cell surface in control, APE1 OE, APE1 OE + PDS (5 µM, 2 hours), and APE1 OE + MX (50 µM, 2 hours) treated HCC827 cells. **i**, Protein expression of EMT and stemness markers was analyzed by western blot in control, APE1 OE, APE1 OE + PDS (5 µM, 2 hours), and APE1 OE + MX (50 µM, 2 hours) treated HCC827 cells

Nonrandom G4 distribution across the human genome has been previously reported [30, 31]. In line with this, BG4 anti‑G4 antibody (BG4) peaks were strikingly prevalent in ± 3 kb of the TSSs regions in the whole genome of HCC827 and HCC827ER cells (Extended Data Fig. 6a). To assess the extent of different transcription levels between sensitive and resistant cells, we quantified the transcriptional levels of DEGs within permissive and silenced chromatin regions relative to the presence of folded G4s and open chromatin signals. This revealed that actively transcribed DEGs were predominantly localized to open chromatin, with G4-harboring DEGs exhibiting significantly greater transcriptional divergence (Fig. 6a). Specifically, DEGs within the open chromatin regions carrying G4 structures displayed enhanced transcriptional activity (Extended Data Fig. 6b). Moreover, genes harboring BG4 peaks consistently exhibited higher levels of transcriptional activity regardless of chromatin status (Fig. 6b). IGV visualization revealed distinct distribution patterns of G4 structures were uncovered for EMT- and stem-like phenotype-associated DEGs. Upregulated EMT- and stem-like phenotype-associated DEGs in ER cells exhibited a higher frequency of G4 formation in open chromatin regions, whereas downregulated DEGs displayed the opposite trend (Extended Data Fig. 6c). To further support the positive connection between active transcription and G4s, we measured G4 foci by immunofluorescence in parental HCC827 and HCC4006 cells treated with either the histone deacetylase (HDAC) inhibitor entinostat (which stabilizes transcriptionally active chromatin) or the RNA polymerase II inhibitor actinomycin D (which blocks transcriptional elongation). Compared with untreated controls, G4 foci exhibited a dose-dependent increase in entinostat-treated HCC827 and HCC4006 cells (Extended Data Fig. 6d). Additionally, treatment with actinomycin D reduced the apparent number of folded G4 structures, reinforcing the link between active transcription and G4 stabilization (Extended Data Fig. 6e). To establish causality between APE1, G4 structure formation, and transcriptional regulation, we performed CUT&Tag-seq for Pol II in APE1 KD cells. APE1 KD significantly reduced both BG4 (G4 structure) and Pol II enrichment at the promoter regions of upregulated EMT and stemness-related genes, with the inverse pattern observed for downregulated genes (Extended Data Fig. 6f). Consistently, APE1 KD decreased Pol II CTD Ser5/Ser2 phosphorylation, indicating impaired transcriptional activation (Extended Data Fig. 6g). These findings establish direct evidence linking APE1-mediated G4 structure stability to transcriptional upregulation of EMT- and stemness-related genes.

Next, we detected G4 structures predominantly in the nuclei of both drug-sensitive and -resistant cells and observed frequent colocalization of G4 structures and APE1 with a G4 DNA-specific antibody (α-1H6) through laser confocal fluorescence microscopy (LCFM). Strikingly, APE1 KD via shRNA in ER cells almost completely abolished G4 detection, indicating that APE1 is essential for the formation or stability of G4 structures (Extended Data Fig. 7a). A similar phenotype was observed upon APE1 KD in parental cells (Fig. 6c and Extended Data Fig. 8b). Conversely, treatment with the G4-stabilizer Pyridostatin (PDS) robustly increased G4 signals and their co-localization with APE1, which was blocked by APE1 knockdown (Extended Data Fig. 7a). APE1 is a multifunctional protein known for its AP site DNA repair activity and its transcriptional regulatory function [32]. To ascertain the significance of APE1’s AP site repair/binding activity, we treated HCC827ER cells with methoxyamine (MX), which competitively inhibits the binding of APE1 to AP sites [33]. Pretreatment of cells with MX significantly reduced G4 structure foci (Extended Data Fig. 7b). Conversely, treatment with E3330, which is widely accepted as an APE1 redox antagonist [34], did not alter the fluorescence intensity of G4 (Extended Data Fig. 7c), implying that the APE1 AP site repair activity and not its redox function influences G4 formation in cells. Supporting this conclusion, reintroduction of wild-type (WT) APEX1 into APE1-KD cells effectively rescued G4 formation, whereas transfection with the empty vector did not. Although a similar effect on G4 detection was observed in cells transfected with an APE1 redox mutant (C65S/C93S) plasmid [35], ectopic expression of a nuclease-deficient H309 (H309A [36]) plasmid in APE1-KD cells failed to restore G4 foci (Fig. 6c and Extended Data Fig. 8b). The H309 residue in the active site pocket of APEX1 has been identified as a key residue involved in hydrogen bonding with AP sites and in coordinating catalysis [36]. And the expression levels of WT and mutant APE1 proteins were confirmed by WB (Extended Data Fig. 8a). Taken together, these results indicate that the DNA repair activity of APE1 and its binding to AP sites modulate G4 formation.

Notably, restoring WT APEX1, but not the nuclease-deficient H309 mutant (H309A), in APE1-KD HCC827 and HCC4006 cells significantly enhanced their sensitivity to erlotinib (Fig. 6d). Transfection of the APEX1 OE plasmid into parental HCC827 and HCC4006 cells increased the formation of G4 foci, whereas pre-treatment with MX diminished the APE1-mediated effect (Fig. 6e and Extended Data Fig. 8c). Importantly, focusing specifically on EMT- and stem-like related DEGs, we observed that overexpression of either OGG1 or APE1 in parental cells significantly increased the mRNA expression of MAP3K4, ANKS1B, and CLDN2 and decreased the expression of ESRP1, USP25, KRT15, and SEC61G (Fig. 6f and Extended Data Fig. 9a). In parallel, we observed that treatment with PDS significantly increased the expression of MAP3K4, ANKS1B, and CLDN2 in control or APE1 OE parental cells, whereas the expression of ESRP1, USP25, KRT15, and SEC61G was downregulated. Conversely, treatment of control or APE1 OE cells with MX exerted the opposite effect (Fig. 6f and Extended Data Fig. 9a). Additionally, PDS treatment significantly increased the number of actin stress fibers, as shown by F-actin staining, and elevated expression of the cell surface stemness marker CD133, whereas MX treatment effectively suppressed these phenotypes (Fig. 6g, h and Extended Data Fig. 9b, c). Lastly, western blot analysis showed that APE1 OE in HCC827 cells upregulated the protein levels of EMT- and stem-like markers (CD44, Vimentin and ALDH1A1) and that PDS (5 µM) or MX (50 µM) treatment could promote or inhibit this effect, respectively (Fig. 6i and Extended Data Fig. 9d). However, PDS treatment also promoted tumorsphere formation capacity in these drug-sensitive cell lines, although the inductive effect was weaker than that induced by APE1 OE (Extended Data Fig. 9e). Similarly, PDS treatment only mildly upregulated EMT markers in parental NSCLC cells (Extended Data Fig. 9f, g), Overall, our results demonstrate that APE1 promotes the formation of G4 structures at the promoters of EMT- and stem-like related DEGs, thereby driving their expression and associated phenotypes.

### G4 structures are found within hypomethylated CGIs in EMT- and stem-like related genes

Chromatin structure and gene expression are regulated by the complex interplay between epigenetic marks (such as DNA methylation, histone modifications, and nucleosome positioning) and the binding of core transcription factors to regulatory DNA [31]. Since APE1 can tolerate TKI stress by stabilizing genomic G4 structures and activating EMT- and stem-like gene programs, we questioned whether other epigenetic marks cooperate with G4 structures to maintain cell-specific transcriptional programs, thereby shaping the drug-resistant transcriptome. G4 structures have been reported to be recognized and bound by DNA Methyltransferases (DNMTs) [37], implying a close functional link between G4s and DNA methylation.

**Fig. 7:**
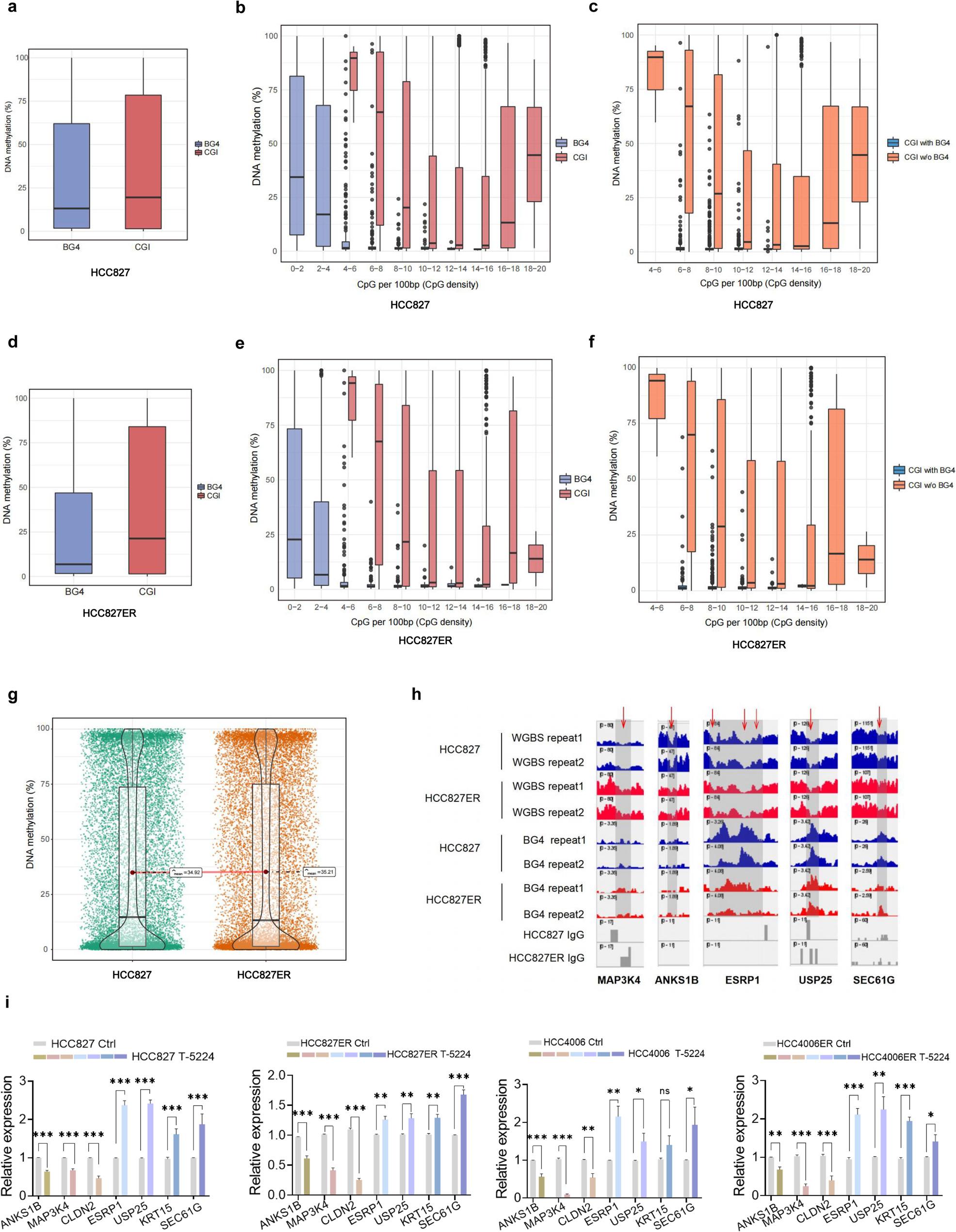
G4 structures are found within hypomethylated CGIs in EMT- and stem-like related genes. **a**, Box and whisker plots showing the average methylation for BG4 peaks and CGIs in HCC827 cells. **b**, Methylation levels at BG4 peaks and CGIs across CpG density bins in HCC827 cells. **c**, Methylation levels at CGIs with or without a BG4 peak at different CpG densities in HCC827 cells. **d**, Methylation levels at BG4 peaks and CGIs in HCC827ER cells. **e**, Methylation levels at BG4 peaks and CGIs across CpG density bins in HCC827 cells. **f**, Methylation levels at CGIs with or without a BG4 peak at different CpG densities in HCC827ER cells. **g**, Comparison of CGI methylation levels between HCC827 and HCC827ER cells. **h**, An IGV screenshot illustrating the co-incidence of BG4 peaks with hypomethylated promoter CGIs for EMT and stemness-related genes (MAP3K4, CLDN2, ANKS1B, ESRP1, USP25, and KRT15) in HCC827 and HCC827ER cells. Shown are normalized signals. **i**, qRT-PCR analysis of EMT and stemness-related genes expression following T-5224 (5 µM, 24 h) treatment in parental (HCC827) and resistant (HCC827ER) cells. Results are presented as mean ± SEM; n = 3; *P < 0.05, **P < 0.01, ***P < 0.001, independent t test

We further validated whether the collaboration between G4s and DNA methylation simultaneously affects the expression of genes related to EMT and stemness in TKI resistance. Towards that end, we performed single-base resolution whole-genome bisulfite sequencing (WGBS) to globally profile DNA methylation in HCC827 and HCC827ER cells. In mammalian genomes, CpG islands (CGIs) are short, GC‑rich genomic regions with a high density of CpG dinucleotides, generally characterized by low methylation levels and active transcription [38, 39]. We then examined the overlap between BG4 CUT&Tag peaks (representing G4 structures) and annotated CGIs (UCSC hg38). The comparative analysis revealed that approximately one-third of BG4 peaks (32.1%, 1011/3145 in HCC827 cells; 32.8%, 885/2698 in HCC827ER cells) overlapped with CGIs, with the majority of these BG4 peaks containing at least one CGI (Extended Data Fig. 10a-b). The majority of CGI regions spanned 200–1000 bp, whereas BG4 peaks ranged from 100 to 400 bp (Extended Data Fig. 10c). Consistent with previous studies showing that CpG density and high GC content were required for establishing a low-methylation state within CGIs [40, 41], we identified that CGIs exhibited higher GC enrichment levels in BG4 peaks (Extended Data Fig. 10d). In addition, BG4 peaks exhibited significantly lower methylation levels than the genome-wide average in both HCC827 (Fig. 7a) and HCC827ER (Fig. 7d) cells. Notably, when comparing the methylation levels in CGIs and BG4 peaks at different CpG densities, we observed a consistent lack of methylation in BG4 peaks over a wide range of CpG densities in both HCC827 (Fig. 7b) and HCC827ER (Fig. 7e) cells. By analyzing the correlation between methylation levels and G4 occupancy, we found that CGIs overlapping G4 peaks were almost completely unmethylated—in contrast to CGIs lacking BG4 peaks—demonstrating that G4-associated CGIs are inherently hypomethylated and functionally relevant (Fig. 7c and f). The distribution of the overall methylation site quantity and level in the HCC827 and HCC827ER genomes is illustrated in Fig. 7g. By examining the EMT- and stem-like related DEGs, we found that the hypomethylation status of CGIs in these genes was mainly located at sites harboring G4 peaks (Fig. 7h and Extended Data Fig. 10e), indicating that the physical presence of G4s was an important feature associated with hypomethylation status.

Next, we predicted the presence of putative transcription factor binding sites (TFBSs) within the differential G4s using Homer software and observed that the most relevant TFBS were enriched in AP-1-family (such as FOS and JUN) motif binding sites (AP-1, 26.07% in gained BG4 peaks vs. 7.02% in background sequences, *p* = 1e − 16 and 10.22% in loss BG4 peaks vs. 5.99% in background sequences, *p* = 1e − 2) (Extended Data Fig. 10f). Given that AP-1 binding is regulated by CGI methylation [42, 43]and that G4 structures can modulate chromatin accessibility [44], we hypothesized that folded G4 structures in chromatin may promote AP-1 recruitment. To functionally validate the involvement of AP-1 in G4-associated gene regulation, we examined the effect of AP-1 inhibition on the expression of G4-linked EMT and stemness genes in parental and erlotinib-resistant cells. Treatment with T-5224, a specific AP-1 DNA-binding inhibitor, significantly reduced the transcript levels of these genes in both drug-sensitive and -resistant cell lines (Fig. 7i). Notably, genes transcriptionally upregulated in resistant cells showed reduction in expression following AP-1 inhibition. These findings provide functional evidence that AP-1 activity contributes to the transcriptional activation of G4-associated genes, consistent with the enrichment of AP-1 motifs near differential G4 peaks.

### Combination with BER inhibitors sensitizes ER cells to EGFR-TKI and suppresses xenograft tumor growth in vivo

**Fig. 8:**
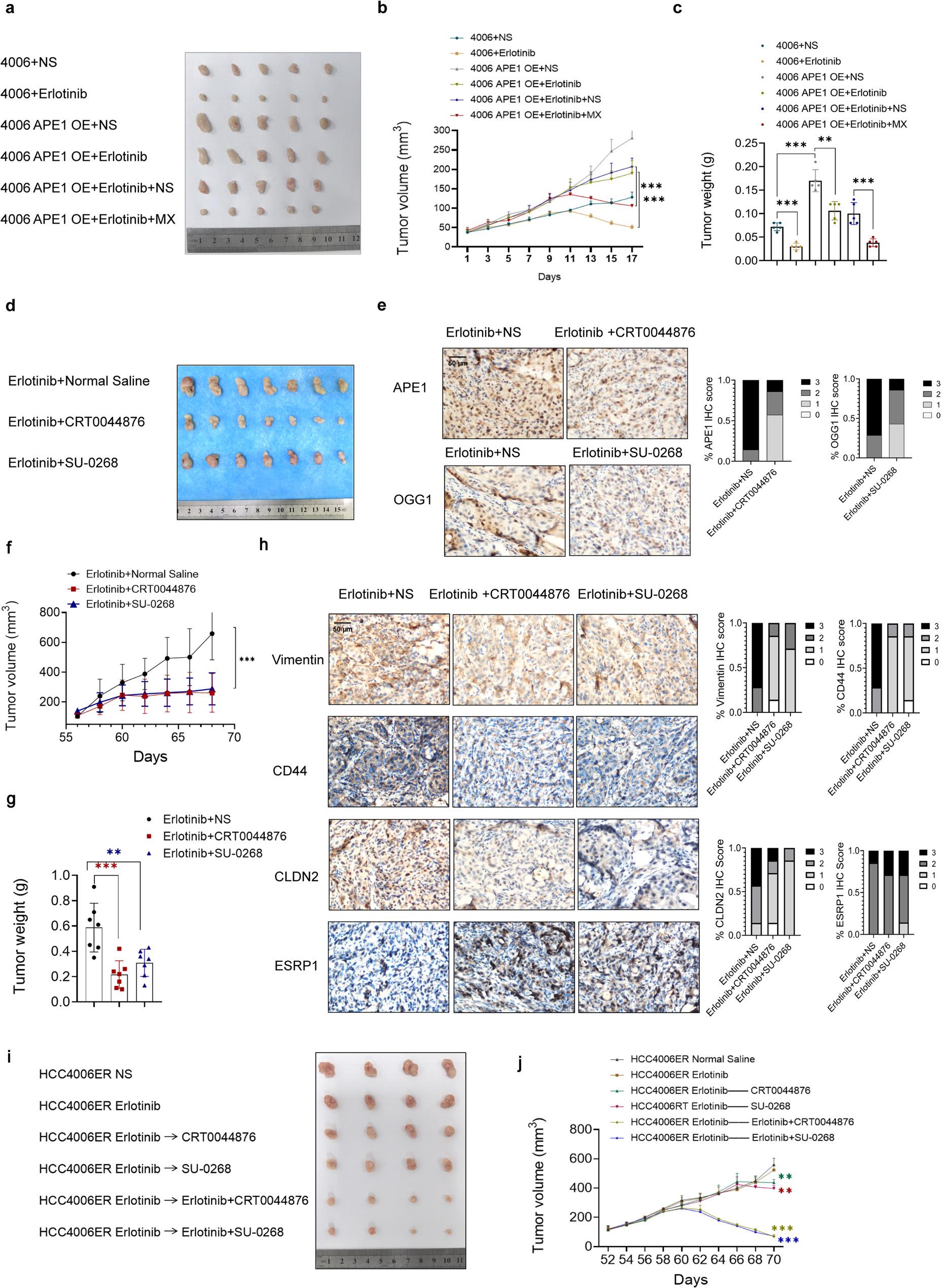
Combined with BER inhibitors sensitizes ER cells to EGFR-TKI and suppresses xenograft tumor growth in vivo. **a**, Resected tumors after completion of treatment. Thirty 4-week-old male BALB/ude mice were randomized into two groups: HCC4006 control (n=10) and HCC4006 APE1 OE (n=20). Mice received subcutaneous injections of 5×10⁶ cells (100 μL) in the right flank. At tumor volumes of ~100 mm³, the control group was divided into NS and erlotinib (10 mg/kg) subgroups, while the APE1 OE group was allocated to four treatment arms: NS, Erlotinib, Erlotinib + NS, and Erlotinib + MX (10 mg/kg, i.p., every 2 days). **b**, Tumor volume was measured on indicated days, and the tumor growth curve was plotted. **c**, Tumor weight of resected tumors after completion of treatment. **d**, Resected tumors after completion of treatment. 3×10⁶ HCC4006ER cells were subcutaneously injected into 4-week-old mice (20 g average weight). After 4 weeks, tumor-bearing mice were randomized into three groups (n=7/group): (1) Erlotinib (10 mg/kg) + NS; (2) Erlotinib + CRT0044876 (APE1 endonuclease inhibitor, 5 mg/kg, i.p., 3×/week); (3) Erlotinib + SU-0268 (OGG1 inhibitor, 10 mg/kg, i.p., 3×/week). Tumor growth was monitored biweekly until euthanasia at 10 weeks (pentobarbital, 50 mg/kg, i.p.), with tumor collection (<2,000 mm³). CRT0044876 and SU-0268 were from MCE (China); MX from Sigma-Aldrich (USA). **e**, Representative IHC images of OGG1 and APE1 expression in resected tumors (Scale bar, 50 µm). **f**, Tumor volume was measured on indicated days, and the tumor growth curve was plotted. **g**, Tumor weight of resected tumors after completion of treatment. **h**, Representative IHC images of Vimentin, CD44, CLDN2, and ESRP1 expression in resected tumors (Scale bar, 50 µm). **i**, Resected tumors after completion of treatment. 3×10⁶ HCC4006ER cells were subcutaneously injected into 4-week-old mice (20 g average weight). After 4 weeks, tumor-bearing mice were randomized into six groups (n=4/group): (1) Control: NS; (2) Erlotinib (10 mg/kg); (3) Erlotinib (10 mg/kg, continued treatment for 6 days) followed by CRT0044876 (APE1 endonuclease inhibitor, 5 mg/kg, i.p., 3×/week); (4) Erlotinib (10 mg/kg, continued treatment for 6 days) followed by SU-0268 (OGG1 inhibitor, 10 mg/kg, i.p., 3×/week); (5) Erlotinib (10 mg/kg, continued treatment for 6 days) followed by CRT0044876 (APE1 endonuclease inhibitor, 5 mg/kg, i.p., 3×/week) combined with erlotinib (10 mg/kg); (6) Erlotinib (10 mg/kg, continued treatment for 6 days) followed by SU-0268 (OGG1 inhibitor, 10 mg/kg, i.p., 3×/week) combined with erlotinib (10 mg/kg). Endpoint: 10 weeks or <2,000 mm³. **j**, Tumor growth curves for panel i model

To investigate the role of APE1 in NSCLC tumor growth and evaluate tumor sensitivity to erlotinib in vivo, we established a xenograft model using HCC4006 control and APE1 OE cells. Erlotinib significantly inhibited tumor growth in control xenografts but exhibited minimal efficacy in APE1 OE xenografts. Notably, co-treatment with MX significantly enhanced tumor suppression, suggesting that APE1-G4-mediated gene expression changes can modulate TKI sensitivity in vivo (Fig. 8a-c). Immunohistochemical analysis confirmed elevated APE1 expression in the mouse model and revealed corresponding alterations in EMT and stemness markers, including increased vimentin and CD44 expression, elevated CLDN2 levels, and reduced ESRP1 expression, consistent with our screening results (Extended Data Fig. 11a). In xenograft experiments with parental cell lines to evaluate the antitumor efficacy of PDS alone and in combination with erlotinib, PDS monotherapy failed to effectively suppress tumor growth in this model, whereas the combination significantly inhibited tumor growth (Extended Data Fig. 11b, c). In a separate therapeutic evaluation, we treated palpable tumors in xenograft models with erlotinib combined with either the APE1 inhibitor CRT0044876 (5 mg/kg) or the OGG1 inhibitor SU-0268 (10 mg/kg) (Fig. 8d). Both combination therapies demonstrated superior therapeutic effects compared to erlotinib monotherapy in HCC4006ER-bearing mice. The significant reduction in tumor volume following combination treatment suggests that inhibition of either BER protein sensitizes HCC4006ER cells to TKI treatment and suppresses tumor growth in vivo (Fig. 8f, g). Quantitative immunohistochemical assessment validated effective APE1 and OGG1 knockdown in our mouse models (Fig. 8e). Consistent with our screening results, we observed corresponding changes in EMT and stemness markers, including increased vimentin expression, decreased CD44 levels, reduced CLDN2, and elevated ESRP1 (Fig. 8h). Using a subcutaneous xenograft model with endogenous APE1-KD HCC4006ER cells, we observed that erlotinib treatment significantly suppressed tumor growth in APE1-KD cells compared to controls (Extended Data Fig. 11d, e). In established HCC4006ER xenografts, late monotherapy with APE1 or OGG1 inhibitors induced mild tumor shrinkage, demonstrating that BER pathway inhibition remains effective even after full resistance establishment. These data indicate that APE1 and OGG1 sustain resistance via EMT/stemness pathways, not merely driving initial resistance emergence (Fig. 8i, j). This single‑agent effect was far weaker than the robust tumor regression achieved by combining BER inhibitors with erlotinib, which acts through a synergistic double‑hit mechanism: erlotinib suppresses residual EGFR signaling, while BER inhibition enhances DNA damage-induced cytotoxicity. Thus, concurrent EGFR and BER blockade is superior to delayed BER inhibition alone. These findings collectively indicate that inhibition of either OGG1 or APE1 can reverse EGFR-TKI resistance in ER cells through modulation of BER pathway activity and associated molecular changes.

## Discussion

While targeted therapies have brought about a revolution in precision oncology, the rise of EGFR-TKI resistance continues to pose a major clinical challenge in the management of NSCLC. Notably, the non-heritable mechanisms driving this resistance remain poorly understood. In the present study, we demonstrate that OGG1 and APE1 enhance both EMT and stem-like plasticity in cancer cells, which in turn promotes tumor metastasis and therapeutic resistance. Our findings further establish that G4 structures, DNA base lesions, BER factors, and epigenetic remodeling cooperate to drive the transcriptional reprogramming that underlies cellular plasticity and TKI resistance in NSCLC.

Endogenous DNA damage and BER proteins play a key role in safeguarding genome integrity. Our findings, however, reveal another equally important function of BER proteins: they regulate gene expression by controlling the formation of DNA G4 structures. When we used the G4-specific 1H6 antibody for immunofluorescence staining, we found that downregulating APE1 completely eliminated G4 structure formation in cells. In contrast, upregulating APE1 or stabilizing G4 structures with the small molecule PDS boosted the transcription of EMT- and stemness-related DEGs, ultimately leading to the development of an EMT phenotype. Mapping analyses and biochemical evidence further support that APE1 binds to G4 structures and likely promotes their formation in promoter regions. This mechanism may serve as a way to facilitate the specific transcriptional programming of genes linked to EMT and stem-like plasticity. Thus, the observed pattern of G4 hotspots and their non-random distribution across genomic regions may not only reflect differences in DNA repair efficiency, but also point to a broader theme: connecting DNA damage to the regulation of gene expression. In this study, PDS monotherapy showed limited antitumor activity in xenografts derived from EGFR-TKI-sensitive parental cells, whereas combining PDS with erlotinib markedly suppressed tumor growth. These findings highlight the context-dependent action of PDS, consistent with its known efficacy in homologous recombination (HR)-deficient tumors such as those with BRCA1/2 mutations. Notably, the EGFR-TKI-sensitive parental cells used in our study possess a functional DNA damage repair (DDR) pathway, which we propose as the key underlying reason for the lack of efficacy of PDS monotherapy. PDS, as a G-quadruplex stabilizer, exerts its antitumor activity mainly by inducing DNA damage; however, a functional DDR pathway enables cells to repair such damage, thereby attenuating the therapeutic effect of PDS alone. In contrast, the combination of PDS with erlotinib overcomes this resistance: erlotinib, by targeting EGFR signaling, may interfere with DDR pathway function or enhance DNA damage induced by PDS, ultimately leading to robust tumor regression. These findings further confirm that the antitumor activity of PDS is highly context-dependent, with the cellular DDR background serving as a critical determinant.

The inherent redox sensitivity of guanines within G4 structures predisposes them to oxidative damage, facilitating localized lesion accumulation. Oxidative stress modulates BER enzyme activity (APE1 and OGG1) and G4 dynamics via multiple mechanisms: elevated ROS generate 8-oxoG lesions processed by APE1 and OGG1, while post-translational modifications and expression changes fine-tune enzyme function. Furthermore, intracellular conditions (e.g., pH and ion concentration) and direct guanine oxidation collectively influence G4 stability and conformation. Here, we identify a non-canonical role for APE1 in stabilizing G4 structures, elucidating a novel mechanism whereby oxidative stress regulates genomic architecture via G4-binding proteins. APE1 maintains genomic stability through dual roles in DNA repair and G4 stabilization, while OGG1 indirectly modulates G4 conformation via lesion processing. We propose that G4 structures function as oxidative stress sensors, where BER initiation within G4 sequences triggers signaling cascades regulating G4-dependent gene expression. G4 motifs are prevalent in promoters and telomeres—regions susceptible to oxidative damage and BER activity—yet their biological roles differ markedly. While telomeric G4s are prone to APE1 cleavage, their limited transcriptional impact suggests that promoter-specific G4 topology is critical for APE1-mediated gene regulation [45]. Structural analyses show that promoter G4-APE1 complexes stall to recruit transcription factors, whereas lower APE1 affinity for telomeric G4s permits efficient repair [46]. This topology-dependent specialization likely explains delayed damage processing in telomeres, contributing to cellular senescence, and opens new avenues for understanding G4 functions within DNA damage response networks.

In EGFR-TKI-resistant cells, we demonstrate that APE1 promotes the transition of G4s from transient to stable states by binding to PQSs within open chromatin promoters. Genome-wide binding profiles show significant APE1-G4 co-localization in promoters, confirmed by immunofluorescence co-localization assays. Functional studies establish APE1 as a direct G4-binding protein with preferential affinity for G4 structures located in the promoters of EMT- and stemness-related genes. Both APE1 knockdown and G4-stabilizing agent MX treatment alter cellular G4 levels, underscoring APE1’s central role in G4 dynamics. While BG4-CUT&Tag and 1H6 immunofluorescence are standard approaches for G4 detection, their inherent limitations in conformational specificity and spatial resolution introduce methodological biases that warrant consideration. Specifically, the BG4 antibody used in CUT&Tag exhibits a preference for parallel G4 structures [47] —the predominant genomic conformation under physiological conditions—while showing substantially reduced recognition of antiparallel and hybrid conformations. This bias may lead to an underrepresentation of non-parallel G4 abundance and distribution in our dataset [48]. Furthermore, signal intensity is contingent upon chromatin accessibility. Consequently, G4s embedded in compact heterochromatin are less accessible to the antibody, potentially leading to incomplete detection or underestimation of G4s in transcriptionally silent regions [49]. The 1H6 antibody suffers from significant specificity limitations that compromise its reliability for detecting genomic G4s via immunofluorescence. Biochemical evidence indicates that 1H6 cross-reacts prominently with thymidine-rich single-stranded DNA (particularly poly(T) sequences), but not with poly(C) or poly(A) [50]. Its G4 recognition is sequence-dependent: it binds strongly to T4G4-containing structures but weakly to those lacking thymidine (e.g., T2G4). This generates non-specific background noise that complicates, and in some cases precludes, accurate interpretation of nuclear G4 signals [50]. Moreover, 1H6 cannot distinguish between DNA and RNA G4s, rendering the assay susceptible to RNA G4 interference [51]. Immunofluorescence permits only single-cell visualization and quantification of global G4 levels; it lacks high-resolution genomic positioning and the capacity to resolve signals from specific gene loci, thereby precluding locus-specific validation [52]. These current technical limitations in detecting diverse G4 topologies necessitate future studies combining PQS motif analysis with multi-conformational G4 detection approaches.

We further identified a significant enrichment of AP-1 transcription factor binding sites proximal to folded G4 structures, suggesting an epigenetic regulatory mechanism whereby G4s facilitate the recruitment of specific transcription factors. AP-1, a heterodimer composed of JUN and FOS family proteins, integrates extracellular and environmental cues to regulate target gene transcription; notably, its DNA-binding affinity is modulated by CGI methylation status. Our WGBS analysis reveals that G4 structures protect CGIs from DNA methylation: hypomethylated CGIs frequently harbor G4s, whereas their flanking regions exhibit significantly higher methylation levels. We speculate that G4 formation within chromatin creates a permissive landscape for AP-1 recruitment, potentially orchestrated by APE1 [53]. A key limitation of this study is the absence of direct epigenetic profiling, which precludes the establishment of causal models defining DNA methylation as either an upstream regulator or a downstream effector of G4-mediated transcription dynamics; addressing these gaps represents a priority for future research.

Based on existing literature, we propose that G4 structure formation is a key driver of CGI hypomethylation. Previous studies have documented G4 formation in gene promoters and reported high-affinity in vitro binding of recombinant human DNMTs to G4-containing DNA [54]. Furthermore, G4-forming sequences (G4FS) inherently elicit hypomethylation, an effect modulated by G4 stability, chromatin accessibility, localization within CGIs, and proximity to CpG sites [55–57]. Consistently, G4 structures detected via BG4-CUT&Tag and 1H6 immunofluorescence were significantly enriched in open chromatin regions, indicative of high folding potential and an anti-methylation function. The co-localization of G4s with hypomethylated CGIs supports their role in establishing a methylation-non-permissive microenvironment, likely through DNMTs inhibition, thereby preventing aberrant methylation. Notably, we hypothesize a bidirectional regulatory relationship between G4s and hypomethylated CGIs. Previous studies have indicated that G4-associated chromatin is characterized by nucleosome depletion and an open conformation [58]; consequently, hypomethylated CGIs, especially those in open chromatin, are prone to enrichment with G4FS possessing high folding potential. Thus, hypomethylation itself may enhance G-rich sequence accessibility by reducing chromatin compaction, thereby facilitating G4 formation. Moreover, it is also identified that G4-induced hypomethylation is highly context-dependent. This context-dependence elucidates the G4 localization patterns observed in our study: detected G4s were predominantly located in open chromatin regions. This aligns with the preference of BG4-CUT&Tag and 1H6 for accessible G4s, which coincide with regions where hypomethylation is most pronounced. Additionally, G4-mediated hypomethylation is influenced not only by CpG density but also synergizes with transcriptional activity and transcription factor binding. These findings reinforce the view of G4s as functional regulators of methylation, rather than passive byproducts.

The therapeutic synergy between APE1/OGG1 inhibitors and targeted agents represents a promising frontier in oncology. Our in vivo studies show that co-targeting APE1 and EGFR signaling overcomes acquired TKI resistance, supporting the rationale for combining DNA repair modulation with pathway-specific inhibition. Concurrent blockade of EGFR and BER pathways proves superior to delayed BER inhibition alone. A key limitation is that pre-established xenografts do not fully recapitulate de novo resistance; future investigations into clonal evolution under erlotinib pressure are warranted to validate these findings in more clinically relevant settings. To date, a growing body of research has provided preliminary evidence for this. At the DNA repair level, APE1/OGG1 inhibition impairs BER, creating synthetic lethality with PARP inhibitor-mediated SSB repair blockade, particularly in replication-stressed, homologous recombination-deficient cancers [59]. Beyond DNA repair modulation, APE1/OGG1 inhibition potentiates immune checkpoint blockade by inducing micronuclei formation and subsequent cytosolic DNA release [60]; this activates the cGAS-STING pathway, triggering type I interferon production and dendritic cell activation [61]. This cascade ultimately enhances tumor immunogenicity via interferon-mediated upregulation of MHC class I expression and neoantigen presentation, thereby facilitating cytotoxic T lymphocyte (CTL) recognition and infiltration into immunologically cold tumors. Collectively, these dual mechanisms establish APE1/OGG1 inhibition as a rational combinatorial strategy to overcome therapeutic resistance through both intrinsic (DNA repair) and extrinsic (immune-mediated) pathways. These results substantiate the therapeutic potential of concomitant targeting of DNA repair machinery and oncogenic signaling pathways.

**Fig. 9:**
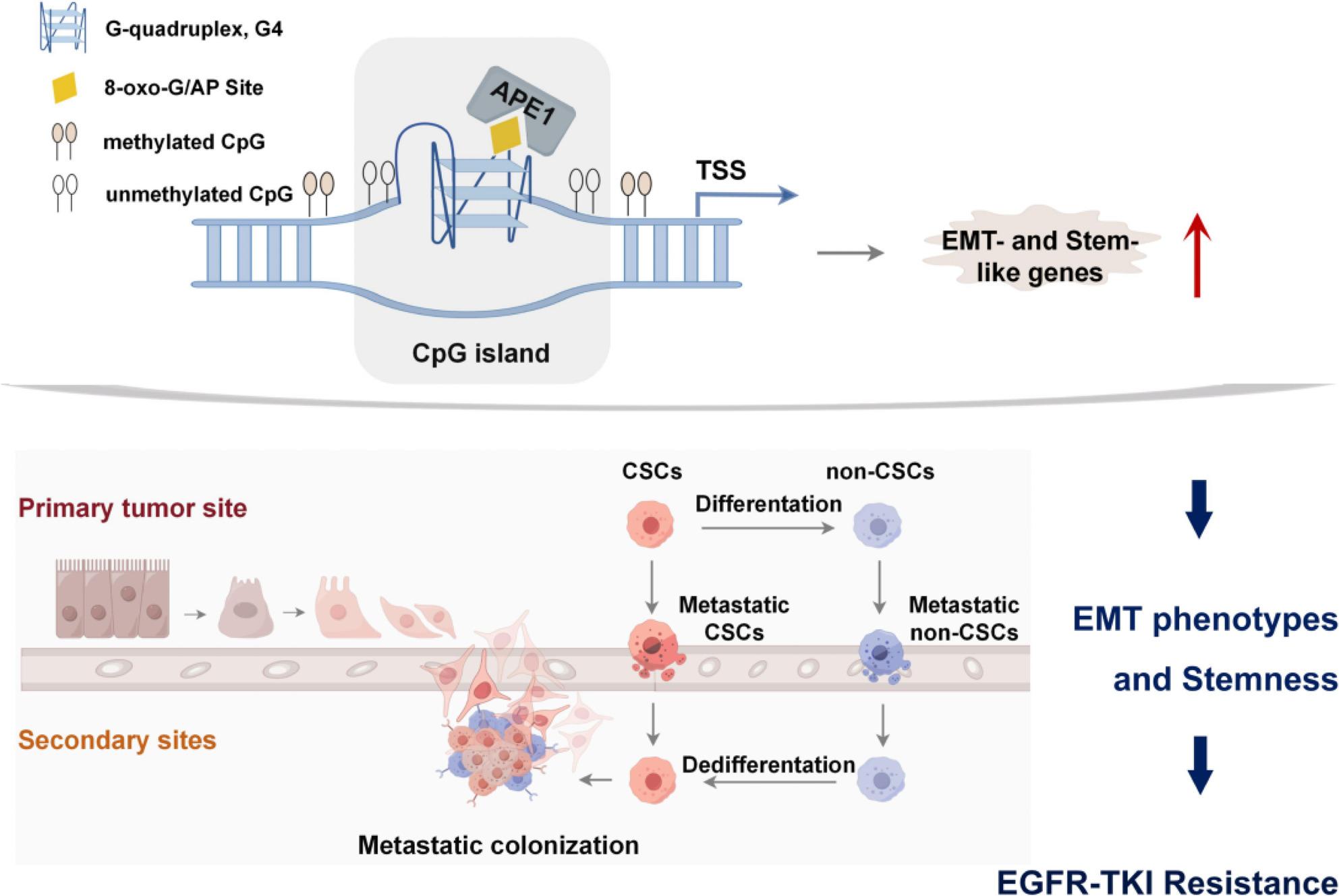
Proposed schematic representation. Proposed schematic representation of endogenous oxidative DNA damage orchestrates APE1-mediated G4 stabilization at promoter regions of EMT and stemness-related genes, driving transcriptome reprogramming through epigenetic regulation. Notably, the enrichment of G4 structures within hypomethylated CGIs implies a functional relationship between secondary DNA structures and DNA methylation dynamics, which may play a regulatory role in gene expression control. Specifically, APE1 binding to PQS facilitates G4 structure formation, which in turn serves as an epigenetic scaffold for sustained expression of EMT and stem-like plasticity genes. This BER-mediated epigenetic remodeling represents a critical molecular determinant of acquired TKI resistance, highlighting the functional interplay between DNA damage response, chromatin architecture, and lineage plasticity in therapeutic resistance

Using an integrated multi-omics approach, we have elucidated a novel mechanism wherein DNA base lesions orchestrate the APE1-mediated stabilization of G4 structures at the promoters of EMT- and stemness-related genes, driving transcriptome reprogramming (Fig. 9). Notably, the enrichment of G4 structures within hypomethylated CpG islands reveals a functional relationship between DNA secondary structures and epigenetic dynamics. Mechanistically, APE1 binding to PQSs facilitates G4 formation; these structures, in turn, act as epigenetic scaffolds that sustain the expression of genes associated with cellular plasticity. This BER-mediated epigenetic remodeling defines the functional nexus linking DNA damage response, chromatin architecture, and cellular plasticity in the context of acquired TKI resistance.

## Conclusions

EGFR-TKI-resistant NSCLC cells acquire EMT and stem-like plasticity, a process driven by the accumulation of oxidative DNA damage and subsequent BER activation via OGG1/APE1 upregulation, which correlates with poor patient prognosis. Genome-wide profiling revealed a strategic redistribution of OGG1/APE1 binding sites and G4 structures at EMT- and stemness-associated loci, characterized by the preferential binding of APE1 to G4-enriched promoters. Mechanistically, APE1 orchestrates G4-mediated transcriptional activation of EMT/stemness programs, particularly within hypomethylated CGIs at plasticity-associated loci. Therapeutically, combinatorial BER inhibition synergized with EGFR-TKIs to resensitize resistant cells and suppress xenograft tumor growth in vivo, establishing APE1-G4 axis targeting as a promising strategy to overcome TKI resistance.

## Supplementary Information

Supplementary Material 1.

Supplementary Material 2.

Supplementary Material 3.

## Data Availability

No datasets were generated or analysed during the current study.

## Abbreviations

APE1: Apurinic/Apyrimidinic Endonuclease 1
AP: Apurinic/Apyrimidinic Site
AP-1: Activator Protein-1
ATAC-seq: Assay for Transposase-Accessible Chromatin using sequencing
BER: Base Excision Repair
BG4 anti‑G4 antibody: Anti‑DNA/RNA G‑quadruplex Structures Antibody, clone BG4
CGI: CpG Island (plural: CGIs)
ChIP-qPCR: Chromatin Immunoprecipitation Quantitative Polymerase Chain Reaction
CUT&Tag-seq: Cleavage Under Targets and Tagmentation sequencing
CTL: Cytotoxic T Lymphocyte
DAR: Differentially Accessible Region (plural: DARs)
DSB: Double-Strand Break
DNMT: DNA Methyltransferase (plural: DNMTs)
EGFR: Epidermal Growth Factor Receptor
EGFR-TKI: Epidermal Growth Factor Receptor Tyrosine Kinase Inhibitor
EMSA: Electrophoretic Mobility Shift Assay
EMT: Epithelial-Mesenchymal Transition
ER: Erlotinib-Resistant
GO: Gene Ontology
G4: G-quadruplex
GSEA: Gene Set Enrichment Analysis
IHC: Immunohistochemistry
IGV: Integrative Genomics Viewer
KD: Knockdown
KEGG: Kyoto Encyclopedia of Genes and Genomes
LAFC: Laser Confocal Fluorescence Microscopy
LUAD: Lung Adenocarcinoma
MAPK: Mitogen-Activated Protein Kinase
MX: Methoxyamine
mIHC: Multiplex Immunohistochemistry
NES: Normalized Enrichment Score
NSCLC: Non-Small Cell Lung Cancer
OE: Overexpression
OGG1: 8-Oxoguanine DNA Glycosylase 1
ORR: Objective Response Rate
PDS: Pyridostatin
PFS: Progression-Free Survival
PQS: Potential G4 Forming Sequences
qPCR: Quantitative Polymerase Chain Reaction
Ref-1: Redox Factor-1 (alias of APE1)
RNA-seq: RNA Sequencing
ROS: Reactive Oxygen Species
Sanger-seq: Sanger Sequencing
SSiNGLe-AP: Single Nucleotide Resolution Abasic Site Mapping by Ligation-Mediated PCR
TSS: Transcription Start Site
TFBS: Transcription Factor Binding Site (plural: TFBSs)
UTR: Untranslated Region (e.g., 5′ UTR = 5′ Untranslated Region)
WB: Western Blot
WGBS: Whole-Genome Bisulfite Sequencing
WGS: Whole-Genome Sequencing
