# Supplementary material for: DNA base lesion-containing G-quadruplex mediates transcriptome reprogramming in EGFR-TKI resistance of non-small cell lung cancer
Source: J Exp Clin Cancer Res. 2026 Apr 14;45:122. doi: 10.1186/s13046-026-03702-w (PMC13185293; doi:10.1186/s13046-026-03702-w)

Extended Data Figure1

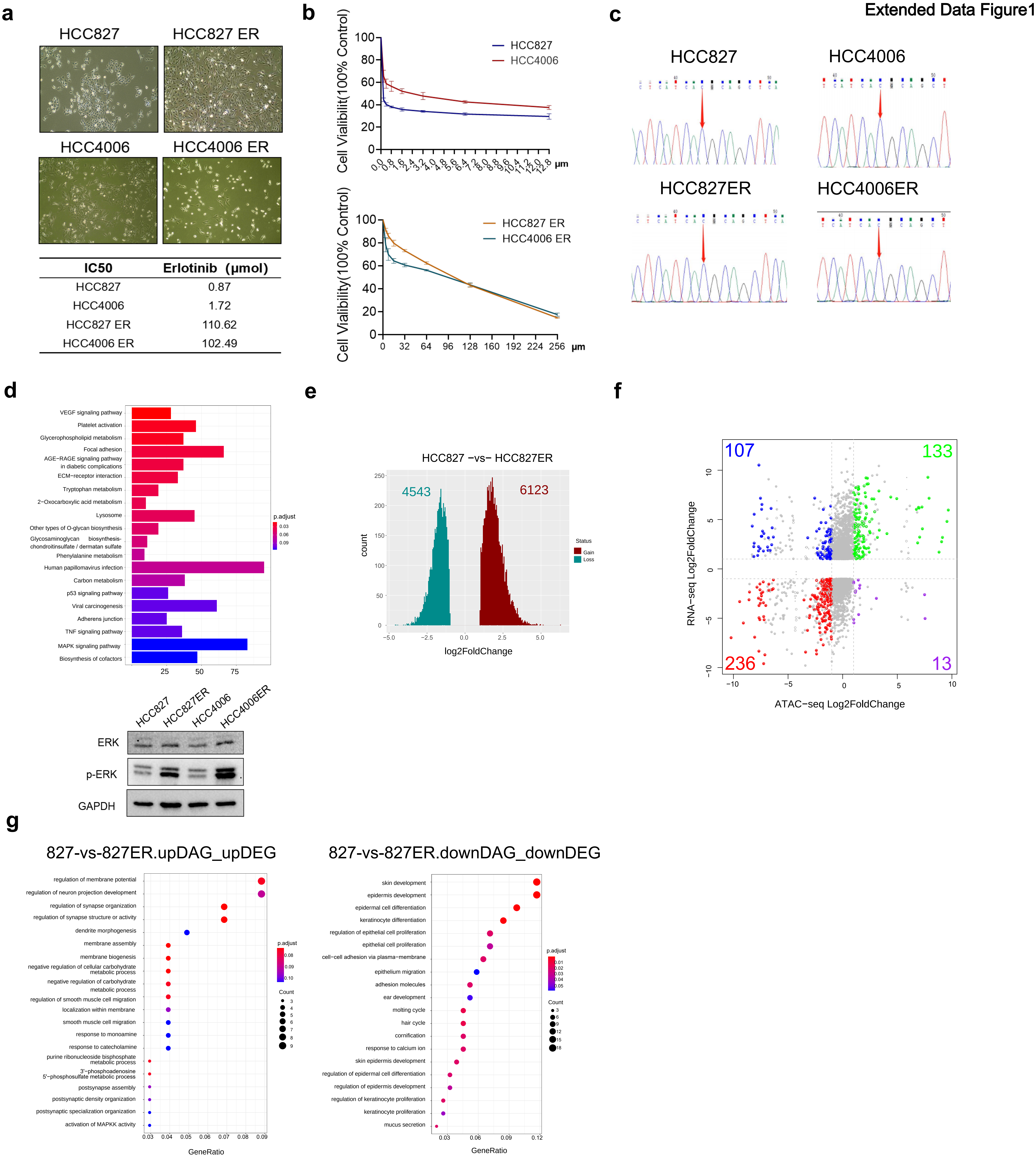

IC50	Erlotinib (μmol)
HCC827	0.87
HCC4006	1.72
HCC827 ER	110.62
HCC4006 ER	102.49

Extended Data Figure2

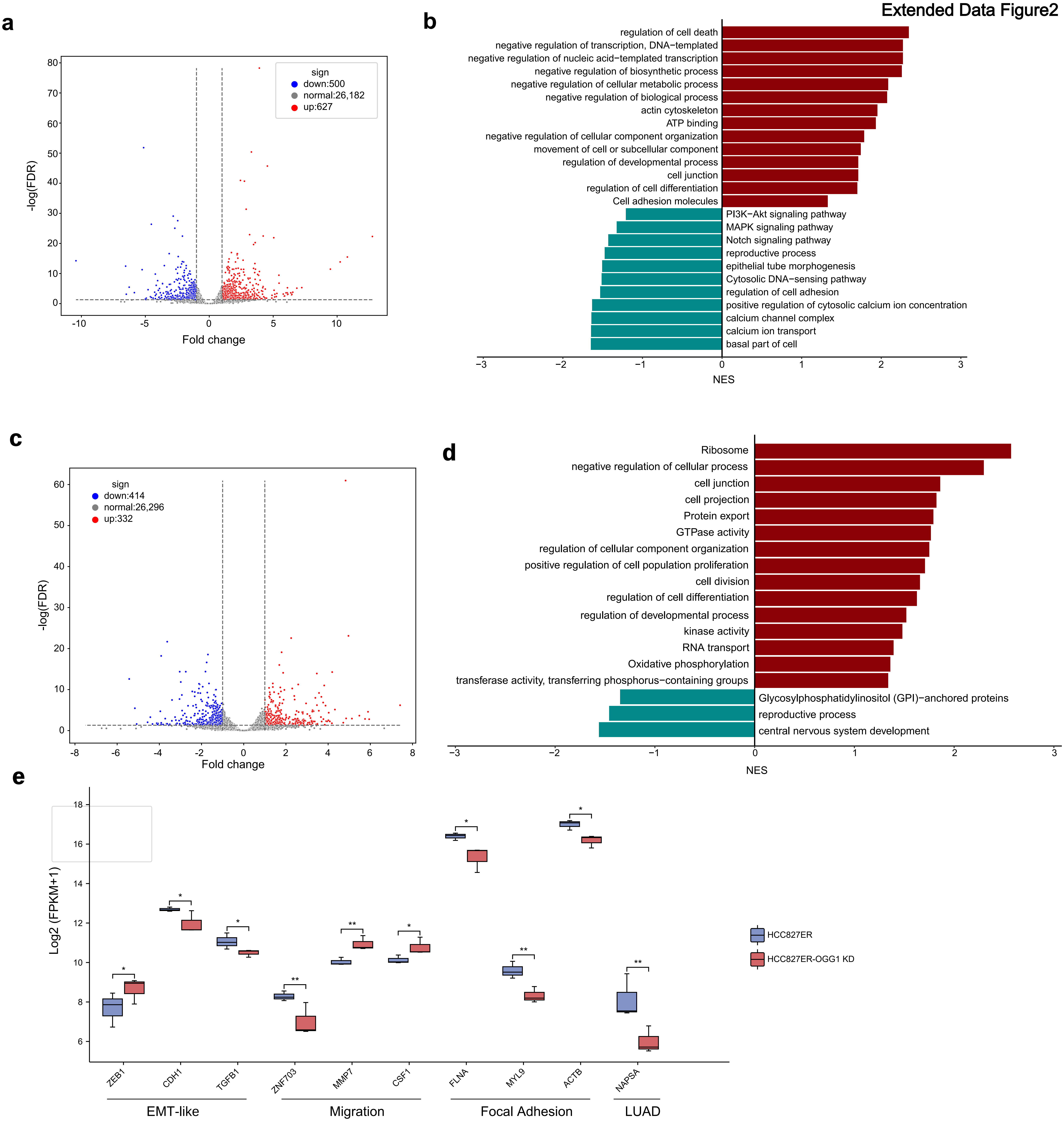

Extended Data Figure3

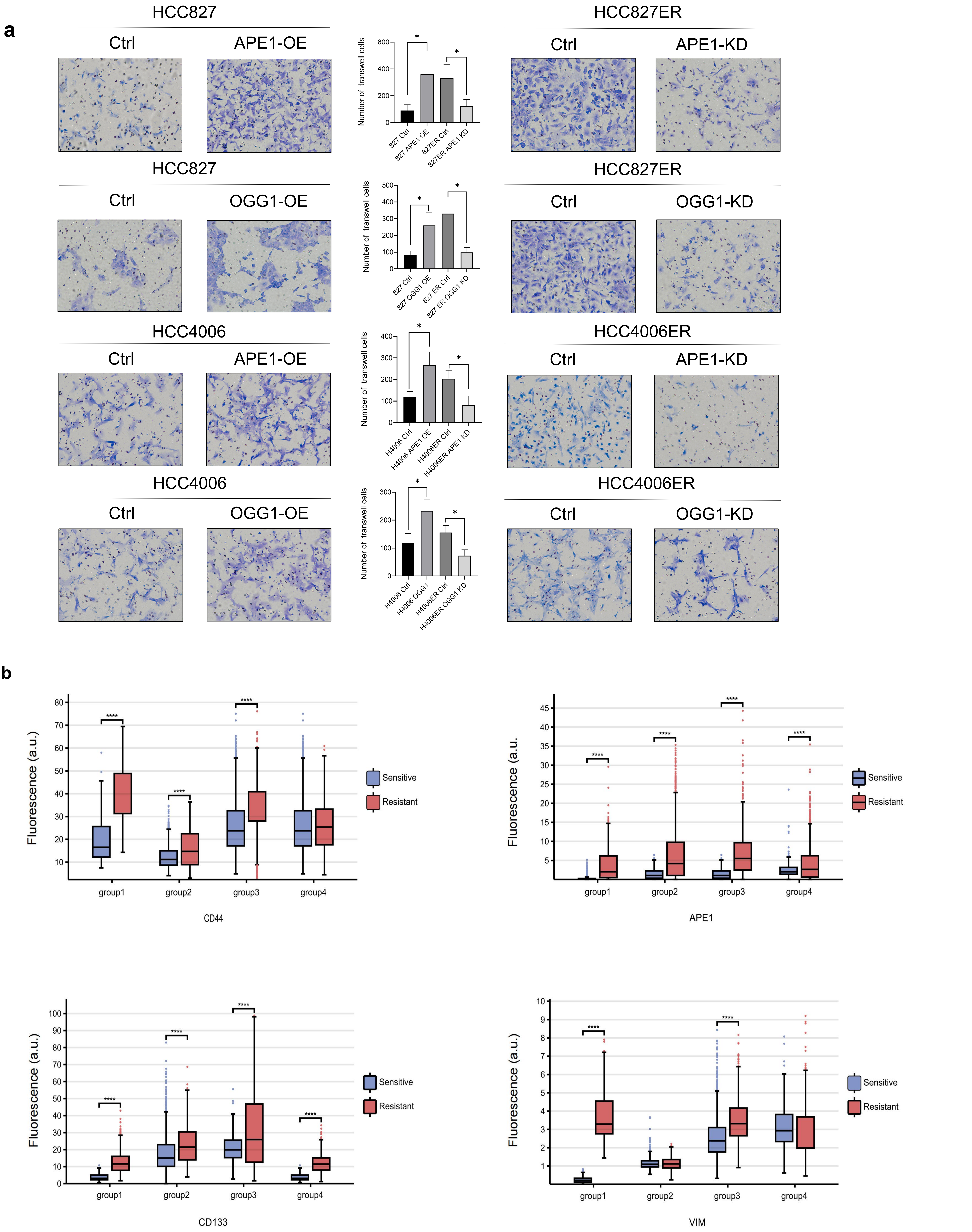

Extended Data Figure4

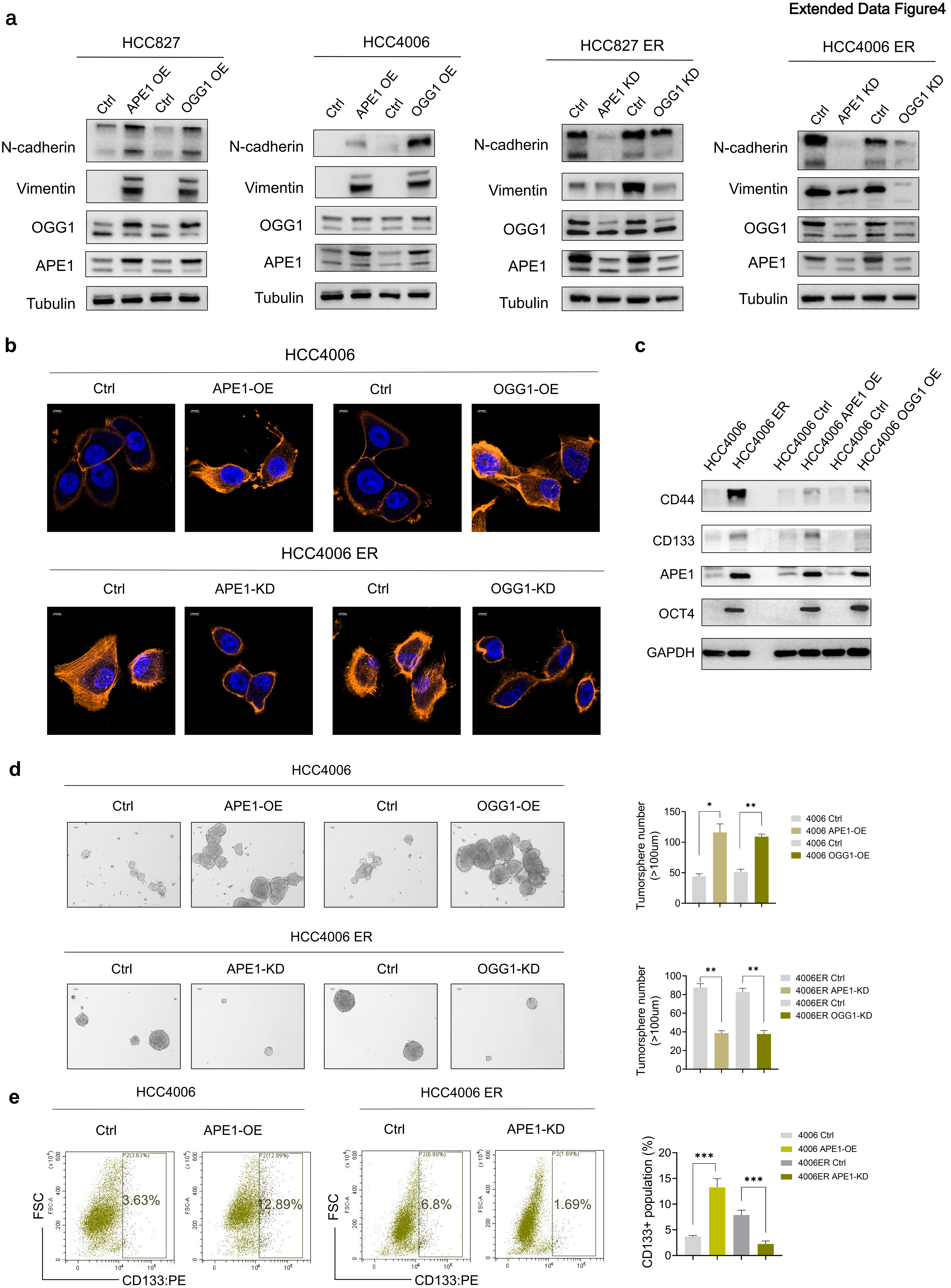

Extended Data Figure5

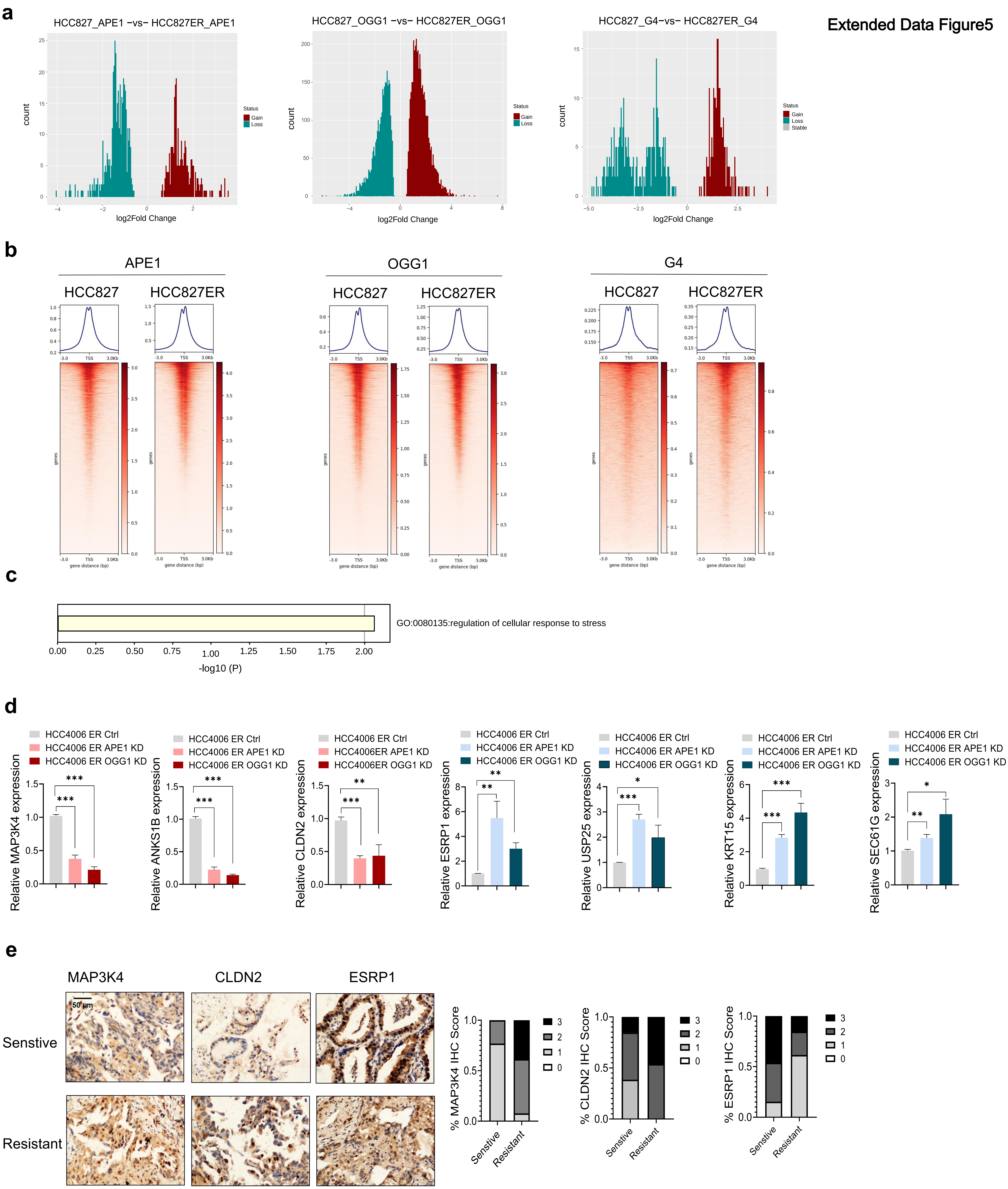

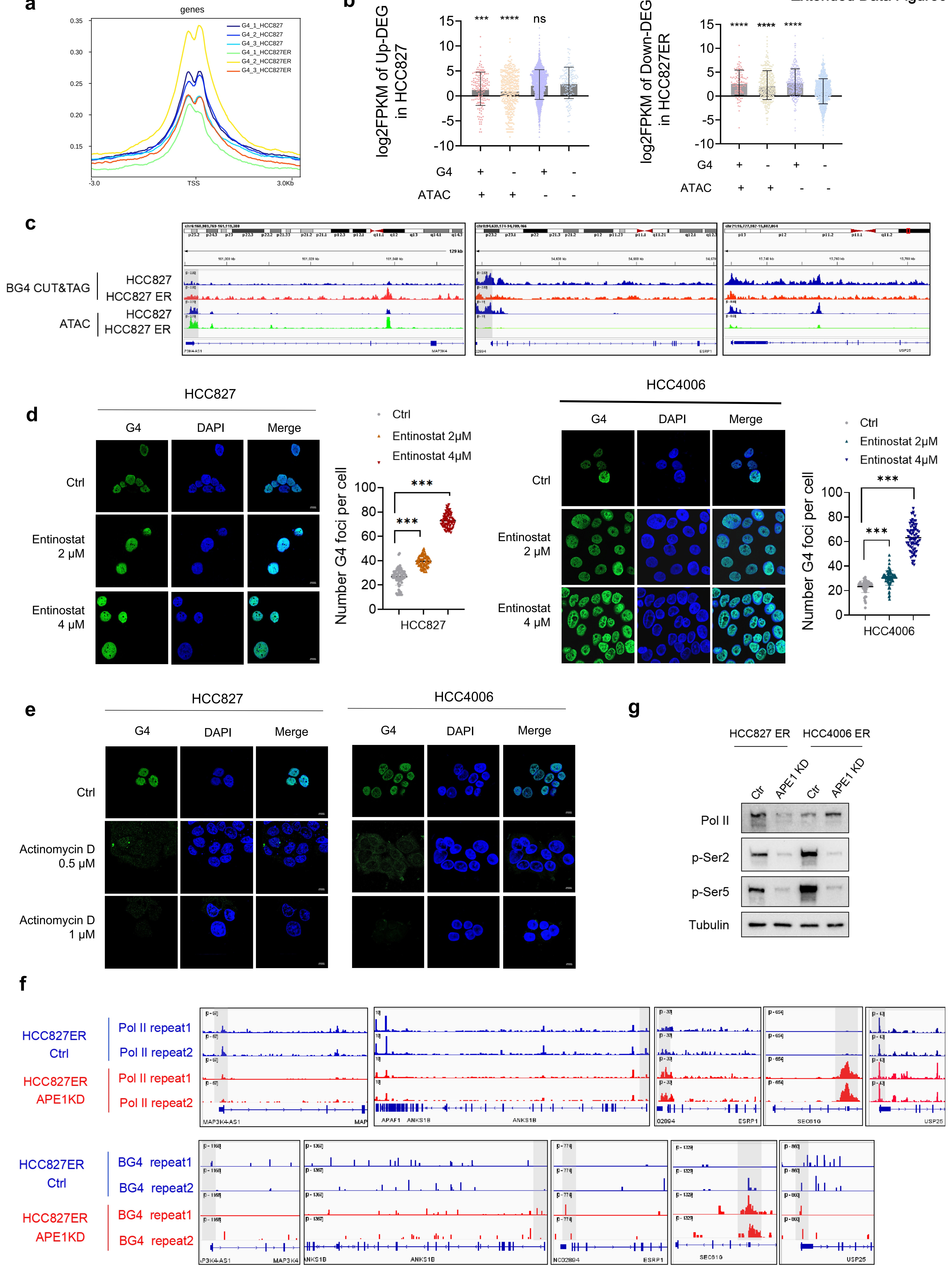
a
genes
G4_1_HCC827
G4_2_HCC827
G4_3_HCC827
G4_1_HCC827ER
G4_2_HCC827ER
G4_3_HCC827ER
-3.0
TSS
3.0Kb
b
log2FPKM of Up-DEG in HCC827
log2FPKM of Down-DEG in HCC827ER
G4
ATAC
*** **** ns
**** **** ****
c
BG4 CUT&TAG
HCC827
HCC827 ER
ATAC
HCC827
HCC827 ER
d
HCC827
HCC4006
G4
DAPI
Merge
Ctrl
Entinostat 2 μM
Entinostat 4 μM
Ctrl
Entinostat 2μM
Entinostat 4μM
Number G4 foci per cell
***
e
Actinomycin D 0.5 μM
Actinomycin D 1 μM
g
HCC827 ER
HCC4006 ER
Ctr
APE1 KD
Pol II
p-Ser2
p-Ser5
Tubulin
f
HCC827ER Ctrl
HCC827ER APE1KD
Pol II repeat1
Pol II repeat2
BG4 repeat1
BG4 repeat2
MAP3K4-AS1
MAP3K4
APAF1
ANKS1B
ESRP1
SEC61G
USP25

Extended Data Figure7

a

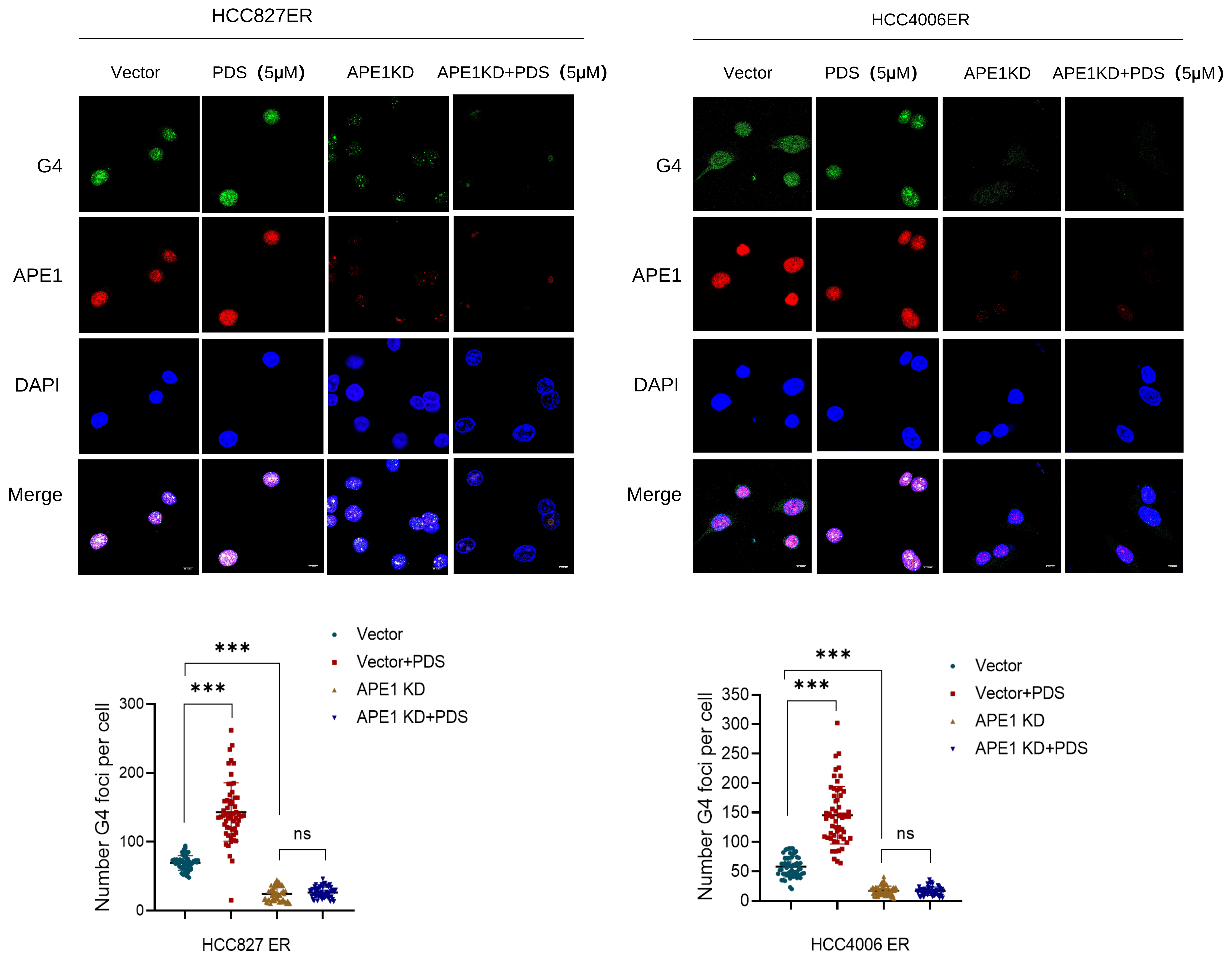

b

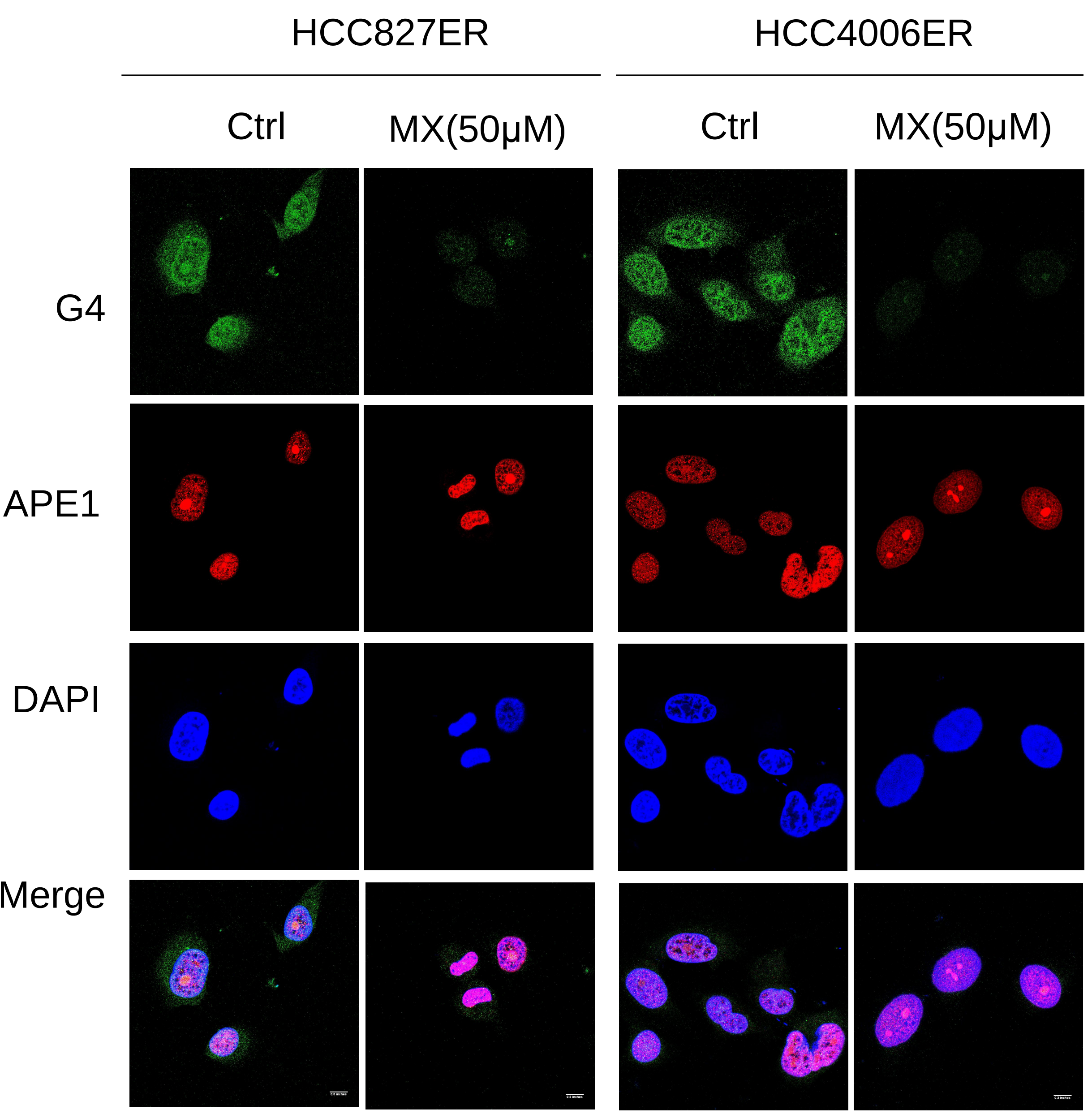

c

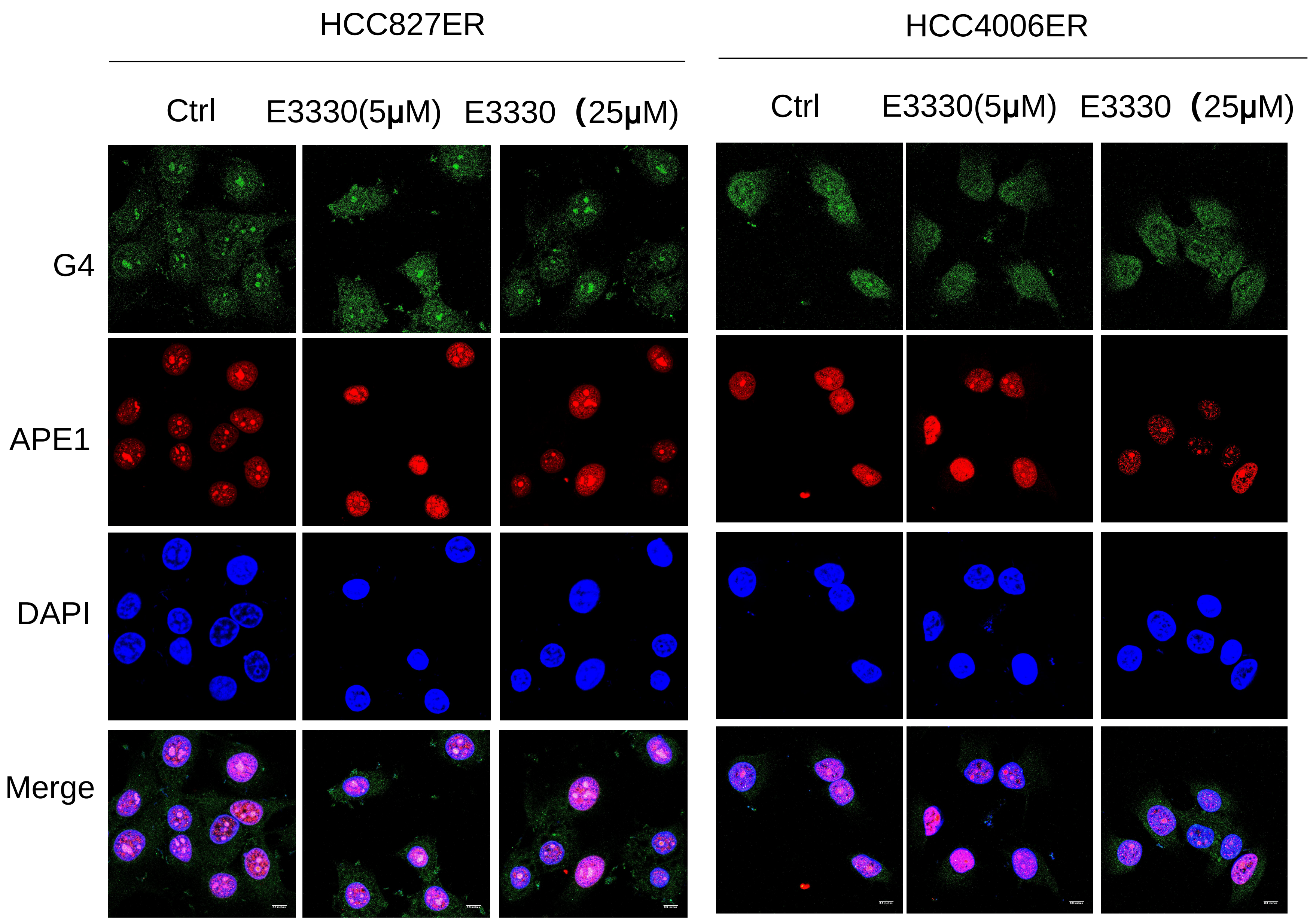

Extended Data Figure8

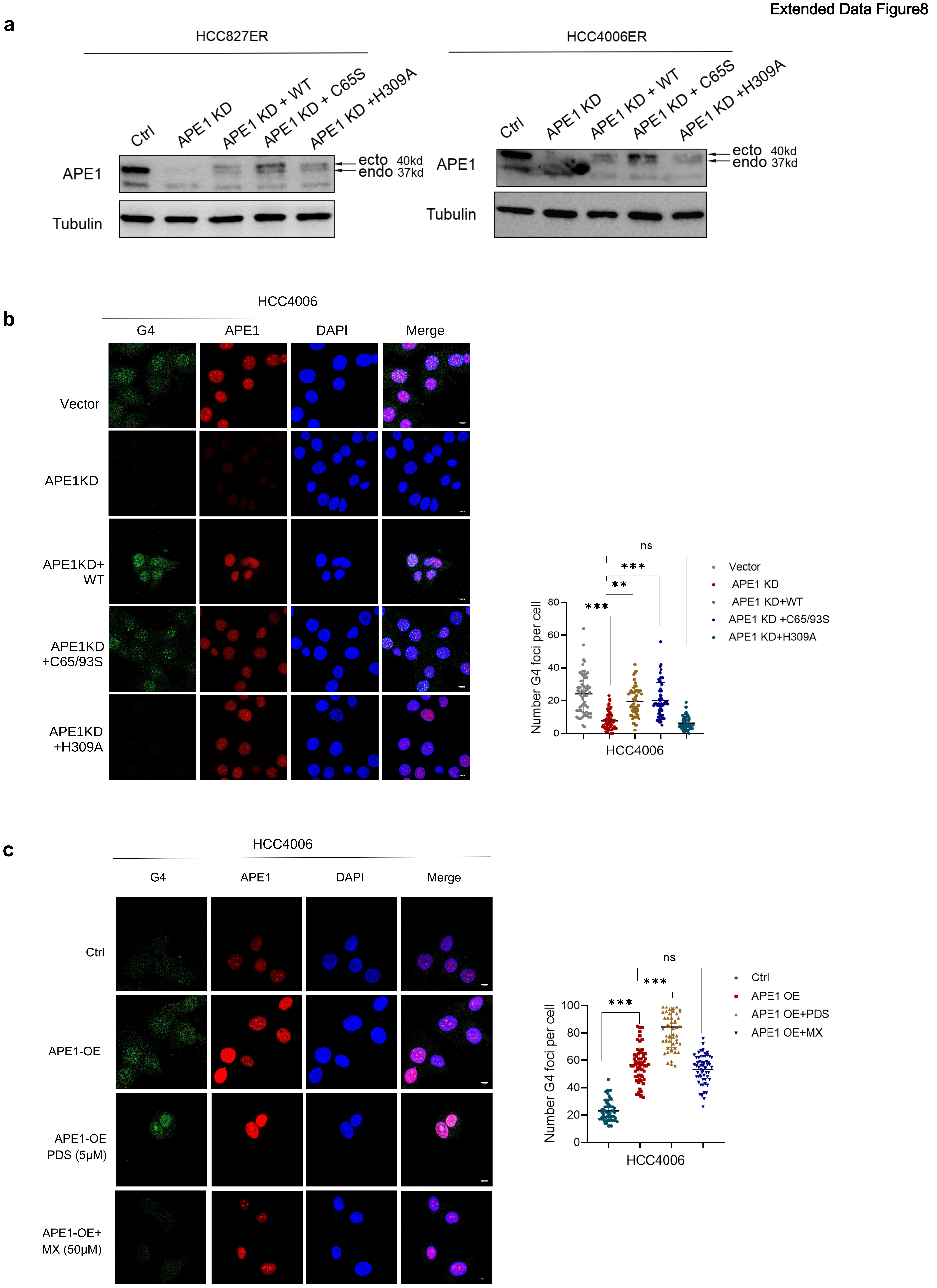

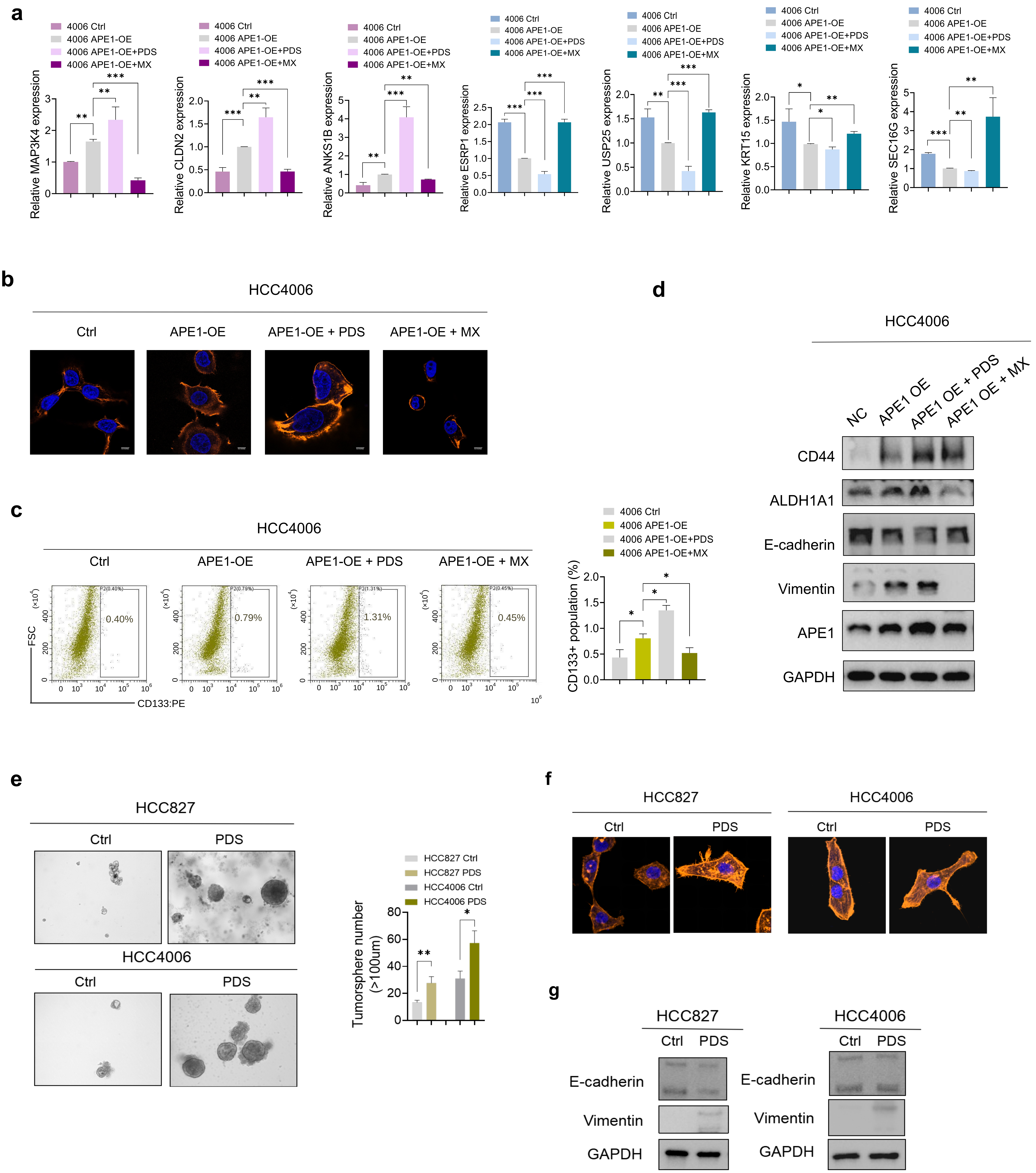
a
4006 Ctrl
4006 APE1-OE
4006 APE1-OE+PDS
4006 APE1-OE+MX
Relative MAP3K4 expression
Relative CLDN2 expression
Relative ANKS1B expression
Relative ESRP1 expression
Relative USP25 expression
Relative KRT15 expression
Relative SEC16G expression
b
HCC4006
Ctrl
APE1-OE
APE1-OE + PDS
APE1-OE + MX
c
HCC4006
0.40%
0.79%
1.31%
0.45%
FSC
CD133:PE
CD133+ population (%)
d
HCC4006
NC
APE1 OE
APE1 OE + PDS
APE1 OE + MX
CD44
ALDH1A1
E-cadherin
Vimentin
APE1
GAPDH
e
HCC827
Ctrl
PDS
HCC4006
HCC827 Ctrl
HCC827 PDS
HCC4006 Ctrl
HCC4006 PDS
Tumorsphere number (>100um)
f
HCC827
HCC4006
g
E-cadherin
Vimentin
GAPDH

Extended Data Figure10

a

b

c

d

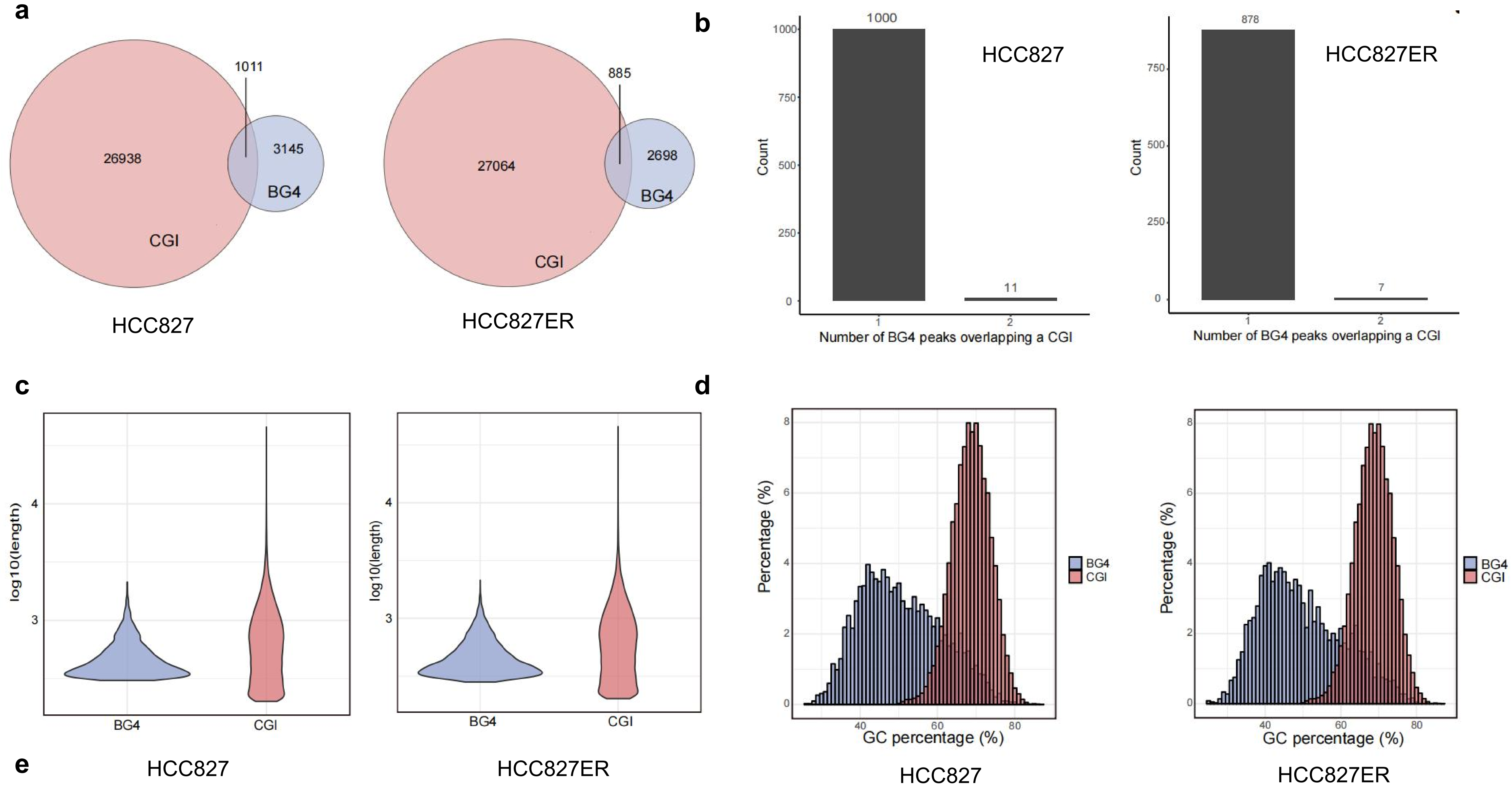

e

Gene / CpG Island	MAP3K4	CLDN2	ANKS1B	ESRP1	USP25	KRT15	SEC61G
Location	160,991,198... 160,992,666	non	100,141,972... 100,143,095	94,641,510... 94,642,701	15,729,458... 15,730,789	non	54,758,646... 54,759,634
Length	1469nt		1124nt	1192nt	1332nt		989nt
Location				94,640,098... 94,641,088			
Length				991nt			
Location				94,638,817... 94,639,756			
Length				940nt			

f

Homer Known Motif Enrichment Results (G4_HCC827-vs-G4_HCC827ER.GainDP)

Rank	Motif	Name	P-value	log P-pvalue	% of Targets Sequences with Motif	% of Background Sequences with Motif
1		Fra1(bZIP)/BT549-Fra1-ChIP-Seq(GSE46166)/Homer	1e-17	-3.937e+01	22.27%	5.06%
2		AP-1(bZIP)/ThioMac-PU.1-ChIP-Seq(GSE21512)/Homer	1e-16	-3.895e+01	26.07%	7.02%
3		Fos(bZIP)/TSC-Fos-ChIP-Seq(GSE110950)/Homer	1e-15	-3.611e+01	22.27%	5.51%
4		Fra2(bZIP)/Striatum-Fra2-ChIP-Seq(GSE43429)/Homer	1e-15	-3.463e+01	18.96%	4.14%
5		Atf3(bZIP)/GBM-ATF3-ChIP-Seq(GSE33912)/Homer	1e-15	-3.463e+01	23.22%	6.22%
6		BATF(bZIP)/Th17-BATF-ChIP-Seq(GSE39756)/Homer	1e-14	-3.302e+01	22.75%	6.24%
7		JunB(bZIP)/DendriticCells-Junb-ChIP-Seq(GSE36099)/Homer	1e-14	-3.270e+01	20.38%	5.08%
8		Fosl2(bZIP)/3T3L1-Fosl2-ChIP-Seq(GSE56872)/Homer	1e-12	-2.862e+01	13.74%	2.59%
9		Jun-AP1(bZIP)/K562-cJun-ChIP-Seq(GSE31477)/Homer	1e-11	-2.695e+01	11.37%	1.84%
10		HNF1b(Homeobox)/PDAC-HNF1B-ChIP-Seq(GSE64557)/Homer	1e-7	-1.738e+01	9.00%	1.89%

Homer Known Motif Enrichment Results (G4_HCC827-vs-G4_HCC827ER.LossDP)

Rank	Motif	Name	P-value	log P-pvalue	% of Targets Sequences with Motif	% of Background Sequences with Motif
1		Fos(bZIP)/TSC-Fos-ChIP-Seq(GSE110950)/Homer	1e-3	-8.324e+00	9.27%	4.52%
2		IRF2(IRF)/Erythroblas-IRF2-ChIP-Seq(GSE36985)/Homer	1e-3	-7.252e+00	2.56%	0.60%
3		JunB(bZIP)/DendriticCells-Junb-ChIP-Seq(GSE36099)/Homer	1e-2	-6.586e+00	8.31%	4.35%
4		RAR:RXR(NR),DR5/ES-RAR-ChIP-Seq(GSE56893)/Homer	1e-2	-6.550e+00	1.60%	0.26%
5		IRF1(IRF)/PBMC-IRF1-ChIP-Seq(GSE43036)/Homer	1e-2	-6.544e+00	2.88%	0.83%
6		Jun-AP1(bZIP)/K562-cJun-ChIP-Seq(GSE31477)/Homer	1e-2	-6.185e+00	4.47%	1.82%
7		Foxh1(Forkhead)/hESC-FOXH1-ChIP-Seq(GSE29422)/Homer	1e-2	-6.184e+00	9.58%	5.44%
8		AP-1(bZIP)/ThioMac-PU.1-ChIP-Seq(GSE21512)/Homer	1e-2	-6.016e+00	10.22%	5.99%
9		Fosl2(bZIP)/3T3L1-Fosl2-ChIP-Seq(GSE56872)/Homer	1e-2	-5.904e+00	5.43%	2.51%
10		FOXM1(Forkhead)/MCF7-FOXM1-ChIP-Seq(GSE72977)/Homer	1e-2	-5.857e+00	13.74%	8.87%

Extended Data Figure11

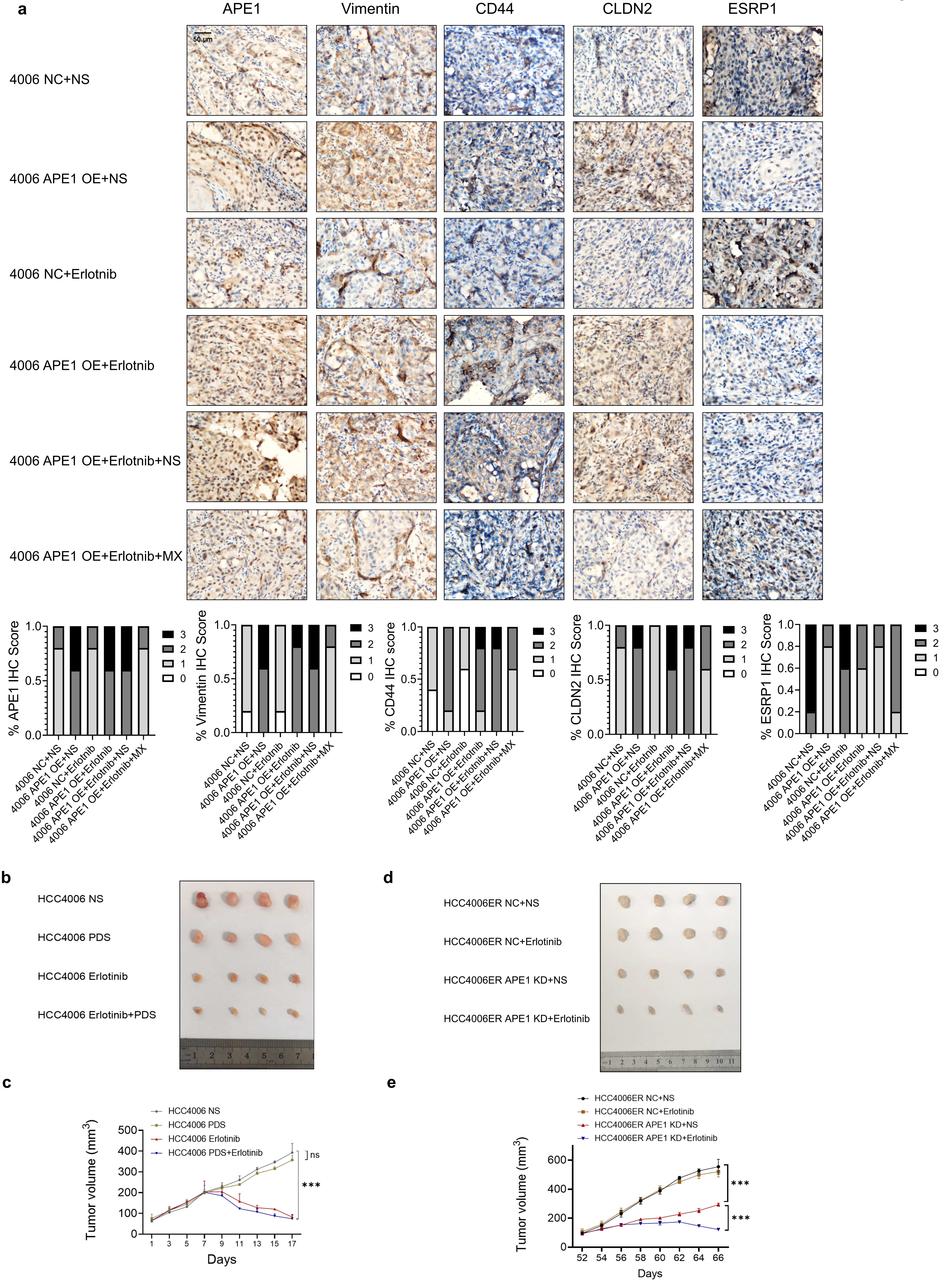

Supplement: Supplementary file 1 — Supplementary Material 1. [file 13046_2026_3702_MOESM1_ESM.pdf]
