# Supplementary material for: DNA base lesion-containing G-quadruplex mediates transcriptome reprogramming in EGFR-TKI resistance of non-small cell lung cancer
Source: J Exp Clin Cancer Res. 2026 Apr 14;45:122. doi: 10.1186/s13046-026-03702-w (PMC13185293; doi:10.1186/s13046-026-03702-w)
Supplement: Supplementary file 2 — Supplementary Material 2. [file 13046_2026_3702_MOESM2_ESM.docx]

**Extended Data Materials and Methods**

**Cell culture and treatment.** Our laboratory has independently generated cell lines that are either sensitive or resistant to erlotinib. We performed comprehensive analyses of these lines, as described later in the manuscript. Both HCC827 (Exon19 del) and HCC4006 (Exon19 del) cells were maintained in RPMI 1640 medium (Procell, PM150110) supplemented with 10% fetal bovine serum (Procell, 164210) and incubated in a 5% CO2 atmosphere at 37 ℃. Cells were passaged using 0.25% trypsin-EDTA (Procell, PB180226). To generate Erlotinib-resistant HCC827 and HCC4006 cells (referred to as ER cells), we implemented a sequential approach wherein increasing concentrations of erlotinib (MCE, HY-50896) were administered to sensitive parental cells. This protocol was repeated until the IC50 of the ER cells increased by more than tenfold compared to their sensitive counterparts, as verified by dose-response curves and cell viability assays. Resistant cells were continuously cultured at their respective final concentrations of erlotinib to maintain resistance. Prior to subsequent drug experiments, ER cells were washed three times with PBS and incubated in drug-free medium for one week to reduce drug carry-over effects.

**Lentiviral and cell transfection.** Lentiviruses encoding OGG1 and APE1 overexpression or knockdown and negative controls were designed and produced by GENECHEM (Shanghai, China). Further transfection was performed according to the manufacturer’s protocol. The lentivirus was added to the parental and resistant cells (overexpressing APE1/OGG1 in HCC827/HCC4006 cells and knockdown of the same genes in HCC827ER/HCC4006ER cells), and stable cell lines were obtained by selection with puromycin (Sigma-Aldrich, MO, USA). Infection efficiency was confirmed by fluorescence microscopy and real-time quantitative RT-PCR (qRT-PCR). The sequences for OE and KD are followed: LV-APEX1-RNAi/NC:TTCTCCGAACGTGTCACGT; LV-APEX1-RNAi:TGCCACACTCAAGATCTGC;LV-OGG1-RNAi/NC:TTCTCCGAACGTGTCACGT; LV-OGG1-RNAi:TTCCAAGAGGTGGCTCAGAAA; LV-APEX1-OE:(forward: 5'-AGGTCGACTCTAGAGGATCCCGCCACCATGCCGAAGCGTGGGAAAAAG-3'; reverse: 5'-TCCTTGTAGTCCATACCCAGTGCTAGGTATAGGGTGATAG-3'); LV-OGG1-OE: (forward: 5'-CACACTGGACTAGTGGATCCCGCCACCATGCCTGCCCGCGCGCTTCTG-3'; reverse: 5'-AGTCACTTAAGCTTGGTACCGAGCCTTCCGGCCCTTTGGAACCCTTTC-3')

**Cleavage Under Target and tagmentation (CUT&Tag).** The CUT&Tag protocol was conducted as previously described^1^, using the Hyperactive Universal CUT&Tag Assay Kit for Illumina Pro (Vazyme, TD904) and 1 μg of specific antibodies: anti-APE1 (Abcam, ab189474), anti-OGG1 antibody (Merck, MAB3560), anti-BG4 (Merck, MABE917) and RNA polymerase II (Pol II) (Abcam, ab76123). Approximately 0.5 million cells were processed, bound to concanaval in A-coated magnetic beads, and treated with the non-ionic detergent digitonin to permeabilize the cell membrane. Primary antibodies against APE1, OGG1, BG4 and Pol II were added and allowed to bind to their target proteins. Secondary antibodies were subsequently introduced to enhance binding specificity. Protein A-Tn5 (pA-Tn5) complexes were added to facilitate DNA cleavage near the bound proteins by Tn5 transposase, which is activated by magnesium ions. The resulting cleaved DNA was purified using magnetic beads, amplified by PCR, and sequenced on a NextSeq500 platform (Illumina) to achieve a minimum of 10^7 reads. The sequencing data were processed using Bowtie2 (v2.3.4.1) to align with the human reference genome GRCh38, with further analysis, including conversion of BAM to BED files using bedtools, visualization via IGV, removal of PCR duplicates using Picard tools, and peak calling using SEACR with stringent criteria. All sequencing experiments were conducted using three independent biological replicates per group. A comparative analysis based on CUT&Tag signal intensities at peak regions was performed to identify differentially bound regions (DPs) between two sample groups. Peaks from all samples were merged to generate a unified set of peak regions. For each peak in this union set, the normalized read coverage was calculated. Differential analysis between the two groups was then conducted using DESeq2, with significantly differential peaks defined as those satisfying the thresholds of |log2 fold change| ≥ 0.5 and p-value ≤ 0.05.

**Chromatin Immunoprecipitation (ChIP)-seq and ChIP-qPCR analysis.** Chip-seq was performed using a Simple Chip Plus Sonication Chip Kit (Thermo, 26157). Chromatin was harvested from 5×10^6 cells, crosslinked with 1% paraformaldehyde for 10 min at room temperature, followed by quenching with 0.125M Glycine. The cross-linked chromatin was lysed using SDS Lysis Buffer for 20 min and sonicated to yield fragments ranging from 200 to 1000 bp. ChIP was performed using specific APE1 (#ab189474, Abcam) antibodies with ChIP-Grade Protein G Magnetic Beads (9006). The chromatin complexes were then eluted from the beads and decrosslinked overnight in a 65 ℃ water bath. Nucleic acids were purified using spin columns to remove protein contaminants, and qPCR was conducted to amplify the targeted gene regions. Normal IgG (Millipore, 12-371, 3 µg per 1×10^6 cells) was used as a negative control. The ChIP-seq library was prepared on an Illumina Novaseq 6000 platform, generating 150bp paired-end reads. Quality control was performed using FastQC, followed by adapter trimming using Trimmomatic and Trim Galore software. The cleaned data were then aligned to the human reference genome GRCh38 using BWA to generate a BAM file. PCR duplicates were removed using Picard (GATK4) MarkDuplicates. Peak calling was conducted using MACS2, and the BAM file was converted to a BigWig file for visualization using deep Tools or IGV. ChIP DNA and 1% input DNA were subjected to SYBR Green-based real-time PCR. The data are represented as % input calculation [2^ (adjusted input − ChIP CT) × 100].

**Transposase-Accessible Chromatin (ATAC-seq).** ATAC-seq libraries were prepared using slight modifications to the Nextera Tn5 Transposase kit (Illumina). The experiment was performed with three biological replicates per group. Approximately 50,000 HCC827/HCC827ER cells were processed, beginning with triple PBS washes, followed by lysis using a cold buffer. The nuclei were then isolated via centrifugation and resuspended in reaction buffer containing the Tagment DNA enzyme. The transposition reaction was incubated at 37℃ with intermittent mixing. After the reaction, the DNA was purified and amplified using barcoding primers for 7 cycles, with size selection for fragments between 100-1000 bp. The libraries were quantified using Qubit and Agilent 4200 TapeStation and sequenced on an Illumina PE150 platform. Raw image data from sequencing were subjected to base calling to produce raw FASTQ sequences, which contained both nucleotide sequences and quality scores. Trimmomatic was used to clean the data of adapters and low-quality reads, and the volume of data before and after filtering was assessed. Clean data were aligned to GRCh38 reference genome using Bowtie2. The aligned reads were then further filtered to remove low-quality alignments (MAPQ < 30), PCR duplicates, and reads originating from organelles, resulting in the final set of high-quality reads for subsequent analysis, such as peak calling. Accessible chromatin peaks were called using MACS3 (v3.0.0) ‘BAMPE’ mode and q-value cutoff at 0.01. Differential accessibility analysis was done using the ‘Diffbind’ R package (v3.16).

**RNA sequencing（RNA-seq）.** NSCLC cells grown to near confluence were harvested for RNA sequencing. RNA was extracted and subjected to poly(A) selection before library preparation using a TruSeq kit (Illumina). Quality was assessed using a TapeStation (Agilent), and the libraries were quantified using a Qubit fluorometer. Sequencing was performed on an Illumina platform, achieving a minimum of 20 million reads per sample. The reads were processed through an established pipeline: alignment to the human reference genome (GRCh38) and transcriptome (Gencode.v29) was performed using STAR (v2.6.0c), and gene and isoform expressions were quantified using RSEM (v1.3.3). Here is the revised text, formatted as a single, coherent paragraph suitable for the Materials and Methods section of an international journal. Three biological replicates were used for each condition. Gene expression levels were quantified as read counts for each sample, and differential expression analysis was performed using the DESeq2 package (version 1.26.0). Genes with an adjusted p-value (padj) < 0.05 and an absolute log2 fold change (|log2FoldChange|) > 1 were considered significantly differentially expressed. Expression levels were normalized and reported as FPKM and TPM. To explore the functional implications of the differentially expressed genes, Gene Ontology (GO) and Kyoto Encyclopedia of Genes and Genomes (KEGG) pathway enrichment analyses were conducted using the clusterProfiler software (version 3.14.3). Enrichment significance was determined by p.adjust, the p-value corrected for multiple hypothesis testing (q-value), where values closer to zero indicate more significant enrichment. Additionally, Gene Set Enrichment Analysis (GSEA) was performed to identify coordinated expression changes within predefined gene sets, with significantly enriched sets defined by a false discovery rate (FDR q-val) < 0.25 and an absolute normalized enrichment score (|NES|) > 1. In cases where few significantly differentially expressed genes were identified, a relaxed threshold of p-value < 0.05 and |log2FoldChange| > 1 was applied.

**Whole-genome bisulfite sequencing (WGBS).** Whole-genome bisulfite sequencing was conducted on 1.0 × 10^5 HCC827 and HCC827 ER cells. DNA was extracted using lysis buffer and phenol-chloroform extraction, followed by precipitation with isopropanol and 70% ethanol. Purified DNA was dissolved in TE buffer, and 5 µg of genomic DNA was used for library preparation. DNA quality was assessed using the 260/280 ratio, with acceptable samples ranging from 1.8–2.0. DNA was then fragmented to approximately 250 bp using Covaris and bisulfite treatment was applied to convert unmethylated cytosine to thymidine. End repair and 3’-end adenylation were performed to prepare the DNA for adapter ligation. Methyl adapters were ligated and DNA fragments were PCR-enriched. The library concentration and insert size were determined using Qubit and Agilent 4200. High-throughput sequencing was performed using the Illumina PE150 platform. Data analysis included initial quality control using FastQC, followed by adapter and quality trimming using Trim-Galore and Trimmomatic. A second round of quality control ensured high-quality reads, which were then aligned to the human reference genome GRCh38 using the Bismark. Each group consisted of three biological replicates. Methylation analysis was performed using Bismark. Specifically, it was used for the alignment of bisulfite-converted reads, followed by the removal of PCR duplicates and the extraction of methylation calls for subsequent visualization. Differentially methylated regions (DMRs) were identified using the dmrseq R package. DMR detection was performed separately for CG, CHG, and CHH contexts. Candidate DMRs were filtered using stringent thresholds: Q value ≤ 0.05, number of CpGs ≥ 10, and absolute methylation difference ≥ 20%.

**Quantitative RT-qPCR.** Total cellular RNA was extracted from HCC827 and HCC827 ER cells using the TRIzol reagent (Invitrogen, CA, USA). RNA was then reverse-transcribed using HiScript Q RT SuperMix for qPCR (Vazyme, Jiangsu, China). Quantitative RT-PCR was performed using the SYBR Green PCR Master Mix (Vazyme) on a CFX96 Real-Time System (Bio-Rad, USA). We measured the expression levels of MAP3K4, ANKS1B, CLDN2, ESRP1, USP25, and KRT15. The mRNA expression was normalized to that of GAPDH by qPCR using Power SYBR Green. The results were analyzed and quantified using the 2^(− ΔΔCT) method. The primers used in these assays were synthesized by Sangon Biotech (Shanghai, China): ESRP1 (forward: 5'-CAGAGGCACAAACATCACAT-3'; reverse: 5'-AGAAACTGGGCTACCTCATTGG-3'), USP25 (forward: 5'-ATGCCTATGAGGAAACTTGA-3'; reverse: 5'-GGTAGACCTATTTGCCCACT-3'), CLDN2 (forward: 5'-CCTTTATCACCTCAGCCCGT-3'; reverse: 5'-GCTACCGCCACTCTGTCTTT-3'), MAP3K4 (forward: 5'-CTCCTCGACCACGCTTTTGT-3'; reverse: 5'-CCTGCGCTGCAGTCTTTATG-3'), ANKS1B (forward: 5'-GCCCTACACTGTGCAGCTCAATA-3'; reverse: 5'-GGGTCAGTGAGCTCTTCTAGGAG-3'), SEC61G (forward: 5'-GCAGTTTGTTGAGCCAAGTCG-3'; reverse: 5'-CCAGCCGAATGGAGTCCTT-3'), KRT15 (forward: 5'-AGAAATCTGAATTCCTATTGCAGGAGA-3'; reverse: 5'-CCCTGAAAGCTTAGACCGAGGGACCCT-3'), GAPDH (forward: 5'-CATGTGGGCCATGAGGTCCACCAC-3'; reverse: 5'-GGGAAGCTCACTGGCATGGCCTTCC-3')**.**

**Electrophoretic Mobility Shift Assay (EMSA).** EMSA was performed using a LightShift Chemiluminescent EMSA Kit (#20148, Thermo, USA). Biotin-labeled and non-biotin-competitive G4 oligonucleotide probes, along with their mutant versions and complementary strands, were used in the assay. Synthetic double-stranded DNA templates were generated by incubating 10 µM of each strand with annealing buffer at a final volume of 100 µL for 5 min at 95° C, followed by slow cooling from 95° C to 37 °C. G4 DNA formation was induced by the addition of 100 mM KCl to the annealing reaction. Oligos were stored at -20° C. Purified APE1 protein (#Ag0251, Proteintech, China) was incubated with these probes in a 20 µL reaction volume using the Epstein-Barr nuclear antigen (EBNA) system. After a 20-minute incubation at room temperature, the reaction mixtures were electrophoresed on a 6% polyacrylamide gel in 0.5×TBE buffer at 100 V. The probes were then transferred onto a nylon membrane (#FFN10, Beyotime, China) at 380 mA for 30 min. After transfer, the DNA on the membrane was crosslinked using a 253.7 nm UV lamp for 30 min. Biotin-labeled DNA was detected using chemiluminescence. The oligonucleotide probes used in these assays were synthesized by Sangon Biotech (Shanghai, China).

**Flow cytometry.** Cells were resuspended in 100 μl of PBS containing 1% FBS and incubated at 4°C for 30 min with PE anti-human CD133 antibody (#397903, Biolegend). After two PBS washes, the labeled cells were analyzed using a Cytoflex flow cytometer (Beckman Coulter, USA).

**Transwell.** 2×10^4 cells were suspended in 200 μl of FBS-free conditioned medium and placed in the upper chamber of a transwell insert pre-coated with Matrigel. The lower chamber was filled with 600 μl of RPMI 1640 medium supplemented with 10% FBS. After a 24-hour incubation, the cells were fixed with 4% paraformaldehyde for 25 min at room temperature and stained with crystal violet (C0121; Beyotime, China) for 15 min. Cell migration was quantified by counting the cells in five randomly selected fields under an EVOS FL auto microscope (Thermo, USA).

**TRITC-Phalloidin stain.** Cells were seeded on glass-bottom dishes (#801002, NEST, China) a day before the experiment. The cells were fixed with 4% paraformaldehyde for 10 min at room temperature, permeabilized with 0.05% Triton X-100 for 5 min, and blocked with 1% BSA in PBS for 20 min. The cells were then stained with TRITC-phalloidin (#BB441424; Bestbio, China) for 1 h in the dark at room temperature and counterstained with DAPI (100 ng/μl) for 5 min. The stained cells were examined using a laser scanning confocal microscope (Zeiss, Germany).

**Sphere formation assay.** For the sphere formation assay, 1000 cells were seeded in a six-well ultralow attachment plate (Corning Glass) and cultured in serum-free DMEM/F12 (Gibco, USA, #111330032) supplemented with N2 (Invitrogen), 20 ng/mL human recombinant epidermal growth factor (hEGF, Peprotech, USA, #315–09), and 20 ng/mL human recombinant basic fibroblast growth factor (hFGF, Novoprotein, #C046). After incubation for 10–14 days, the number of spheres was counted by microscopy and statistically analyzed.

**Western blot.** Whole cell protein extracts were collected from RIPA lysis buffer (50 mmol/L Tris, pH 8.0, 150 mmol/L NaCl, 5 mmol/L MgCl2, 1% Triton X-100, 0.1% SDS), and the protein concentration was detected using the BCA assay. Equal amounts of proteins were subjected to SDS-PAGE and transferred onto nitrocellulose membranes. After blocking with 5% nonfat dry milk for 1 h, the transblot sheets were incubated with appropriate primary antibodies overnight at 4℃, followed by corresponding secondary antibody incubation. After washing three times with PBS, the bands were visualized using an Odyssey Infrared Imaging System (Li-Cor Biosciences, Lincoln, NE). Relative protein expression levels were calculated from the gray intensity of the band. The primary antibodies used for western blot analysis were anti-APE1 (1:1000; Abcam; ab189474), Anti-OGG1 (1:1000; Abcam; ab233214), anti-E-cadherin (1:1000; Abcam; ab314063), anti-N-cadherin (1:1000; Abcam; ab76011), anti-vimentin (1:1000; Abcam; ab92547), Anti-CD44 (1:1000; Abcam; ab254530), Anti-CD133 (1:1000; Abcam; ab278053), Anti-OCT4 (1:10000; Abcam; ab200834), Anti-ALDH1 (1:10000; Abcam; ab200834), anti-alpha-tubulin (1:5000, Abcam, ab7291), and GAPDH (1:5000, Abcam, ab8245). The secondary antibodies used for the western blot studies were goat anti-rabbit secondary antibody (1:1000, Beyotime, A0208) or goat antimouse secondary antibody (1:1000, Beyotime, A0216).

**Immunofluorescence.** The cells were washed thrice with PBS and fixed in methanol for 2 h. Next, they were permeabilized with 0.02% Triton X-100 for 15 min and blocked with 5% goat serum at room temperature for 1 h. The cells were then incubated with the respective primary antibodies overnight at 4℃. After washing, the coverslips were incubated with secondary antibodies for 1 h at 37℃. Finally, the cells were stained with DAPI and imaged using a laser scanning confocal microscope (Zeiss, Oberkochen, Germany). The primary antibodies used for immunofluorescence studies were mouse monoclonal anti-APE1 (1:5000; Abcam; ab194), Anti-G4-1H6 (1:50; Sigma-Aldrich; MABE1126) and Anti-H3K4Me3 (1:250; Cell Signaling Technology; #9751S). The secondary antibodies used for immunofluorescence studies were Alexa Fluor 488 antimouse IgG (Life Technologies, 1:500) or Alexa Fluor 594 anti-rabbit IgG (Life Technologies; 1:500).

**Immunohistochemistry.** Formalin-fixed and paraffin-embedded (FFPE) specimen sections were obtained from the Department of Pathology, Daping Hospital, for IHC analysis. After dewaxing, heat-induced epitope retrieval (HIER) was performed using EDTA (pH 8.0) (MXB Kit5030, China). Following a 10-minute incubation at room temperature with endogenous peroxidase blocker (#SP KIT-A3, MXB, China), the sections were incubated with APE1/OGG1 antibodies overnight at 4℃. The next day, the sections were incubated with HRP-conjugated secondary antibodies at 37℃ for 30 min and stained with 3,3’-diaminobenzidine (DAB). The nuclei were counterstained with hematoxylin, and the primary antibodies used for immunofluorescence studies were anti-APE1 (1:2000; Abcam; ab189474) and Anti-OGG1 (10 µg/ml; Abcam; ab233214).

A modified H-score system was applied, incorporating both staining intensity and the percentage of positive cells. Staining intensity was graded as 0 (no staining), 1+ (faint yellow, weak), 2+ (brown-yellow, moderate), or 3+ (dark brown, strong). The percentage of positive cells was estimated in 5% increments. The total H-score was calculated as the sum of the products of the intensity scores and their corresponding percentages (Σ [intensity score × percentage of cells]). To ensure reproducibility, stringent quality control measures were implemented. Each staining batch included parallel positive controls (high-expression tissue microarrays) and negative controls (slides omitting primary antibodies). Optical density calibration was performed using ImagePro Plus 6.0 software (Media Cybernetics) with a standardized white balance threshold of 230-255. Inter-rater discrepancies (>15% variance in scores) were resolved through consensus review using a dual-head microscope (Olympus BX53). Final scores represent the mean of both pathologists' assessments.

**Xenograft studies.** All BALB/c nude mice were given free access to tap water and housed in a room with a 12:12-hour light–dark cycle and a temperature of 22 ℃ throughout the duration of the experiments at the Animal Centre of Daping Hospital, Third Military Medical University. （1） Thirty 4-week-old (BALB/c) male nude mice were randomly divided into two groups (10 mice in HCC4006 ctrl group, 20mice in HCC4006 APE1 OE group): HCC4006 ctrl and APE1 OE. HCC4006 cells with stable overexpression of APE1 (HCC4006 APE1 OE) and control cells (HCC4006 ctrl) (5 ×10^6^/100μL cells per mouse) were subcutaneously injected into the right flank of nude mice. When the average volume of nude mice was about 80-120mm^3^, HCC4006 ctrl group was divided into two groups: 4006 ctrl+Normal Saline(NS) and 4006 ctrl+Erlotinib. HCC4006 APE1 OE group was divided into four groups: 4006 APE1 OE+NS, 4006 APE1 OE+Erlotinib, 4006 APE1 OE+Erlotinib+NS and 4006 APE1 OE+Erlotinib+MX. Next, 4006 ctrl+Erlotinib, APE1 OE+Erlotinib, 4006 APE1 OE+erlotinib+NS and 4006 APE1 OE+Erlotinib+MX groups were fed with Erlotinib 10 mg/kg, 4006 APE1 OE+Erlotinib+NS and 4006 APE1 OE+Erlotinib+MX followed by intraperitoneal injections of NS (as control ) and MX (10 mg/kg) every two days. (2) 3×10^6 H4006ER cells were subcutaneously injected into mice at 4 weeks of age with an average body weight of 20 g. After four weeks, when palpable tumors were visible, mice were divided into three groups (n = 7 in each group). Control groups were fed with Erlotinib 10 mg/kg and normal saline. The other two groups were fed with Erlotinib 10 mg/kg, followed by intraperitoneal injections of CRT0044876 (5 mg/kg, specifically inhibits the AP endonuclease activity of APE1) or SU-0268 (10 mg/kg, specifically inhibits the activity of OGG1) three times a week for 2 weeks. CRT0044876 and SU-0268 were purchased from MCE (China), and MX was purchased from Sigma-Aldrich (USA). Xenografts were evaluated by visual inspection every two days after cell injection. When the mice reached 10 weeks of age, they were euthanized under pentobarbital anesthesia (50 mg/kg body weight, intraperitoneally) for the isolation and harvesting of tumor tissues (tumor volume not exceeding 2,000 mm^3). (3) Sixteen 4-week-old (BALB/c) male nude mice were randomly divided into two groups (8 mice in HCC4006ER NC group, 8 mice in HCC4006ER APE1 KD group), 3 ×10^6^/100μL cells per mouse were subcutaneously injected into the right flank of nude mice. When the average volume of nude mice was about 80-120mm^3^, each group was divided into two groups: HCC4006ER NC +NS and HCC4006ER NC +Erlotinib; HCC4006ER APE1 KD +NS and HCC4006ER NC +Erlotinib. (4) in vivo studies were conducted using a pre-established HCC4006 xenograft model with acquired resistance to erlotinib (HCC4006 ER). To validate the stability of the resistant phenotype under continuous drug pressure in vivo, tumor-bearing mice were treated with erlotinib once tumors reached an average volume of 150 mm³. Treatment was continued for 6 days. Following this confirmation, a late-intervention strategy was initiated, wherein the erlotinib-resistant tumors were treated with an APE1 inhibitor or an OGG1 inhibitor as monotherapy.

The study was approved by the Research Council and Animal Care and Use Committee of the Daping Hospital, Third Military Medical University. All experiments conformed to the guidelines for the ethical use of animals, with efforts made to minimize animal suffering and reduce the number of animals used. The animals were treated and cared for in accordance with the National Research Council’s Guide for the Care and Use of Laboratory Animals.

**Patient tissue samples**

Samples from cancer patients were collected from the Cancer Center, Daping Hospital, and Army Medical Center of PLA. 13 NSCLC patient paired tissue samples before and after the development of ER were collected and stored at −80 °C. This study was approved by the Ethics Committee of the Cancer Center, Daping Hospital, and Army Medical Center of PLA. Informed consent was obtained from all patients.

**Statistical analysis**

Statistical analysis of the experimental results was performed using GraphPad Prism 8. An unpaired two-tailed Student’s t-test was applied, and results with a 95% confidence interval (P < 0.05) were considered statistically significant. All P-values <0.05 were considered significant and were denoted as follows: NS, P ≥ 0.05; *P = 0.01 to < 0.05; **P = 0.001 to < 0.01; ***P = 0.0001 to < 0.001; ****P ≤ 0.0001. Unless otherwise specified, bar plots or center lines in box plots represent mean values, and error bars represent standard deviations. Statistical data are expressed as mean ± SE. For data with n≥6, normality was determined using the Shapiro–Wilk test before statistical analysis. For normally distributed data, differences between two groups were analyzed using an unpaired Student’s t-test. For multiple group comparisons, one-way analysis of variance (ANOVA) with Bonferroni’s post-hoc analysis was used. For non-normally distributed data with n<6, differences between the two groups were analyzed using the nonparametric Mann–Whitney U test.
