# Supplementary material for: DNA base lesion-containing G-quadruplex mediates transcriptome reprogramming in EGFR-TKI resistance of non-small cell lung cancer
Source: J Exp Clin Cancer Res. 2026 Apr 14;45:122. doi: 10.1186/s13046-026-03702-w (PMC13185293; doi:10.1186/s13046-026-03702-w)
Supplement: Supplementary file 3 — Supplementary Material 3. [file 13046_2026_3702_MOESM3_ESM.docx]

**Figure legends**

**Fig.1│Oxidative DNA damage accumulation and BER activation in EGFR-TKI resistant NSCLC cells. a,** Genomic distribution of AP sites in HCC827 and HCC827ER cells. The concentric rings (outer to inner) represent: chromosomes, HCC827-specific AP sites, gene-mapped AP site counts for HCC827, HCC827ER-specific AP sites, and gene-mapped AP site counts for HCC827ER. The outermost ring labels genes with ≥30 AP sites (color-coded: red, HCC827ER; blue, HCC827; green, shared). **b,** GSEA pathway analysis highlighting cancer-related signaling pathways associated with altered DNA repair in HCC827ER cells compared with parental cells. **c,** Differential expression analysis of BER pathway genes in HCC827ER vs. parental cells. **d,** Representative images of IHC analysis of the expression of OGG1 and APE1 in 13 pairs of human NSCLC tissue samples before and after resistance to first- and second-generation EGFR-TKI treatment (Scale bar, 100 µm). **e,** Elevated APE1 expression significantly correlates with reduced overall survival in the TCGA-LUAD cohort (log-rank p=0.031). **f,** APE1 and OGG1 were significantly upregulated in NSCLC tissues compared to normal controls by analyzing TCGA-derived clinical samples. **g,** Erlotinib dose-response curves for control and APE1 or OGG1 OE in parental cell lines and control and APE1 or OGG1 KD in ER counterparts. IC₅₀ values of sensitive and resistant cell lines. n=3 experimental replicates, mean ± SEM. Significance was calculated using a paired t test. *p < 0.05.

**Fig.2│OGG1/APE1 driven transcriptional reprogramming established EMT- and stem-like plasticity in EGFR-TKI resistant NSCLC cells. a,** Volcano plots representing differentially expressed genes (DEGs) in HCC827 and HCC827ER cells. Upregulated genes (red), downregulated genes (green). **b,** Gene Ontology (GO) Enrichment analysis reflected up and down regulated pathway terms associated with DEGs in HCC827 and HCC827ER cell lines. The dot color represented Normalized Enrichment Score (NES), and the dot size indicated the number of overlapping genes per pathway. **c,** GSEA pathway analysis highlighted cancer-related signaling pathways corresponding to lineage-specific signatures altered in HCC827ER cells compared to parental cells. NES：Normalized Enrichment Score. **d,** GSEA enrichment analysis revealed a strong correlation between DEGs and EMT- and stem-like signatures. **e,** Differential expression analysis of EMT, migration, focal adhesion, and cell-stem response genes in HCC827ER cells compared to parental cells. **f,** Differential expression analysis of EMT, migration, focal adhesion and cell stem response genes in HCC827ER APE1 KD cells compared to HCC827ER cells. **g,** Differential OGG1 and APE1 binding sites in SNAI1/ZEB1/ZEB2 region in HCC827 cells treated with 0.5 μm erlotinib for 0, 2, 4 and 6 days. **h,** Differential OGG1 and APE1 binding sites promoter regions in HCC827 versus HCC827ER cells.

**Fig.3│OGG1/APE1 mediate EMT- and stem-like plasticity to drive EGFR-TKI Resistance in NSCLC. a,** Western blot analysis of EMT markers and APE1/OGG1 in two panels of sensitive and resistant cells. **b,** Phalloidin immunofluorescence showing enhanced actin stress fiber formation in HCC827 cells with OGG1 or APE1 overexpression (OE), and reduced stress fiber levels in HCC827ER cells with OGG1 or APE1 knockdown (KD). Control (Ctrl); Overexpressed (OE); Knockdown (KD). **c,** Western blot analysis of EMT and stemness markers in a panel of sensitive and resistant cells. **d,** Representative phase‑contrast images (right) and quantitative analysis (left) of tumorsphere formation in HCC827 (APE1/OGG1 OE) and HCC827ER (APE1/OGG1 KD) cells. Results are presented as mean ± SEM; n = 3; *P < 0.05, **P < 0.01, ***P < 0.001, independent t test. **e,** Surface expression of CD133 (cancer stem cell marker) in HCC827 (APE1/OGG1 OE) and HCC827ER (APE1/OGG1 KD) cells. Results are presented as mean ± SEM; n = 3; *P < 0.05, **P < 0.01, ***P < 0.001, independent t test. **f,** Representative illustration of human NSCLC tissue stained by multiplex IHC before and after resistance to first- and second-generation EGFR-TKI treatments. Scale bar, 50 µm. DAPI (blue), APE1 (cyan), E-cadherin (yellow), vimentin (red), CD44 (green), and CD133 (orange) staining.

**Fig.4│Redistribution of genome-wide OGG1/APE1 binding sites and G4 structures in EMT- and stem-like related genes. a,** Venn diagram showing overlapping APE1 binding sites (APE1 peaks) between HCC827 and HCC827ER cells, with differential distribution across promoters, gene bodies, and intergenic regions. **b,** Venn diagram of overlapping OGG1 binding sites (OGG1 peaks) between HCC827 and HCC827ER cells, including genomic distribution analysis. **c,** Venn diagram of overlapping G-quadruplex (G4) structures (G4 peaks) between HCC827 and HCC827ER cells, with genomic distribution across promoter, gene body, and intergenic regions. **d,** Whole-genome circular plot displaying co-distribution of APE1, OGG1 binding sites, and G4 structures in HCC827 and HCC827ER cells. Genomic windows (100-kb) were used to calculate peak density. Tracks (from outer to inner): chromosomes; APE1 binding (HCC827); APE1 binding (HCC827ER); OGG1 binding (HCC827); OGG1 binding (HCC827ER); G4 structures (HCC827); G4 structures (HCC827ER). Color gradient (light red → dark red) indicates peak abundance (darker = higher density). **e,** Genomic distribution of 8-oxo-G/AP sites and G4 structures in ER resistant cells and their correlation with downstream gene expression. **f,** qRT-PCR analysis of EMT/stemness gene expression following OGG1/APE1 knockdown in resistant cells. Results are presented as mean ± SEM; n = 3; *P < 0.05, **P < 0.01, ***P < 0.001, independent t test. **g,** The shaded region highlights the overlap of OGG1, APE1, and BG4 binding in the MAP3K4 promoter region (HCC827 vs. HCC827ER), showing elevated AP site density in HCC827ER cells. **h,** The shaded region highlights the overlap of OGG1, APE1, and BG4 binding in the ESRP1 promoter region (HCC827 vs. HCC827ER), showing reduced AP site density in HCC827ER cells.

**Fig.5│APE1 exhibits stronger affinity towards promoter G4 regions than non-G4 regions in EMT- and stem-like related genes. a,** Occurrence of APE1 binding sites in G4-containing promoters of DEGs (PQS score ≥20 considered significant). **b,** Schematic highlighting G4-forming regions (shaded) and non-G4 control regions (white box) in promoters of EMT/stemness genes (MAP3K4, CLDN2, ANKS1B, ESRP1, USP25, KRT15). G4 regions were identified via QGRS Mapper (PQS score ≥20). **c,** Representative genome browser views of EMT and stemness-related gene promoter regions (MAP3K4, CLDN2, ANKS1B, ESRP1, USP25, and KRT15) showing OGG1/APE1 binding and G4 profiles in HCC827 and HCC827ER cells. **d,** ChIP-qPCR validation of APE1 enrichment at G4 vs. non-G4 regions in EMT and stemness-related genes (MAP3K4, CLDN2, ANKS1B, ESRP1, USP25, and KRT15) in HCC827 and HCC827ER cells. Primers were utilized to amplify the G4 region and a non-G4 control region. The P-values were determined by an unpaired Student’s t-test (****P < 0.0001, ***P < 0.001, **P < 0.01, *P < 0.05). Error bars denote ± SEM. Three independent experiments were performed in triplicates. **e and f,** EMSA confirming APE1 binding to G4-forming oligonucleotides (derived from ANKS1B and ESRP1 promoters) and non-G4 controls. Biotinylated G4 oligonucleotides and mutant control sequences were constructed.

**Fig.6│APE1 modulates transcription of EMT- and stem-like related genes by stablizing G4 structures in cells.** **a,** Differential gene expression in HCC827 vs. HCC827ER cells categorized by G4 and ATAC signal presence, analyzed relative to ATAC-/G4- baseline. The presence (+) or absence (−) of G4s or ATAC signals are reported. Gene expression distribution of the differentially expressed genes in HCC827 vs. HCC827ER cells was evaluated by two-sided t test in comparison to the G4:ATAC -/- condition (CI 95%, ***p value < 0.001). **b,** Expression distribution of DEGs grouped in HCC827 vs. HCC827ER cells according to the presence of G4 signals (BG4-CUT&TAG Seq) in their gene region. Gene expression is reported as log_10_FPKM. **c,** APE1 KD HCC827 cells were transfected with APE1 WT plasmid, redox-defective C65/93S, or repair-defective H309A plasmid for 48 hours. Cells were then immunostained with α-1H6 and APE1, counterstained with DAPI, and visualized by confocal microscopy (Magnification: 63×; Scale bars: 0.3 inches). **d,** Erlotinib dose-response curves for APE1 KD HCC827 cells transfected with APE1 WT plasmid, redox-defective C65/93S, or repair-defective H309A plasmid. n = 3 experimental replicates, mean ± SEM *P < 0.05, **P < 0.01, ***P < 0.001. **e,** Representative fields of view showing G4 foci formation detected by confocal microscopy in control, APE1 OE, APE1 OE + PDS (5 µM, 2 hours), and APE1 OE + MX (50 µM, 2 hours) treated HCC827 cells (Magnification: 63×; Scale bars: 0.3 inches). **f,** qRT-PCR assays were performed in control, APE1 OE, APE1 OE + PDS (5 µM, 2 hours), and APE1 OE + MX (50 µM, 2 hours) treated HCC827 cells, and relative gene expression (normalized to GAPDH) of EMT and stemness-related genes (MAP3K4, CLDN2, ANKS1B, ESRP1, USP25, and KRT15) was calculated. The P-values were determined by an unpaired Student’s t test (****P < 0.0001, ***P < 0.001, **P < 0.01, *P < 0.05). Error bars denote ± SEM. Three independent experiments were performed in triplicates. **g,** Actin stress fiber quantification (phalloidin staining, 63×; scale bar = 0.3 mm) in control, APE1 OE, APE1 OE + PDS (5 µM, 2 h), and APE1 OE + MX (50 µM, 2 h) cells.. **h,** The expression of the classical tumor stemness marker CD133 on the cell surface in control, APE1 OE, APE1 OE + PDS (5 µM, 2 hours), and APE1 OE + MX (50 µM, 2 hours) treated HCC827 cells. **i,** Protein expression of EMT and stemness markers was analyzed by western blot in control, APE1 OE, APE1 OE + PDS (5 µM, 2 hours), and APE1 OE + MX (50 µM, 2 hours) treated HCC827 cells.

**Fig.7│G4 structures are found within hypomethylated CGIs in EMT- and stem-like related genes. a,** Box and whisker plots showing the average methylation for BG4 peaks and CGIs in HCC827 cells. **b,** Methylation levels at BG4 peaks and CGIs across CpG density bins in HCC827 cells. **c,** Methylation levels at CGIs with or without a BG4 peak at different CpG densities in HCC827 cells. **d,** Methylation levels at BG4 peaks and CGIs in HCC827ER cells. **e,** Methylation levels at BG4 peaks and CGIs across CpG density bins in HCC827 cells. **f,** Methylation levels at CGIs with or without a BG4 peak at different CpG densities in HCC827ER cells. **g,** Comparison of CGI methylation levels between HCC827 and HCC827ER cells. **h,** An IGV screenshot illustrating the co-incidence of BG4 peaks with hypomethylated promoter CGIs for EMT and stemness-related genes (MAP3K4, CLDN2, ANKS1B, ESRP1, USP25, and KRT15) in HCC827 and HCC827ER cells. Shown are normalized signals. **i,** qRT-PCR analysis of EMT and stemness-related genes expression following T-5224 (5 µM, 24 h) treatment in parental (HCC827) and resistant (HCC827ER) cells. Results are presented as mean ± SEM; n = 3; *P < 0.05, **P < 0.01, ***P < 0.001, independent t test.

**Fig.8│Combined with BER inhibitors sensitizes ER cells to EGFR-TKI and suppresses xenograft tumor growth in vivo. a,** Resected tumors after completion of treatment. Thirty 4-week-old male BALB/ude mice were randomized into two groups: HCC4006 control (n=10) and HCC4006 APE1 OE (n=20). Mice received subcutaneous injections of 5×10⁶ cells (100 μL) in the right flank. At tumor volumes of ~100 mm³, the control group was divided into NS and erlotinib (10 mg/kg) subgroups, while the APE1 OE group was allocated to four treatment arms: NS, Erlotinib, Erlotinib + NS, and Erlotinib + MX (10 mg/kg, i.p., every 2 days). **b,** Tumor volume was measured on indicated days, and the tumor growth curve was plotted. **c,** Tumor weight of resected tumors after completion of treatment. **d,** Resected tumors after completion of treatment. 3×10⁶ HCC4006ER cells were subcutaneously injected into 4-week-old mice (20 g average weight). After 4 weeks, tumor-bearing mice were randomized into three groups (n=7/group): (1) Erlotinib (10 mg/kg) + NS; (2) Erlotinib + CRT0044876 (APE1 endonuclease inhibitor, 5 mg/kg, i.p., 3×/week); (3) Erlotinib + SU-0268 (OGG1 inhibitor, 10 mg/kg, i.p., 3×/week). Tumor growth was monitored biweekly until euthanasia at 10 weeks (pentobarbital, 50 mg/kg, i.p.), with tumor collection (<2,000 mm³). CRT0044876 and SU-0268 were from MCE (China); MX from Sigma-Aldrich (USA). **e,** Representative IHC images of OGG1 and APE1 expression in resected tumors (Scale bar, 50 µm). **f,** Tumor volume was measured on indicated days, and the tumor growth curve was plotted. **g,** Tumor weight of resected tumors after completion of treatment. **h,** Representative IHC images of Vimentin, CD44, CLDN2, and ESRP1 expression in resected tumors (Scale bar, 50 µm). **i,** Resected tumors after completion of treatment. 3×10⁶ H4006ER cells were subcutaneously injected into 4-week-old mice (20 g average weight). After 4 weeks, tumor-bearing mice were randomized into six groups (n=4/group): (1) Control: NS; (2) Erlotinib (10 mg/kg); (3) Erlotinib (10 mg/kg, continued treatment for 6 days) followed by CRT0044876 (APE1 endonuclease inhibitor, 5 mg/kg, i.p., 3×/week); (4) Erlotinib (10 mg/kg, continued treatment for 6 days) followed by SU-0268 (OGG1 inhibitor, 10 mg/kg, i.p., 3×/week); (5) Erlotinib (10 mg/kg, continued treatment for 6 days) followed by CRT0044876 (APE1 endonuclease inhibitor, 5 mg/kg, i.p., 3×/week) combined with erlotinib (10 mg/kg); (6) Erlotinib (10 mg/kg, continued treatment for 6 days) followed by SU-0268 (OGG1 inhibitor, 10 mg/kg, i.p., 3×/week) combined with erlotinib (10 mg/kg). Endpoint: 10 weeks or <2,000 mm³. **j,** Tumor growth curves for panel i model.

**Fig.9│Proposed schematic representation.** Proposed schematic representation of endogenous oxidative DNA damage orchestrates APE1-mediated G4 stabilization at promoter regions of EMT and stemness-related genes, driving transcriptome reprogramming through epigenetic regulation. Notably, the enrichment of G4 structures within hypomethylated CGIs implies a functional relationship between secondary DNA structures and DNA methylation dynamics, which may play a regulatory role in gene expression control. Specifically, APE1 binding to PQS facilitates G4 structure formation, which in turn serves as an epigenetic scaffold for sustained expression of EMT and stem-like plasticity genes. This BER-mediated epigenetic remodeling represents a critical molecular determinant of acquired TKI resistance, highlighting the functional interplay between DNA damage response, chromatin architecture, and lineage plasticity in therapeutic resistance.

**Extended Data Fig.1│Chromatin accessibility and gene regulatory underpinnings of erlotinib resistant cell lines. a,** Erlotinib-resistant cell lines (HCC827ER and HCC4006ER) were established by low-concentration-gradient induction of erlotinib. HCC827ER and HCC4006ER displayed morphological changes characterized by elongated and irregular shapes compared to the parental cells. IC₅₀ values for sensitive and resistant cell lines. **b,** Erlotinib dose-response curves for parental cell lines and their TKI-resistant counterparts. n=3 experimental replicates, mean ± SEM. **c,** Sanger sequencing identified EGFR mutation status in HCC827ER and HCC4006ER. No EGFR T790M mutations were detected in the genomes of HCC827ER and HCC4006ER cell subpopulations. **d,** KEGG analysis reflected up- and down-regulated pathway terms corresponding to DEGs in HCC827 and HCC827ER cell lines. **e,** Peak annotation was performed to assess the distribution of DARs across different genomic feature types in HCC827 and HCC827ER cell lines. **f,** Quadrant analysis of genes with coordinated changes in chromatin accessibility and expression: (I) increased accessibility with upregulated expression, (II) increased accessibility with downregulated expression, (III) decreased accessibility with upregulated expression, and (IV) decreased accessibility with downregulated expression. **g,** GO functional annotation and enrichment analysis of gene sets in (I) and (IV) group.

**Extended Data Fig.2│OGG1/APE1 alternative transcriptional programs underlying the shift to EMT- and stem-like plasticity. a，**Volcano plots representing DEGs in HCC827ER and HCC827ER APE1 KD cells. **b,** GSEA pathway analysis highlighting cancer-related signaling and lineage-specific signatures altered in HCC827ER APE1 KD cells compared to HCC827ER cells. NES, normalized enrichment score. **c,** Volcano plots represent DEGs in HCC827ER and HCC827ER OGG1 KD cells. **d,** GSEA pathway analysis highlighting cancer-related signaling and lineage-specific signatures altered in HCC827ER OGG1 KD cells compared to HCC827ER cells. NES, normalized enrichment score. **e,** Differential expression analysis of EMT, migration, focal adhesion and LUAD response gene expression in HCC827ER OGG1 KD cells vs. HCC827ER cells. *P < 0.05, **P < 0.01, ***P < 0.001, independent t test.

**Extended Data Fig.3│OGG1/APE1 induce EMT- and stem-like plasticity in EGFR-TKI resistant NSCLC cells. a,** Representative transwell invasion assays comparing control vs. APE1 or OGG1 OE in parental cell lines, and control vs. APE1 or OGG1 KD in ER counterparts. n = 3 experimental replicates, mean ± SEM, *P < 0.05, **P < 0.01, ***P < 0.001. **b,** High-resolution, multiplex imaging analysis of APE1 and EMT- and stem-like markers in tumor regions. *P < 0.05, **P < 0.01, ***P < 0.001, Wilcoxon test.

**Extended Data Fig.4│OGG1/APE1 mediate EMT- and stem-like plasticity to drive EGFR-TKI Resistance in NSCLC.** **a,** Western blot analysis for EMT markers (N‑cadherin, and vimentin) in HCC827/HCC4006 cells upon OGG1 or APE1 OE and in HCC827ER/HCC4006ER cells upon OGG1 or APE1 KD. **b,** Phalloidin immunofluorescence showing enhanced actin stress fiber formation in HCC4006 cells with OGG1 or APE1 OE, and reduced stress fiber levels in HCC4006ER cells with OGG1 or APE1 KD. **c,** Western blot analysis of APE1 and stemness markers in a panel of sensitive and resistant cells. **d,** Phase images (Left) and quantification data (Right) showed tumorsphere formation in APE1/OGG1 OE in HCC4006 cells and KD in HCC4006ER cells. Results are presented as mean ± SEM; n = 3; *P < 0.05, **P < 0.01, ***P < 0.001, independent t test. **e,** Surface expression of CD133 (cancer stem cell marker) in HCC4006 (APE1/OGG1 OE) and HCC4006ER (APE1/OGG1 KD) cells. Results are presented as mean ± SEM; n = 3; *P < 0.05, **P < 0.01, ***P < 0.001, independent t test.

**Extended Data Fig.5│Redistribution of genome-wide OGG1/APE1 binding sites and G4 structures in EMT- and stem-like related genes. a,** Differential APE1/OGG1/BG4 binding sites between HCC827 and HCC827ER, represented by the number of differential peaks (DP) on the y-axis and the fold change in DP on the x-axis (log_2_Fold Change). Red indicates Gain DP and blue represents Loss DP. **b,** Enrichment of APE1/OGG1/BG4 CUT&TAG-Seq signals within ± 2kb of TSS in HCC827 and HCC827ER cells. **c,** Annotated genes screened by cross-comparison were significantly enriched in the regulation of the cellular stress response, as anticipated. **d,** qRT-PCR analysis revealed that OGG1/APE1 KD regulates the expression of EMT- and stemness-related genes in resistant cell lines. Results are presented as the mean ± SEM; n = 3; *P < 0.05, **P < 0.01, ***P < 0.001, independent t test. **e,** Representative images of IHC analysis of the expression of MAP3K4, CLDN2 and ESRP1 in 13 pairs of human NSCLC tissue samples before and after resistance to first- and second-generation EGFR-TKI treatment (Scale bar, 50 µm).

**Extended Data Fig.6│G4s mediated EMT- and stem-like related genes and detection of G4s foci upon transcription perturbation treatments. a,** Metaprofiles of the relative enrichment of BG4 with respect to ± 3kb of TSS in HCC827 and HCC827ER cells. **b,** Expression distribution of DEGs in HCC827 versus HCC827ER cells stratified by G4 structure presence (+) or absence (-) and chromatin accessibility (ATAC signals). Bars represent log_2_(FPKM) expression values, with statistical significance (two-sided t-test) calculated relative to G4-/ATAC- conditions. **c,** Co-occurrence of G4 structures and chromatin accessibility in EMT and stemness-related genes (MAP3K4 and ESRP1). **d,** Representative fields of G4 foci formation detected by immunofluorescence in control non-treated (Ctrl) and entinostat (2 µM and 4 µM)-treated HCC827 and HCC4006 cells. Nuclear staining (blue), BG4 (green), and merged channels have been previously reported. Scale bars = 0.3 inches. The fields shown belong to one of three independent biological replicates. **e,** Representative fields of G4 foci formation detected by immunofluorescence in control (untreated) and actinomycin D-treated (0.5 μM and 1 μM) HCC827 and HCC4006 cells. Nuclear staining (blue), BG4 (green), and merged channels have been previously reported. Scale bars = 0.3 inches. The fields shown belong to one of three independent biological replicates. **f,** Differential RNA polymerase II (Pol II) and BG4 binding at EMT- and stemness-related genes in HCC827ER versus HCC827ER APE1 KD cells. **g,** Western blot analysis of Pol II C-terminal domain (CTD) Ser5 and Ser2 phosphorylation in HCC827/HCC4006ER and HCC827/HCC4006ER APE1 KD cells.

**Extended Data Fig.7│APE1 plays a crucial role in the formation of G4 structures in cells. a,** Confocal images immunostained with α-1H6 and APE1, counterstained with DAPI(nuclear), in erlotinib-resistant (ER) cells under the following conditions: control (untreated), PDS-treated (5 μM, 2 h), APE1 knockdown (KD), and APE1 KD + PDS (5 μM, 2 h), visualized by confocal microscopy (magnification: 63×; scale bars: 0.3 inches). **b and c,** Confocal images immunostained with α-1H6 and APE1, counterstained with DAPI, in ER cells treated with mitoxantrone (MX; 50 μM, 2 h), E3330 (APE1 inhibitor; 5 μM and 25 μM, 2 h). (magnification: 63×; scale bars: 0.3 inches).

**Extended Data Fig.8│APE1 stable AP site binding, not redox function is crucial for cellular G4 formation. a,** Expression levels of wild-type and mutant APE1 proteins were confirmed by Western blot. **b,** Representative confocal immunofluorescence images showing α-1H6 and APE1 staining, counterstained with DAPI (nuclear), in APE1 KD HCC4006 cells transfected with APE1 WT plasmid, redox-defective APE1 C65S/C93S plasmid, or repair-defective APE1 H309A plasmid for 48 h. (magnification, 63×; scale bars: 0.3 inches). **c,** Representative fields of view showing G4 foci formation detected by confocal microscopy in control, APE1 OE, APE1 OE + PDS (5 µM, 2 hours), and APE1 OE + MX (50 µM, 2 hours)-treated HCC4006 cells (magnification: 63×; scale bars: 0.3 inches).

**Extended Data Fig.9│APE1 modulates transcription of G4-mediated EMT- and stem-like related genes. a,** qRT-PCR analysis of EMT- and stemness-related gene expression (MAP3K4, CLDN2, ANKS1B, ESRP1, USP25, and KRT15) in control, APE1 OE, APE1 OE + PDS (5 μM), and APE1 OE + MX (50 μM) HCC4006 cells. Expression values normalized to GAPDH. P-values were determined using an unpaired Student’s t-test (****P < 0.0001, ***P < 0.001, **P < 0.01, *P < 0.05, ns. (non-significant) = P ≥ 0.05). Error bars denote ± SEM. Three independent experiments were performed in triplicate. **b,** Phalloidin staining visualized by confocal microscopy showing the number of actin stress fibers in control, APE1 OE, APE1 OE + PDS (5 µM), and APE1 OE + MX (50 µM)-treated HCC4006 cells. **c,** low cytometry analysis of cell surface CD133 expression (cancer stemness marker) in control, APE1 OE, APE1 OE + PDS (5 μM), and APE1 OE + MX (50 μM) HCC4006 cells. **d,** Western blot analysis of EMT- and stemness-related protein expression in control, APE1 OE, APE1 OE + PDS (5 μM), and APE1 OE + MX (50 μM) HCC4006 cells. **e,** Phase images (Left) and quantification data (Right) showed tumorsphere formation in parental cells and PDS treated HCC827/HCC4006 cells. Results are presented as mean ± SEM; n = 3; *P < 0.05, **P < 0.01, ***P < 0.001, independent t test. **f,** Phalloidin staining visualized by confocal microscopy showing the number of actin stress fibers in parental cells and PDS treated HCC827/HCC4006 cells. Results are presented as mean ± SEM; n = 3; *P < 0.05, **P < 0.01, ***P < 0.001, independent t test. **g,** Protein expression of EMT markers in parental cells and PDS treated HCC827/HCC4006 cells. Results are presented as mean ± SEM; n = 3; *P < 0.05, **P < 0.01, ***P < 0.001, independent t test.

**Extended Data Fig.10│G4 structures are found within hypomethylated CGIs in EMT- and stem-like related genes. a,** Venn diagram illustrating the overlap between the formation of G4 structures (represented by the BG4 peak) and the occurrence of CGIs in HCC827 (left) and HCC827ER (right) cells. **b,** Count of BG4 peaks overlapping a CGI in HCC827 (left) and HCC827ER (right) cells. **c,** Violin plot showing the size distribution of BG4 peaks and CGIs in HCC827 (left) and HCC827ER (right) cells. **d,** Histogram showing the distribution of GC percentages for the BG4 peak and CGIs in HCC827 (left) and HCC827ER (right) cells. **e,** Positional analysis of CGIs in gene promoter regions (NCBI RefSeq annotation) for EMT- and stemness-related genes (MAP3K4, CLDN2, ANKS1B, ESRP1, USP25, and KRT15). **f,** Consensus sequences of transcription factor binding sites (TFBSs) significantly enriched in differential BG4 peaks, identified using HOMER motif analysis software.

**Extended Data Fig.11│Downregulation of APE1 sensitizes ER cells to EGFR-TKI and suppresses xenograft tumor growth in vivo. a,** Representative IHC images demonstrating the expression levels of APE1, Vimentin, CD44, CLDN2 and ESRP1 in both treated and control tumors of initial xenograft (Scale bar, 50 µm).  **b,** Resected tumors after completion of treatment. Xenograft experiments using EGFR-TKI–sensitive parental HCC4006 cells to evaluate antitumor effects of PDS monotherapy and PDS–erlotinib combination treatment. Sixteen 4-week-old male BALB/c nude mice were randomized into 4 groups (n = 4 per group): HCC4006 + NS (normal saline), HCC4006 + erlotinib, HCC4006 + PDS, and HCC4006 + erlotinib + PDS. Mice were subcutaneously injected with 3 × 10⁶ cells in 100 μL PBS into the right flank. When tumor volumes reached ~150 mm³, treatment was initiated with NS, erlotinib, PDS, or erlotinib + PDS, respectively. **c,** Tumor volume was measured on indicated days, and the tumor growth curve was plotted. **d,** Resected tumors after completion of treatment. Experimental schematic for xenograft studies using APE1 KD in HCC4006ER cells. Sixteen 4-week-old male BALB/c nude mice were randomized into 2 groups (n = 8 per group): HCC4006ER + NC (negative control) and HCC4006ER + APE1 KD. Mice received subcutaneous injections of 3 × 10⁶ cells (100 μL PBS) into the right flank. At tumor volumes of 80–120 mm³, each group was further divided into NS and erlotinib treatment subgroups. Representative resected tumors shown after treatment completion. **e,** Tumor volume was measured on indicated days, and the tumor growth curve was plotted.
